# Carboxylic Acid Bioisosteres Boost Nurr1 Agonist Selectivity

**DOI:** 10.1021/acs.jmedchem.5c01140

**Published:** 2025-07-17

**Authors:** Tanja Stiller, Christian Gege, Wael Saeb, Jan Vietor, Úrsula López-García, Romy Busch, Hella Kohlhof, Daniel Vitt, Daniel Merk

**Affiliations:** 1 Ludwig-Maximilians-Universität München, Department of Pharmacy, 81377 Munich, Germany; 2 Immunic AG, 82166 Gräfelfing, Germany; 3 RebisLab R&D GmbH, 82152 Planegg-Martinsried, Germany

## Abstract

Nuclear receptor related 1 (Nurr1) is a neuronal ligand-activated transcription factor implicated in neurodegenerative diseases including Alzheimer´s disease, Parkinson´s disease and multiple sclerosis which has fueled the development of Nurr1 modulators. Among them, the clinically studied dihydroorotate dehydrogenase (DHODH) inhibitor vidofludimus was found to exhibit strong Nurr1 agonism. Here we aimed to establish a vidofludimus-derived Nurr1 agonist lacking DHODH inhibitor potency as tool. We explored bioisosteric replacement of the drug’s carboxylate motif and succeeded in boosting selectivity for Nurr1 over DHODH to >100-fold. Dopaminergic neural cells treated with the optimized tetrazole-based Nurr1 agonist revealed induction of genes involved in neuroprotection and neuronal health, supporting the potential of Nurr1 activation in neurodegenerative diseases.

## Introduction

**Chart 1:**
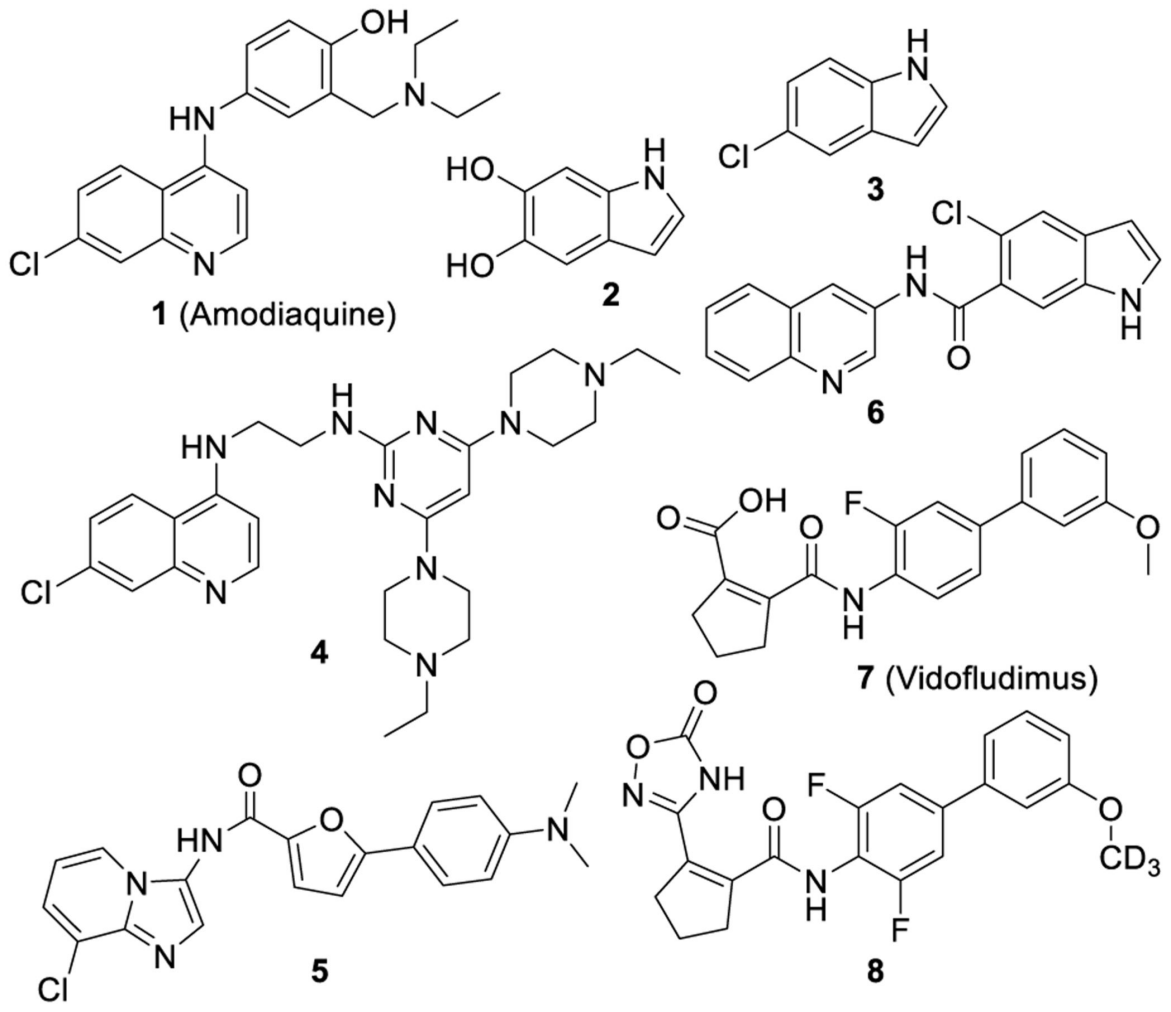
Nurr1 modulators

The ligand-sensing transcription factor nuclear receptor related 1 (Nurr1, NR4A2)^1^ exhibits a neuroprotective and anti-inflammatory role in the central nervous system (CNS) and is implicated in neurodegenerative diseases including Alzheimer´s disease (AD), Parkinson´s disease (PD) and multiple sclerosis (MS)^2–7^. Nurr1 agonists may thus have therapeutic potential in these pathologies and address urgent unmet medical needs. Substantial progress in Nurr1 agonist development has recently been made based on the antimalarial amodiaquine (**1**)^8–11^, which was the first reported synthetic Nurr1 ligand, based on the natural ligand 5,6-dihydroxyindole (**2**) and its mimetic **3**^12–15^, and based on fatty acid mimetics^16–18^ (Chart 1). Among the advanced agonists (**4-7**), **7** emerges with strong Nurr1 potency and activation efficacy (EC_50_ 0.4 µM, 3.1-fold activation)^17^. It is studied in late-stage clinical trials for multiple sclerosis^19^ and exhibits dihydroorotate dehydrogenase (DHODH) inhibition as another pharmacodynamic mode-of-action (IC_50_ 0.61 µM)^17^. In our endeavor to develop selective chemical tools for Nurr1 and the related NR4A receptors Nur77 and NOR1, we aimed to identify analogues of **7** with enhanced preference for Nurr1 over DHODH.

Previous structural analysis of DHODH inhibition by this chemotype^20^ revealed two possible binding modes in both of which the carboxylic acid motif engages in strong interactions and is bound in rather small polar subpockets suggesting that bulkier groups might diminish affinity. Significantly reduced DHODH inhibition of the analogue **8** (IC_50_ 9.26 µM)^21^ supports this assumption. For Nurr1 activation, the SAR of **7**^17^ also indicated importance of the carboxylic acid motif, but its structural variation has not been systematically explored yet. We hypothesized that bioisosteric replacement of the carboxylic acid in **7** might enable tuning of Nurr1 preference. Bioisosteres are considered as molecular metaphors that are primarily concerned with biological function rather than structure or physicochemical properties. They replace a functional group, that is important for a drug’s biological activity, and mimic the biological properties of this group but do not necessarily have to display similar steric and chemical features^22,23^. Bioisosteric relationships can hence exist between motifs that appear structurally quite different.

The carboxylic acid group frequently occurs in drug molecules and has the potential to engage strong polar contacts that contribute significantly to potency, but it can also negatively affect permeability and is susceptible to formation of reactive intermediates in phase 2 metabolism^24–26^. Several bioisosteric replacements have therefore been developed ranging from various heterocyclic motifs over sulfur and boron based acids to fluorinated alcohols^24–27^. They have enabled improvements in pharmacokinetic properties and safety of drugs but can also alter a drug molecule’s selectivity profile^25^ supporting potential of carboxylate bioisosteres to shift the preference of **7** towards Nurr1.

Here, we report a systematic exploration of bioisosteric analogues of **7**. Various carboxylic acid bioisosteres differently impacted on Nurr1 agonism and DHODH inhibition resulting in new selective and dual modulators. A tetrazole descendant (**32**) emerged as highly potent Nurr1 agonist with substantially improved preference over DHODH (>100-fold). Treatment of dopaminergic neurons with this compound resulted in strongly enhanced expression of genes associated with neuroprotection and neuronal health highlighting the potential of Nurr1 agonists in neurodegenerative diseases.

## Results & Discussion

**Chart 2:**
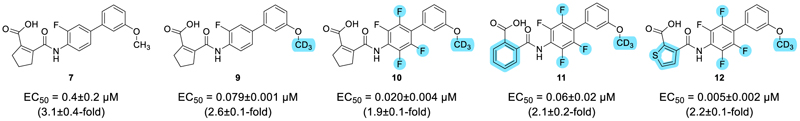
Design of the lead 12

Our previous studies on Nurr1 ligand discovery have identified **7** as potent agonist (EC_50_ 0.4 µM). Despite rather strict SAR of the scaffold for Nurr1 agonism, systematic evaluation revealed modifications in all substructures that enhanced potency (Chart 2): Deuteration of the methoxy group (**9**)^17^ or its extension to a prop-2-yn-1-yloxy group^17^ and introduction of further fluorine substituents on the central ring (**10**)^21^ were favored for Nurr1 agonist potency. Additionally, replacement of the cyclopentene residue by aromatic motifs was tolerated for benzene (**11**) and favored for thiophene (**12**). Compound **12** combining these modifications is a highly potent Nurr1 agonist and DHODH inhibitor (Table 1), and served as lead to explore the impact of carboxylic acid bioisosteres to improve Nurr1 selectivity.

We commenced the SAR evaluation by introducing common sulfonic acid-based motifs (Table 1). Replacement of the carboxylate (**12**) by a sulfonamide (**13**), an *N*-acetyl sulfonamide (**14**), or a methyl sulfone (**15**) markedly diminished Nurr1 agonism by more than 100-fold. Interestingly, all three analogues **13-15** and even the methylsulfonimidoyl derivative **16** exhibited equal Nurr1 agonist potency and efficacy, indicating that acidity of this motif was not a key factor for Nurr1 activation. **13-16** also displayed significantly reduced DHODH inhibitory potency, but with more distinctive profiles. While **13** and **14** comprising a protic group possibly acting as H-bond donor retained low micromolar DHODH inhibition, the methyl sulfone derivatives **15** and **16** were very weak DHODH inhibitors and thus gained in Nurr1 preference. Nevertheless, the overall weak potency of **13-16** prevented substantial SAR insights and further exploration.

Hence, we moved our attention to cyclic carboxylic acid bioisosteres and initially characterized the squaric acid (**17**), 1,2,4-oxadiazol-5(4*H*)-one (**18**), and tetrazole (**19**) analogues spanning a broad p*K*_a_ spectrum (Table 1). Despite being less active than the original carboxylic acid **12**, all three compounds exhibited markedly higher Nurr1 agonist potency than **13-16** and also retained stronger DHODH inhibition. The squaric acid **17** was least active and selective with respect to Nurr1. Both heterocyclic derivatives **18** and **19** displayed double digit nanomolar Nurr1 agonism and gained in selectivity for the nuclear receptor with 13- and 28-fold preference, respectively. Of note, **18** was almost equipotent to **19** despite two orders of magnitude lower acidity. These results indicated that heterocyclic carboxylic acid mimetics were favored to achieve potent Nurr1 agonism of the scaffold with enhanced preference over DHODH, and that shape and steric features might be more relevant than acidity.

Based on these encouraging observations for the 1,2,4-oxadiazol-5(4*H*)-one **18** and the tetrazole **19**, we explored further polar 5-membered heterocycles (Table 2). For ease of synthesis, we switched the main scaffold from the 2,3-disubstituted thiophene **12** to the symmetric 3,4-disubstituted analogue, which in the case of the carboxylic acid derivative **20** exhibited similar potency on both targets as **12**. The corresponding 1,2,4-oxadiazol-5(4*H*)-one (**21**) and tetrazole (**22**) analogues displayed similar double-digit nanomolar Nurr1 agonism as their counterparts **18** and **19** but Nurr1 preference was only retained in **21**. Hence, we focused on modifications of the 1,2,4-oxadiazol-5(4*H*)-one residue.

The 1,2,4-oxadiazole-5(4*H*)-thione **23** displaying slightly higher acidity was equipotent to **21** on Nurr1 but gained in DHODH inhibitory potency thus reducing preference. Methylation of **21** and **23** in **24** and **25** was detrimental for activity on Nurr1 and thus not productive, although **25** displayed enhanced Nurr1 preference. The alternative 1,3,4-oxadiazol-2(3*H*)-one regioisomer **26** exhibited strong potency on both Nurr1 and DHODH and hence no preference, and the bulkier thione analogue **27** comprised a similar profile with slightly lower potency. Only the rare 3*H*-1,2,3,5-oxathiadiazole 2-oxide **28** comprising a slightly larger ring size due to the sulfur atom displayed potent Nurr1 agonism with strong preference over DHODH (30-fold). Additionally, **28** exhibited favorably low lipophilicity and appeared promising for further profiling as a Nurr1 agonist tool.

The 3*H*-1,2,3,5-oxathiadiazole 2-oxide (**28**) and the tetrazole (**19, 22**) emerged as favorable replacements for the carboxylic acid in Nurr1 agonists based on **7**. Therein, the tetrazole tended to exhibit stronger Nurr1 activation efficacy (**22**), but minor differences in the scaffold (**19** vs. **22**) had a marked impact on the selectivity of the tetrazole derivatives due to substantially different DHODH inhibitory potency. Hence, we evaluated other related scaffolds for enhanced Nurr1 selectivity with the tetrazole moiety (Table 3). Previous SAR evaluation of the chemotype^17^ had revealed a dihydrofuran motif as another tolerated replacement for the cyclopentene of the original lead **7**. Its incorporation in the carboxylic acid lead **12** generated the potent Nurr1 agonist and DHODH inhibitor **29** but with pronounced preference for DHODH. Replacement of the carboxylic acid in this dihydrofuran scaffold by the tetrazole in **30** switched the selectivity towards 14-fold Nurr1 preference further underscoring the potential of this bioisoster. A similar trend was evident for the matched pair **11**/**31** bearing a simple benzene to replace the thiophene in **19**/**22** but with overall lower selectivity. Eventually, transfer of the favored tetrazole to the original lead **7** in **32** provided a remarkable improvement to >100-fold Nurr1 selectivity arising from 5-fold enhanced Nurr1 agonist potency and a substantial drop in DHODH inhibition.

**Figure 1:**
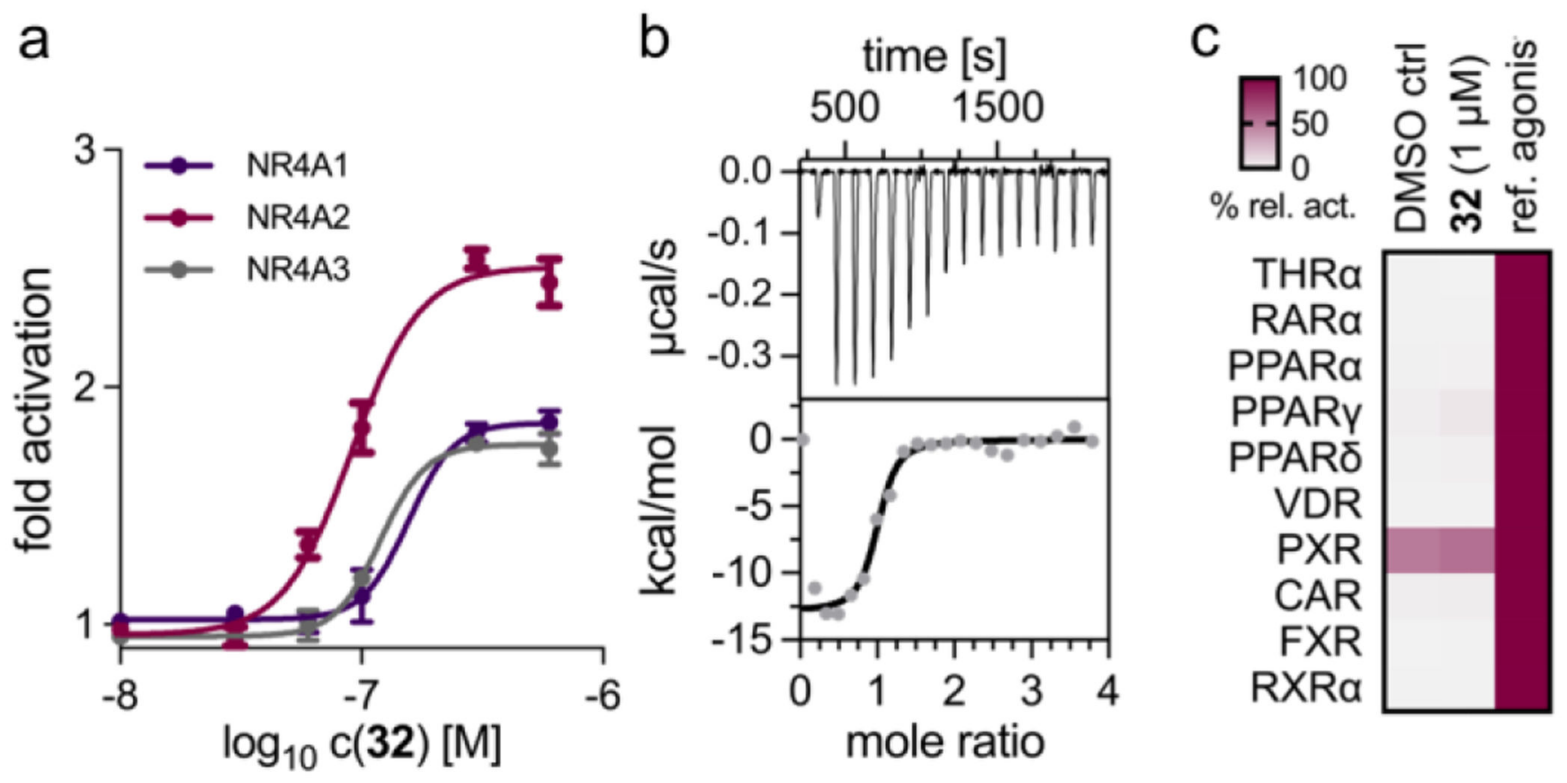
In vitro profiling of **32**. (a) Activity of **32** on NR4A receptors. Data are the mean±S.E.M. fold activation from Gal4 hybrid reporter gene assays; n ≥ 3. (b) Binding of **32** to the Nurr1 LBD in isothermal titration calorimetry (ITC) with a K_d_ value of 0.2 µM. The upper panel shows the isotherm of the **32**-protein titration; the lower panel shows the fitting of the heat of binding. (c) **32** revealed no relevant activity on lipid-sensing and promiscuous nuclear receptors at 1 µM. The heatmap shows the mean relative activation [%] compared to reference agonists.

Broad profiling of **32** (Figure 1) revealed a slight functional preference for Nurr1 activation (EC_50_ 0.09±0.01 µM, 2.5-fold act.) over the related nuclear receptors Nur77 (EC_50_ 0.16±0.03 µM, 1.9-fold act.) and NOR1 (EC_50_ 0.12±0.01 µM, 1.8-fold act.; Figure 1a), and the strong selectivity over DHODH was also retained in rats (64±2% rDHODH inhibition at 100 µM) suggesting suitability for in vivo studies. Moreover, **32** bound to the recombinant Nurr1 LBD with high affinity (K_d_ 0.2 µM) in isothermal titration calorimetry (Figure 1b). Outside the NR4A family of nuclear receptors, no activity was detected for **32** at 1 µM on other lipid-activated receptors (THR, RAR, PPAR, VDR, FXR, RXR) and promiscuous xenobiotic sensors (CAR, PXR) highlighting selectivity for NR4A (Figure 1c).

**Figure 2:**
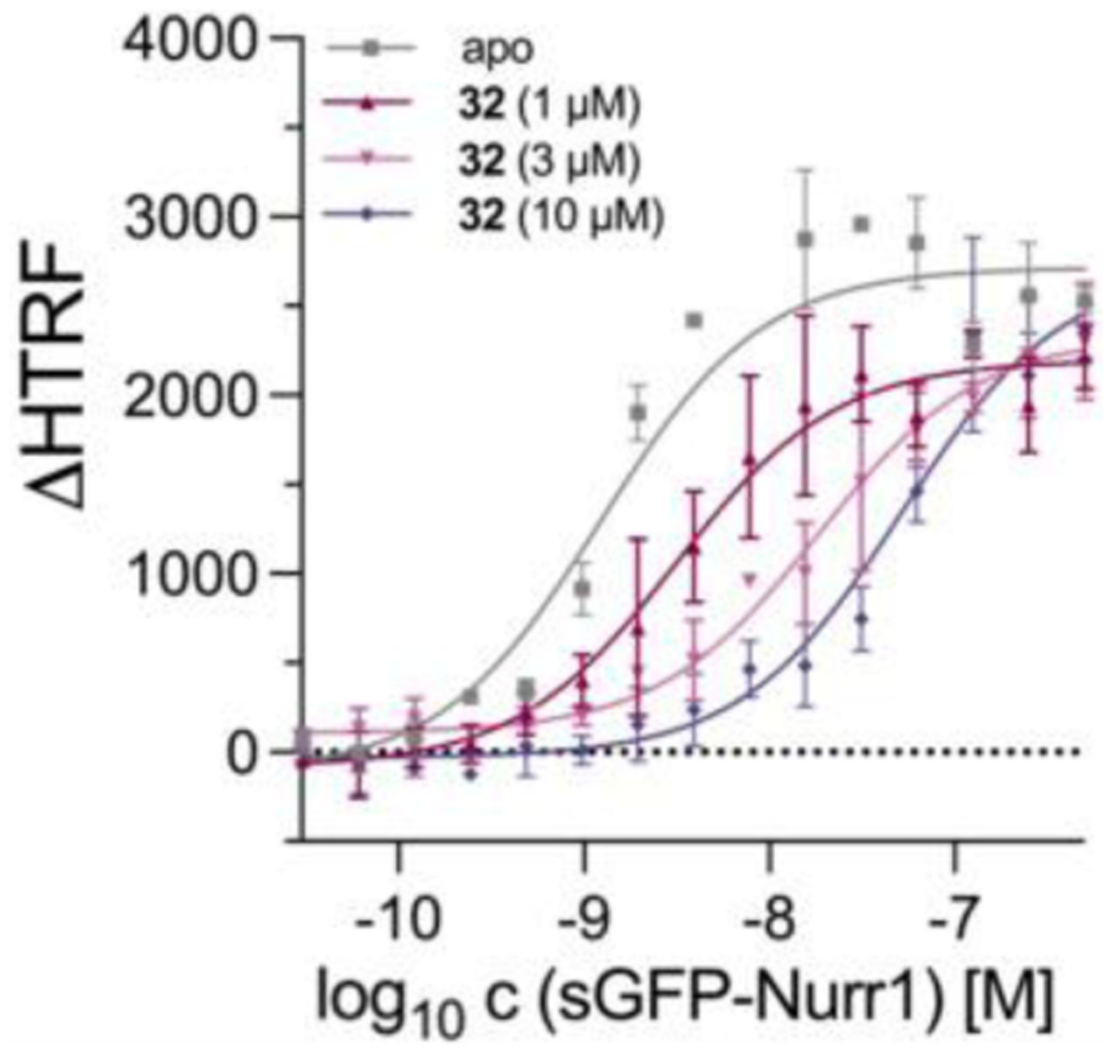
Impact of **32** on Nurr1 dimerization in a homogenous time-resolved fluorescence resonance energy transfer (HTRF) based assay monitoring the interaction of Tb^3+^-cryptate labeled Nurr1 LBD (FRET donor) and sGFP labeled Nurr1 LBD (FRET acceptor). Data are the mean±S.E.M. ΔHTRF; n = 3.

Next, we aimed to explore the mechanism by which **32** activated Nurr1. It has been hypothesized^32–34^ that agonists break Nurr1 dimers to release the transcriptionally more active Nurr1 monomer. Hence, we determined the impact of **32** on Nurr1 dimerization in a homogenous time-resolved fluorescence resonance energy transfer (HTRF) based assay monitoring the interaction of Tb^3+^-cryptate labeled Nurr1 LBD and sGFP labeled Nurr1 LBD (Figure 2). **32** robustly diminished Nurr1 dimer formation supporting this proposed activation mechanism.

**Figure 3:**
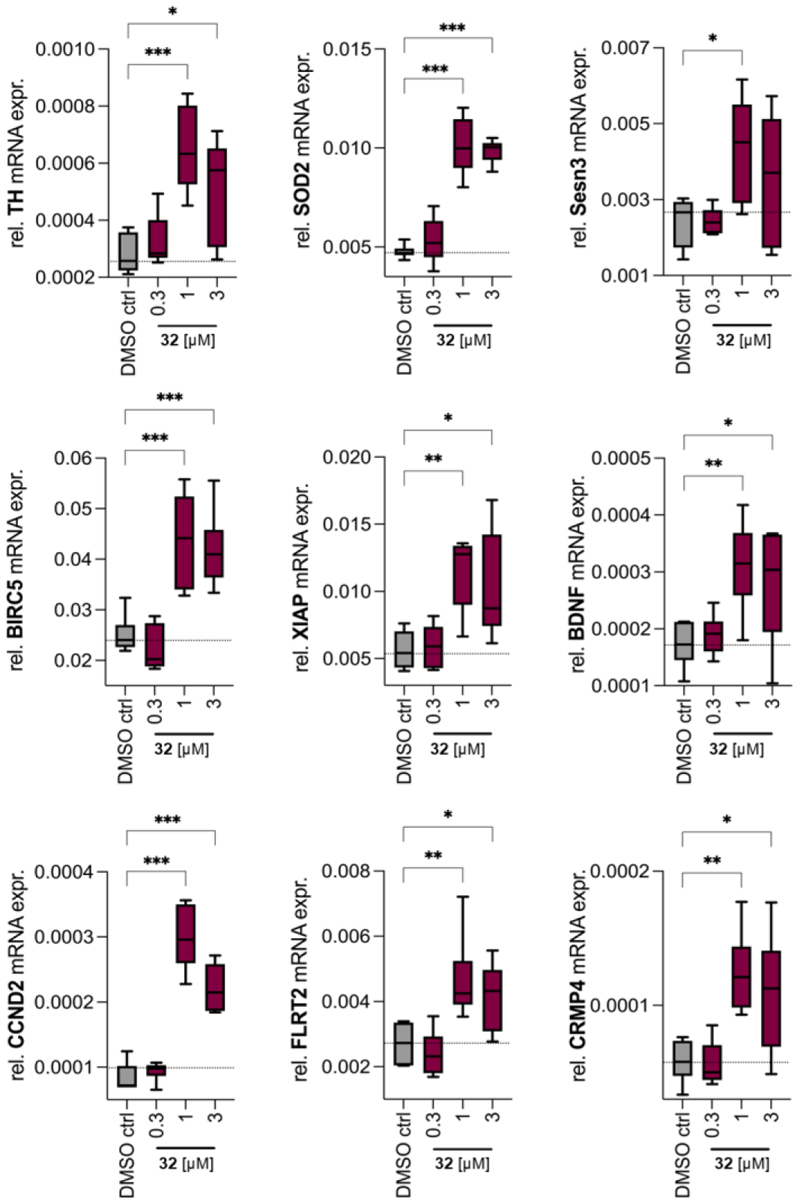
Effects of **32** on mRNA expression in rat dopaminergic neuronal cells (N27). TH−tyrosine hydroxylase, SOD2−superoxide dismutase 2, Sesn3−sestrin 3, BIRC5−baculoviral inhibitor of apoptosis repeat-containing 5 (also termed survivin), XIAP−X-linked inhibitor of apoptosis, BDNF−brain-derived neurotrophic factor, CCND2−cyclin D2, FLRT2−fibronectin leucine rich transmembrane protein 2, CRMP4−collapsin response mediator protein 4. mRNA levels were referenced to GAPDH and analyzed by the 2^-ΔCt^ method. Boxplots are min.-max.; n = 6; ^*^ p <0.05, ^**^ p <0.001, ^***^ p <0.0001 (ANOVA with Dunnett’s multiple comparisons test).

Nurr1 is considered as a neuroprotective factor particularly in dopaminergic neurons and as a promising therapeutic target for PD^6,10,35^. Therefore, we studied the effects of the optimized agonist **32** on gene expression in immortalized dopaminergic neuronal cells (N27, Figure 3). **32** enhanced the expression of the well-known Nurr1-regulated gene and dopaminergic neuron marker tyrosine hydroxylase (TH) demonstrating cellular target engagement. Additionally, the Nurr1 agonist mediated robust induction of superoxide dismutase 2 (SOD2)^36^ and sestrin 3 (Sesn3)^37^, both protecting from oxidative stress, and the antiapoptotic factors baculoviral inhibitor of apoptosis repeat-containing 5 (BIRC5)^38^ and X-linked inhibitor of apoptosis protein (XIAP)^39^. We also detected enhanced expression of brain-derived neurotrophic factor (BDNF), cyclin D2 (CCND2), fibronectin leucine rich transmembrane protein 2 (FLRT2) and collapsin response mediator protein 4 (CRMP4). FLRT2 is involved in neuronal guidance and repulsion of inappropriate synaptic partners^40,41^ whereas CRMP4 was reported to promote axonal regeneration, to regulate dendritic growth, and to be required for maturation and positioning of neurons^42–44^. Notably, genetic studies have suggested association of a missense mutation of CRMP4 with amyotrophic lateral sclerosis (ALS)^45^. Altogether, these gene expression effects suggest that activation of Nurr1 and possibly the related receptors Nur77 and NOR1 by **32** provide neuroprotection and improved neuronal health underscoring the potential of this mode-of-action in neurodegenerative diseases.

## Conclusion

Preclinical and clinical evidence of Nurr1 involvement in neurodegenerative diseases such as AD, PD and MS has fueled the development of Nurr1 agonists as potential therapeutics in neurodegeneration^2,4,6,7^. Vidofludimus (**7**) and analogues^17^ have emerged as potent and efficient Nurr1 activators and are among the best characterized and validated chemical tools to study the biology of Nurr1 as neuroprotective transcription factor. However, a vidofludimus-derived Nurr1 agonist with no DHODH inhibitor activity was lacking. Based on previous observations that carboxylic acid replacement in vidofludimus has strong impact on DHODH inhibitory potency, we studied carboxylic acid bioisosters for improved Nurr1 selectivity and identified common (tetrazole)^46^ and rare (3*H*-1,2,3,5-oxathiadiazole 2-oxide) bioisosters as highly favored. With strong Nurr1 agonist potency, validated direct and cellular target engagement, and >100-fold selectivity over DHODH, the tetrazole-based vidofludimus analogue **32** is a valuable next-generation Nurr1 agonist tool and enabled studies on Nurr1 agonist induced gene expression in vitro with no influence of residual DHODH inhibition. Treatment of dopaminergic neurons with **32** resulted in multifaceted upregulation of neuroprotective genes including protection from oxidative stress (SOD2, SESN3), anti-apoptotic factors (BIRC5, XIAP), and neuronal guidance/regeneration factors (FLRT2, CRMP4). These results suggest that Nurr1 agonism contributes to neuroprotective efficacy of vidofludimus and further underscore potential of Nurr1 activation as therapeutic concept in neurodegeneration.

### Chemistry

**Scheme 1:**
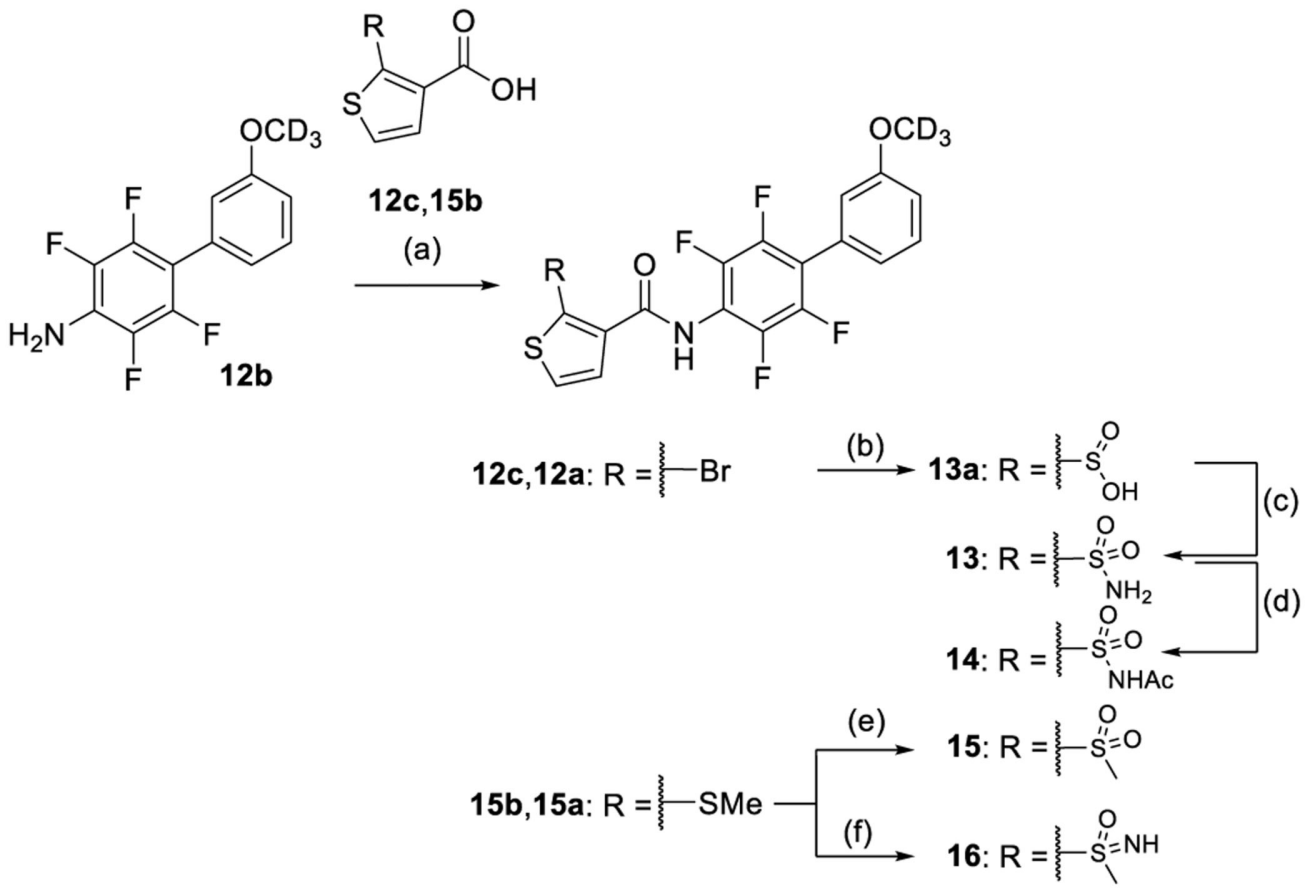
Synthesis of 13–16.^a^ ^a^ Reagents&Conditions: (a) **12c, 15b** with oxalyl chloride, 0°C to rt, 2 h, evap.; NaH, THF, 10 min, 0°C, then acid chloride, 0°C to rt, 6 h, 49%; (b) *n*-BuLi (2.5 M in THF), THF, –78°C, 30 min, then 1,4-diazabicyclo[2.2.2]octane sulfur dioxide complex, –78°C, 30 min, 23%; (c) hydroxylamine-*O*-sulfonic acid, NaOAc, MeCN/H_2_O (1:1), rt, 16 h, 36%; (d) cat. ZnCl_2_, Ac_2_O, rt, 1 h, 50 %; (e) *m*-CPBA, CH_2_Cl_2_, rt, 1 h, 8%; (f) (NH_4_)_2_CO_3_, (diacetoxyiodo)benzol, MeOH, rt, overnight, 24%.

Compounds **13-16** were prepared by reacting aniline **12b** with the corresponding carboxylic acids **12c** and **15b** in an amide coupling reaction to obtain building blocks **12a** and **15a**, followed by subsequent treatment according to Scheme 1. Treatment of **12a** with *n*-BuLi in THF and then 1,4-diazabi-cyclo[2.2.2]octane sulfur dioxide complex yielded sulfinic acid **13a**, followed by reaction with hydroxylamine-*O*-sulfonic acid to obtain sulfonamide **13**. Treatment of the sulfonamide **13** with acetic anhydride in the presence of a catalytic amount of ZnCl_2_ afforded *N*-acetyl sulfonamide **14**. Oxidation of intermediate **15a** with *m*-CPBA in DCM yielded methyl sulfone derivative **15**, while reaction with ammonium carbonate in the presence of (diacetoxyiodo)benzol afforded the methylsulfonimidoyl derivative **16**.

**Scheme 2:**
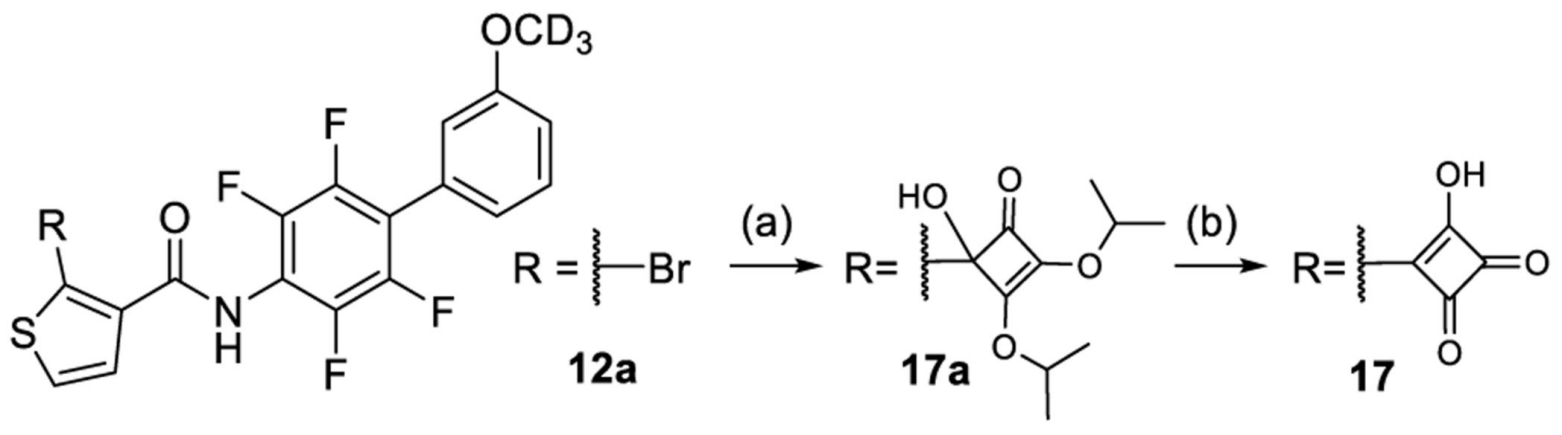
Synthesis of 17 ^a^ Reagents&Conditions: (a) *n*-BuLi (2.5 M in THF), THF, –78°C, 30 min, then 3,4-diisopropoxycyclobut-3-ene-1,2-dione, –78°C, 30 min, 40%; (b) AcOH/1N aq. HCl (6:1), rt, overnight, 32%.

The squaric acid analogue **17** was prepared by treating the aryl bromide precursor **12a** with *n*-BuLi and 3,4-diisopropoxy-cyclobut-3-ene-1,2-dione to afford intermediate **17a**, and cleavage of the isopropyl ethers yielded **17** (Scheme 2).

**Scheme 3:**
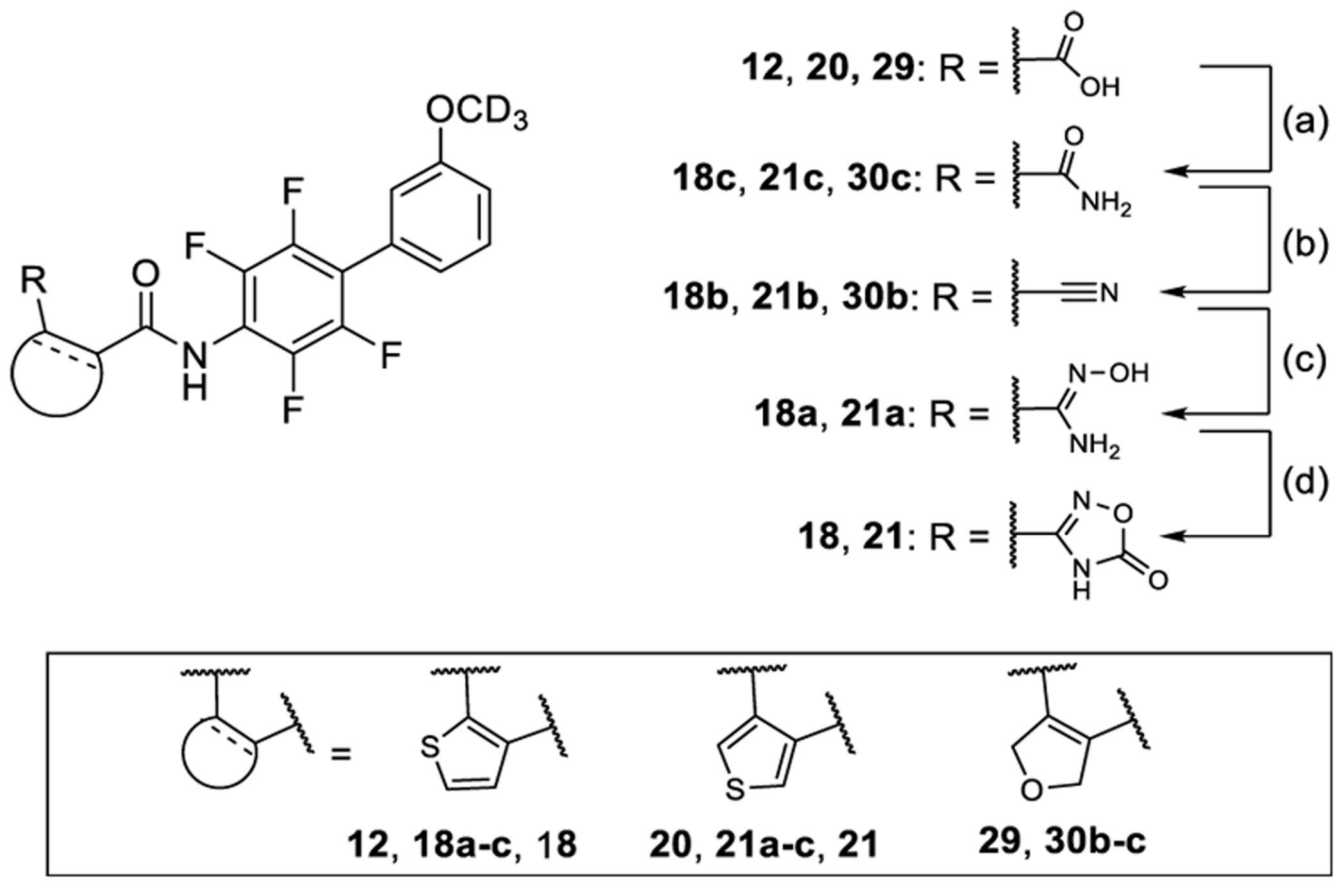
Synthesis of 18 and 21.^a^ ^a^ Reagents&Conditions: (a) NH_4_Cl, EDC, DMAP, CH_2_Cl_2_ rt to 60°C, 3 h, 80% (**18c**), NH_4_Cl, EDC, DMAP, DMF, rt to 60°C, overnight, 50–52% (**21c, 30c**); (b) cyanuric chloride, DMF, 0°C, 3 h, 70–78% (**18b, 30b**), Burgess reagent, THF, 0°C to rt, 94% (**21b**); (c) H_2_NOH•HCl, Hünig's base, MeOH, 70°C, 4 h, 46% (**18a**), H_2_NOH, EtOH/H_2_O, 50°C, 5 h, 88% (21**a**); (d) CDI, 1,8-diaza-bicyclo[5.4.0]undec-7-en, 1,4-dioxane, 100°C, 3 h, 36% (**18**), CDI, DMF, 100°C, 15 h, 21% (**21**).

The carboxylic acid derivatives **9, 10, 12, 20** and **29** were prepared as described previously^21^. The 1,2,4-oxadiazol-5(4*H*)-one analogues **18** and **21** were prepared from **12** and **20** over four steps according to Scheme 3. Treatment of **12** with ammonium chloride in the presence of EDC and 4-DMAP in DCM yielded primary amide **18c**. Carboxylic acid **20** treated with ammonium chloride in the presence of EDC and 4-DMAP in DMF afforded primary amide **21c**. Amides **18c** and **21c** were then treated with cyanuric chloride (**18c**) or Burgess reagent (**21c**) to obtain the nitriles **18b** and **21b**. The *N*-hydroxy amidines **15a** and **18a** were prepared by reacting the corresponding nitriles **18b** and **21b** with hydroxylammonium chloride and Hünig's base (**18a**) or hydroxylamine (**21a**). Cycloaddition of **18a** with carbonyldiimidazole in the presence of DBU in 1,4-dioxane afforded **18**. Treatment of **21a** with carbonyldiimidazole in DMF yielded **21**.

**Scheme 4:**
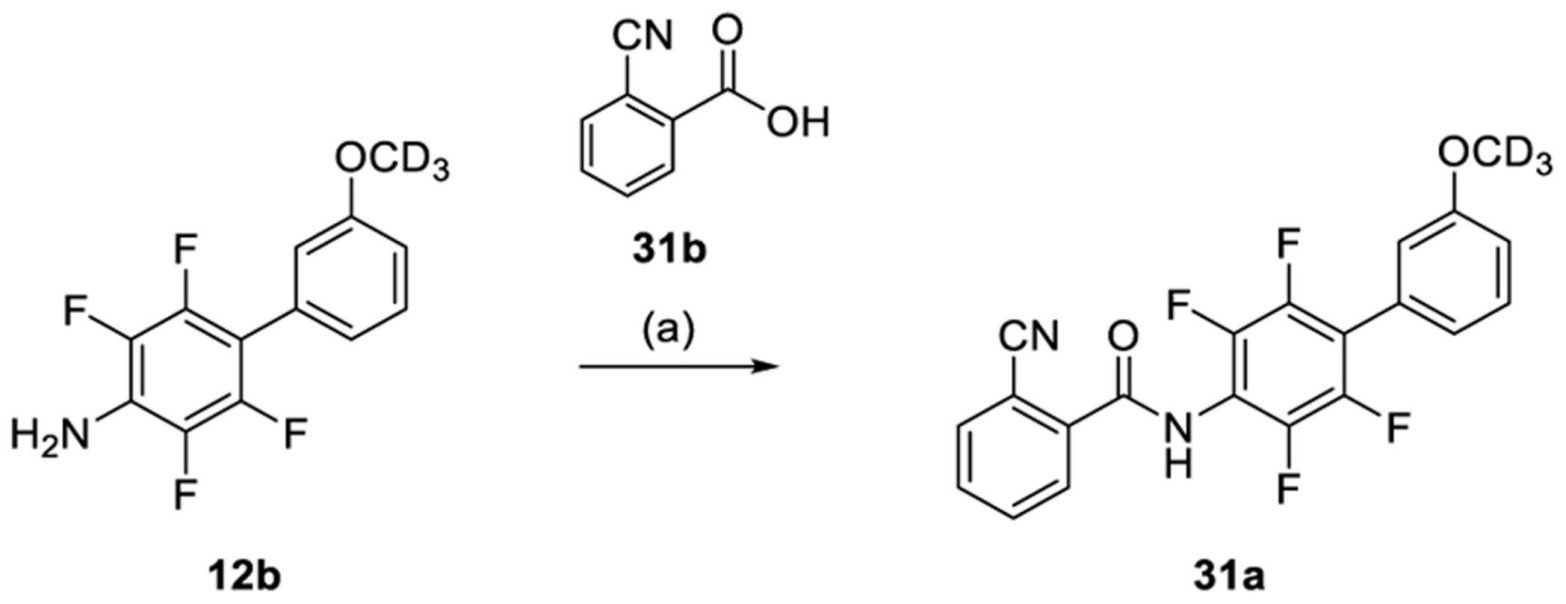
Synthesis of Building Block 31a.^a^ ^a^ Reagents&Conditions: (a) **31b**, SOCl_2_, CH_2_Cl_2_, 0°C, 2 h, evap.; **12b**, NaH, THF, 0°C, 30 min, then acid chloride, 0°C, 2h, 18%.

The benzonitrile precursor **31a** was obtained from **12b** according to Scheme 4 by amide coupling with carboxylic acid **31b**.

**Scheme 5:**
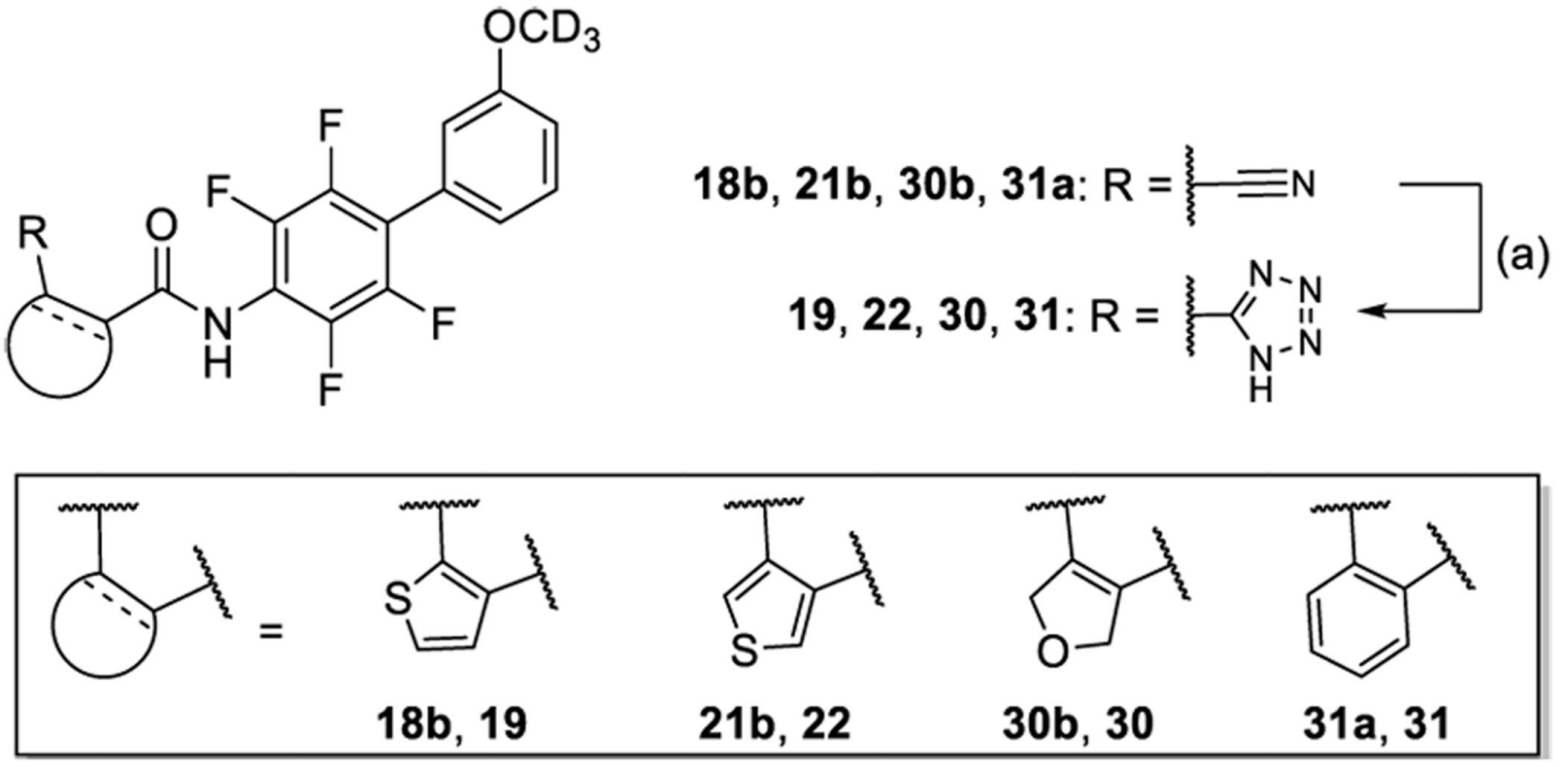
Synthesis of 19, 22, 30 and 31.^a^ ^a^ Reagents&Conditions: (a) NaN_3_, NH_4_Cl, DMF, 125°C, overnight, 25–46% (**19, 22, 30**), NaN_3_, Et_3_N•HCl, DMF, 120°C, 4 h, 11% (**31**).

The 1*H*-tetrazole derivatives **19, 22, 30** and **31** were prepared by reacting the corresponding nitriles **18b, 21b, 30b** and **31a** with sodium azide according to Scheme 5.

**Scheme 6:**
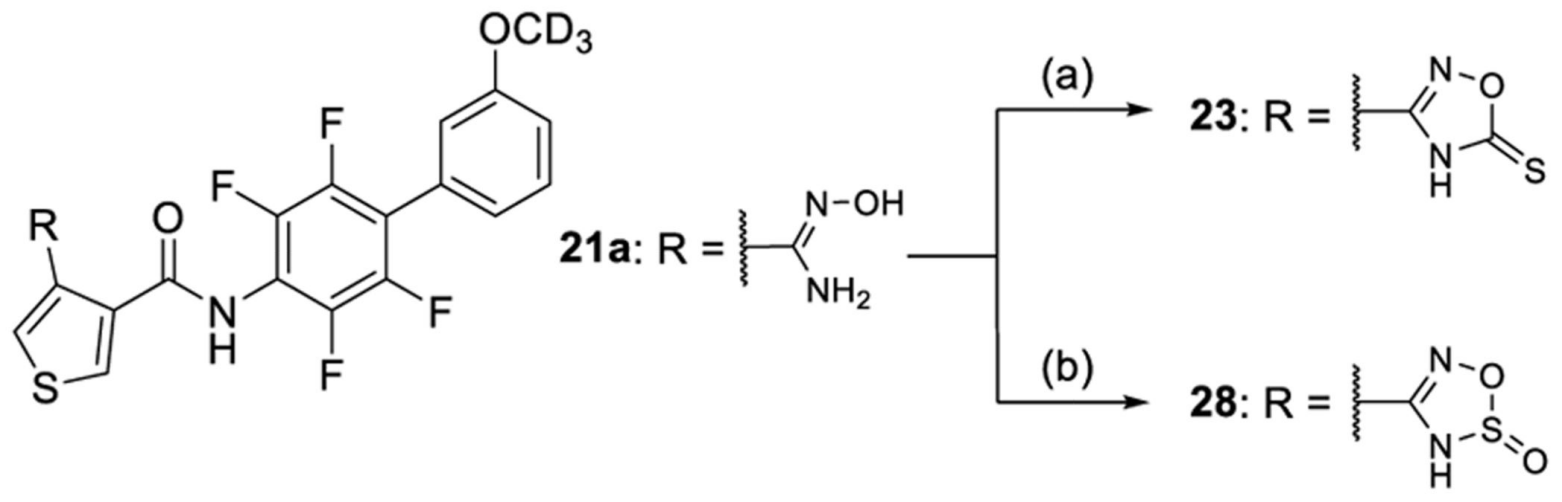
Synthesis of 23 and 28.^a^ ^a^ Reagents&Conditions: (a) 1,1'-thiocarbonyldiimidazole, Hünig's base, DMF, 100°C, overnight, 33%; (b) SOCl_2_, pyridine, CH_2_Cl_2_, −50°C to rt, 2 h, 29%.

Synthesis of the 1,2,4-oxadiazole-5(4*H*)-thione derivative **23** was achieved by reacting *N*-hydroxyamidine **21a** with 1,1'-thiocarbonyldiimidazole and Hünig's base, and treatment of **21a** with SOCl_2_ and pyridine afforded the corresponding 3*H*-1,2,3,5-oxathiadiazole 2-oxide analogue **28** (Scheme 6).

**Scheme 7:**
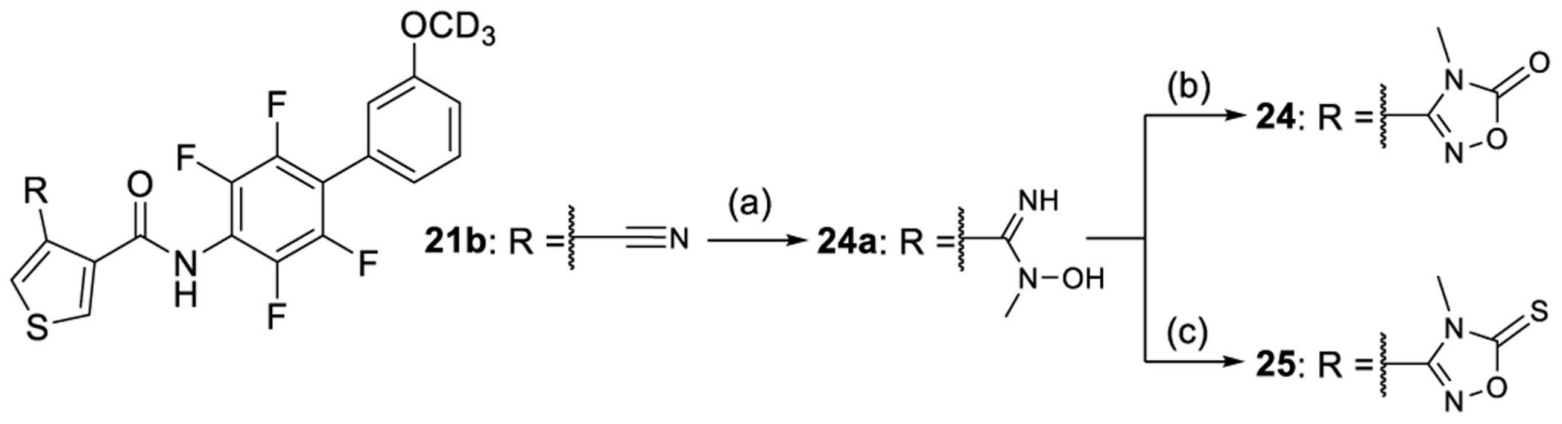
Synthesis of 24 and 25.^a^ ^a^ Reagents&Conditions: (a) MeHNOH•HCl, Hünig's base, EtOH, 80°C, 5 h, 54%; (b) 1,1'-carbonyldiimidazole, Hünig's base, DMF, 80°C, overnight, 62%; (c)1,1′-thiocarbonyldiimidazole, Hünig's base, DMF, 80°C, 15 h, 60%.

Similarly, reaction of the nitrile **21b** with MeHNOH•HCl and Hünig's base to intermediate **24a** and subsequent treatment of **24a** with 1,1'-carbonyldiimidazole and Hünig's base afforded *N*-methyl-1,2,4-oxadiazol-5(2*H*)-one **24**, while reaction of **24a** with 1,1′-thiocarbonyldiimidazole in the presence of Hünig's base yielded the *N*-methyl-1,2,4-oxadiazole-5(2*H*)-thione **25** (Scheme 7).

**Scheme 8:**
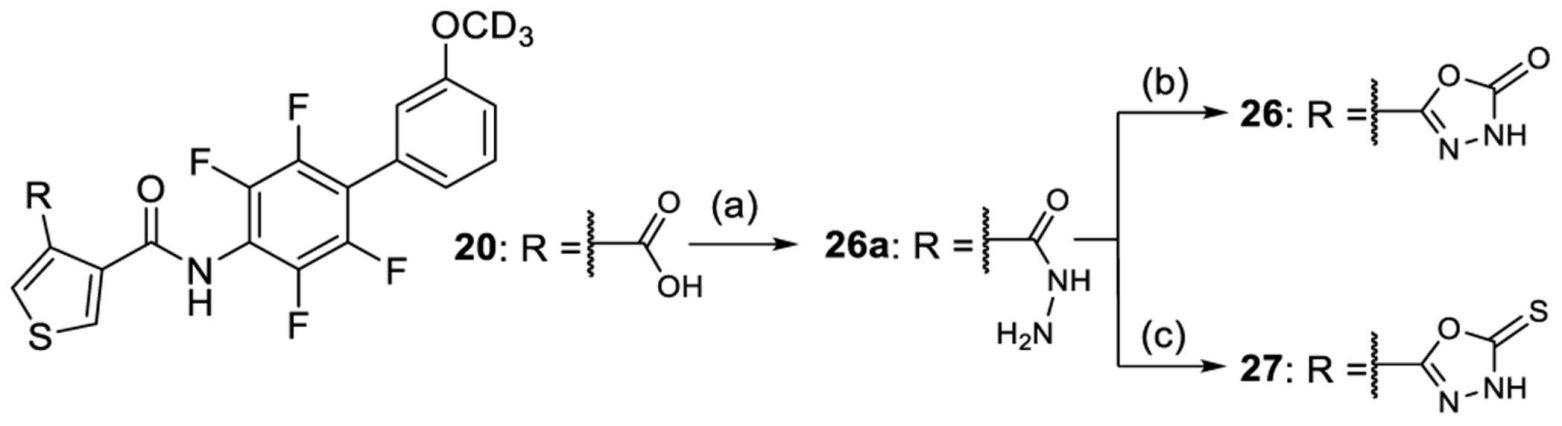
Synthesis of 26 and 27.^a^ ^a^ Reagents&Conditions: (a) oxalyl chloride, CH_2_Cl_2_, cat. DMF, 0°C to 50°C, 1 h, evap., then NH_2_NH_2_•H_2_O, Hünig's base, THF, rt, overnight, 49%; (b) 1,1'-carbonyldiimidazole, Hünig's base, DMF, rt, 4 h, 35%; (c) 1,1′-thiocarbonyldiimidazole, Hünig's base, DMF, 80°C, 15 h, 60%.

The isomeric 1,3,4-oxadiazole derivatives **26** and **27** were prepared by reacting the carboxylic acid **20** with oxalyl chloride in DCM with a catalytic amount of DMF, followed by treatment with hydrazine hydrate and Hünig's base in THF to afford intermediate **26a**. Cycloaddition of **26a** with 1,1'-carbonyldiimidazole and Hünig's base yielded 1,3,4-oxadiazol-2(3*H*)-one **26**, while reaction of **26a** with 1,1′-thiocarbonyldiimidazole and Hünig's base afforded 1,3,4-oxadiazol-2(3*H*)-thione **27**. (Scheme 8).

**Scheme 9:**
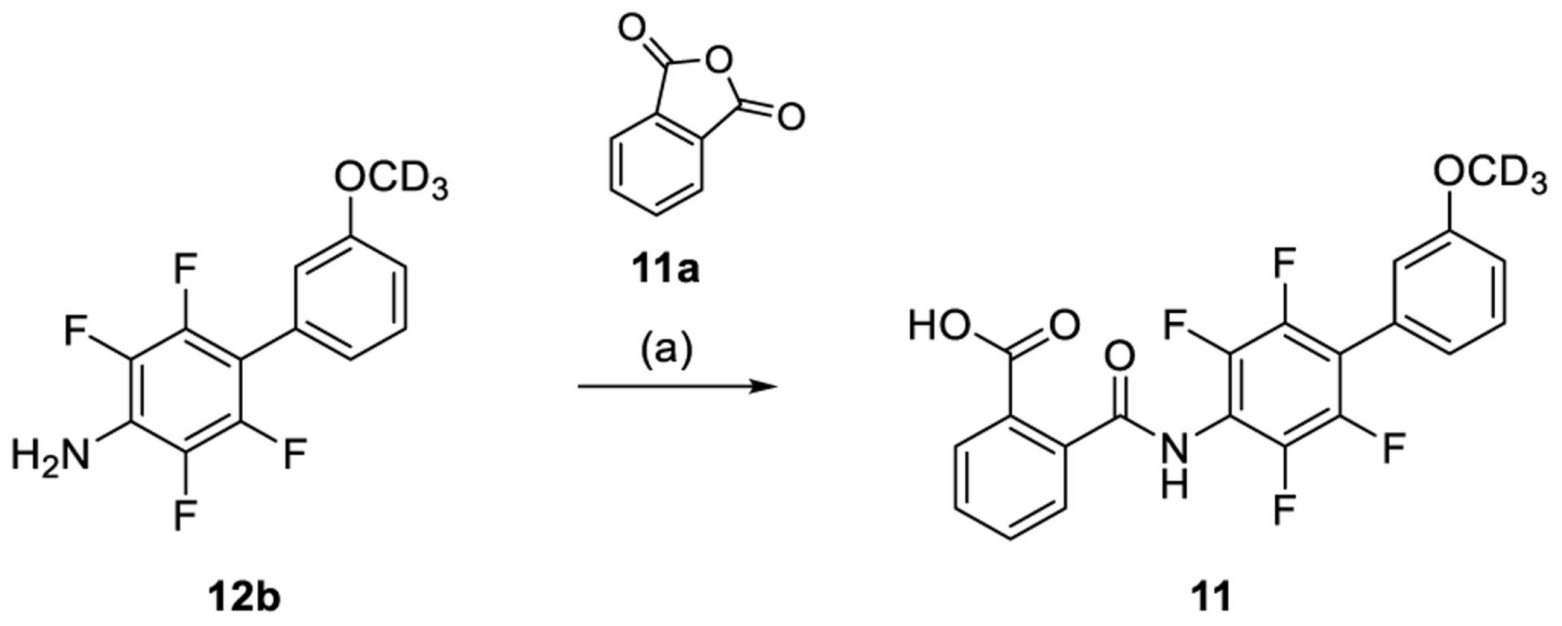
Synthesis of 11.^a^ ^a^ Reagents&Conditions: (a) isobenzofuran-1,3-dione **11a**, AlCl_3_, CHCl_3_, 70°C, 12 h, 50%.

The benzoic acid **11** was prepared by reacting aniline **12b** with isobenzofuran-1,3-dione **11a** according to Scheme 9.

**Scheme 10:**
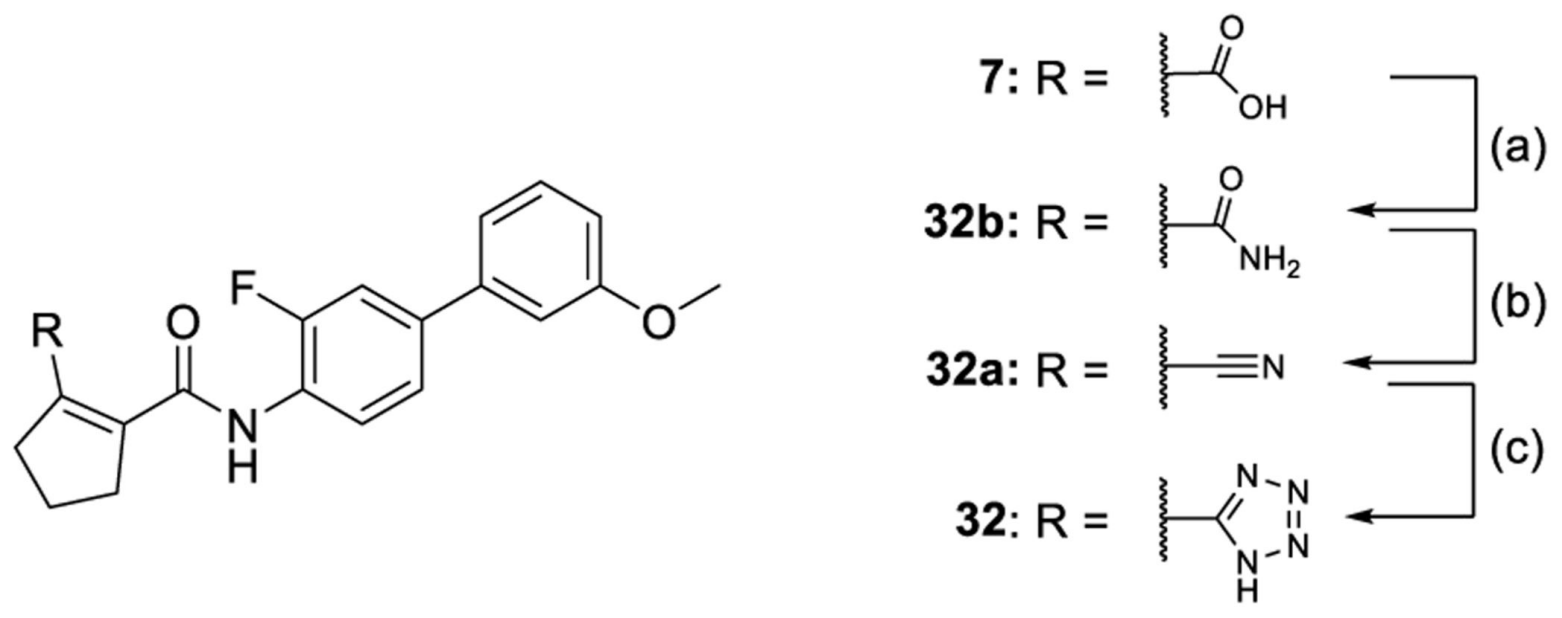
Synthesis of 32.^a^ ^a^ Reagents&Conditions: (a) oxalyl chloride, CH_2_Cl_2_, cat. DMF, 0°C to 50°C, 1 h, evap., then NH_3_•H_2_O, THF, 5°C to rt, overnight, 81%; (b) Burgess reagent, THF, 0°C to rt, 1 h, 81%; (c) NaN_3_, NH_4_Cl, DMF, 120°C, 15 h, 9%.

The tetrazole **32** was prepared from the corresponding carboxylic acid **7** according to Scheme 10 by treatment with oxalyl chloride in DCM with a catalytic amount of DMF followed by reaction with NH_3_•H_2_O in THF to obtain the primary amide **32b**. Amide **32b** was then treated with Burgess reagent to obtain the nitrile **32a**, and cycloaddition with sodium azide in the presence of ammonium chloride in DMF afforded **32**.

### Experimental Procedures

#### Chemistry

*General* All chemicals were of reagent grade, purchased from commercial sources and used without further purification unless otherwise specified. All reactions were conducted in oven-dried glassware under Ar or N_2_ atmosphere and in absolute solvents (where appropriate). Other solvents, especially for work-up procedures, were of reagent grade. Compounds were purified with a Biotage Isolera One combiflash chromatography system (SEPAFLASH, 40-63Å) or Büchi Reveleris Prep system with FlashPure cartridges (EcoFlex Silica 50µm irregular 80 g, Reveleris HP Silica 20µm 40 g or Reveleris HP Silica 20µm 12 g) and with the solvent mixtures specified in the corresponding experiment. Preparative HPLC was performed using a combiflash reversed-phase chromatography (C18) Boston ODS 40 g Flash 35mL–50mL/min at 200 psi with gradient A: 0.1% NH_4_HCO_3_ in water, 10–100% MeCN, or with gradient B: 0.1% TFA in water, 10–100% MeCN. Alternatively, preparative HPLC was performed on a Büchi Pure C-850 FlashPrep system and C18 column [10 µm, 250 x 10.0 ID mm or and 150 x 30.0 ID mm] using gradient C: 5–90% MeOH gradient in water. Mass spectra were obtained on a puriFlash^®^-CMS system (Advion) using atmospheric pressure chemical ionization (APCI), an Ion Trap Esquire 3000+ instrument (Bruker Corporation, Billerica, MA, USA) using electrospray ionization (LCMS (ESI)) or using a *Waters Acquity SQ Detector* coupled to a *Waters Acquity* UPLC system. The instrument was operated in electrospray ionization (ESI) mode in both positive and negative ionization modes. Data acquisition and processing were performed using Waters MassLynx software. Using a [BEH C18, 1.7 µm, 2.1 × 50 mm], with a mobile phase of [water/acetonitrile + 0.1% formic acid] at a flow rate of [0.5 mL/min], gradient: 2% - 98% B in 6.0 min; oven temperature: 10°C; mass range: 110-1000; detection: UV (190 - 400 nm) and MS (ESI, Pos mode, 2 to 2000 Da (amu)). NMR spectra were recorded on Bruker Avance 300 MHz, 400 MHz or 500 MHz spectrometers equipped with CryoProbe™ Prodigy broadband probe (Bruker) or a Magritek Spinsolve 80 spectrometer (80 MHz). Chemical shifts are reported in δ values (ppm), coupling constants (*J*) in Hertz (Hz). Signals are described as br for broad. Purity of all compounds was analyzed on an Agilent Technologies 1200 Series machine under the following conditions: LC-Mass Method 1: column: Sunfire C18, 4.6*50 mm, 3.5 µm; mobile phase: A: water (0.01% TFA), B: MeCN (0.01% TFA); gradient: 5% - 95% B in 1.5 min; flow rate: 2.0 mL/min; oven temperature: 50°C; mass range: 110-1000; detection: UV (214 nm, 254 nm) or LC-Mass Method 2: column: Xbridge C18(2) (4.6*50 mm, 3.5 µm); mobile phase: A: H_2_O (10 mmol NH_4_HCO_3_), B: MeCN; elution program: gradient from 10 to 95% of B in 1.5 min at 1.8 mL/min; temperature: 50 ºC; detection: UV (214 nm, 254 nm) and MS (ESI, Pos mode,103 to 800 amu). CD_3_I used for deuteration had ≥99% isotopic purity, the deuterated products had ≥98% isotopic purity according to NMR. All compounds for biological testing had a purity >95% based on the 254 nm UV-trace.

#### 2-{[2,3,5,6-Tetrafluoro-3’-(^2^H_3_)methoxy-[1,1'-biphenyl]-4-yl]carbamoyl}benzoic acid (11)

2,3,5,6-tetrafluoro-3’-(^2^H_3_)methoxy-[1,1'-biphenyl]-4-amine (**12b**, 1.2 g, 4.7 mmol) and AlCl_3_ (0.61 g, 4.6 mmol) were added to a stirred solution of isobenzofuran-1,3-dione (**11a**, 0.70 g, 4.7 mmol) in CHCl_3_ (10 mL). The mixture was stirred at 70°C for 12 h, cooled down to rt, concentrated and purified by preparative HPLC (gradient B) to afford compound **11** (1.0 g, yield: 50%) as a colorless solid. ^1^H-NMR (400 MHz, DMSO-*d*_6_) δ = 13.21 (br s, 1H), 10.70 (s, 1H), 7.91 (d, *J* = 7.6 Hz, 1H), 7.73 (m, 3H), 7.48 (t, *J* = 8.0 Hz, 1H), 7.15–7.08 (m, 3H) ppm. ^13^C-NMR (126 MHz, DMSO-*d*_6_) δ =167.9, 167.8, 159.8, 143.9 (m), 142.8 (m), 137.5, 132.2, 130.9, 130.5, 130.4, 130.1, 128.5, 128.2, 122.7, 118.0 (t, *J* = 17.5 Hz), 116.8 (m), 116.2, 115.4, 54.9 (m) ppm. LCMS (ESI): *m/z* 421.1 ([M–H]^–^).

#### 2-Bromo-*N*-{2,3,5,6-tetrafluoro-3'-(^2^H_3_)methoxy-[1,1'-biphenyl]-4-yl}thiophene-3-carboxamide (12a)

Oxalyl chloride (50 mL) was added to 2-bromothiophene-3-carboxylic acid (**12c**, 5.0 g, 2.4 mmol) at 0°C. The mixture was then stirred at rt for 2 h and concentrated under *vacuo* to afford the crude acid chloride intermediate. NaH (5.5 g, 60%wt) was added to a solution of 2,3,5,6-tetrafluoro-3'-(^2^H_3_)methoxy-[1,1'-biphenyl]-4-amine (**12b**, 6.6 g, 2.4 mmol) in anhydrous THF (50 mL). The mixture was then stirred at 0°C for 10 min. The acid chloride intermediate was added subsequently, and the mixture was stirred at rt for 6 h, quenched with saturated aq. NH_4_Cl and extracted with EtOAc (3 × 150 mL). The combined organic layer was dried over Na_2_SO_4_, filtered, concentrated and purified by preparative HPLC (gradient B) to obtain compound **9a** (5.5 g, yield: 49%) as a colorless solid. LCMS (ESI): *m/z* 465.0 ([M+H]^+^).

#### Sulfamoyl-*N*-{2,3,5,6-tetrafluoro-3'-(^2^H_3_)methoxy-[1,1'-biphenyl]-4-yl}thiophene-3-carboxamide (13)

NaOAc (73 mg, 8.9 mmol) and hydroxylamine-*O*-sulfonic acid (0.20 g, 1.8 mmol) were added to a stirred solution of compound **13a** (0.40 g, 0.89 mmol) in MeCN/H_2_O (1:1, 20 mL). The mixture was stirred at rt for 16 h, concentrated and purified by preparative HPLC (gradient A) to obtain compound **13** (0.15 g, yield: 36%) as a colorless solid. ^1^H-NMR (400 MHz, CD_3_OD) δ = 7.70 (d, *J* = 5.2 Hz, 1H), 7.61 (d, *J* = 5.2 Hz, 1H), 7.46–7.41 (m, 1H), 7.07– 7.04 (m, 3H) ppm. ^13^C-NMR (126 MHz, DMSO-*d*_*6*_) δ = 161.2, 159.3, 147.8, 143.4 (m), 141.4 (dt, *J* = 15.9, 3.7 Hz), 134.1, 129.9, 129.7, 128.9, 127.6, 122.2, 118.2 (t, *J* = 17.7 Hz), 115.8, 115.0, 54.5 (m) ppm. LCMS (ESI): *m/z* 464.0 ([M+H]^+^).

#### 3-{[2,3,5,6-Tetrafluoro-3'-(^2^H_3_)methoxy-[1,1'-biphenyl]-4-yl]carbamoyl}thiophene-2-sulfinic acid (13a)

To a stirred solution of compound **12a** (2.0 g, 4.3 mmol) in anhydrous THF (25 mL) *n*-BuLi (2.5M in THF, 1.7 mL) was added at –78°C. The mixture was stirred at –78°C for 30 min. and 1,4-diazabicyclo[2.2.2]octane sulfur dioxide complex (0.52 g, 2.4 mmol) was then added. The mixture was stirred for another 30 min at –78°C, quenched with water and purified by preparative HPLC (gradient A) to give compound **13a** (0.45 g, yield: 23%) as a colorless solid. LCMS (ESI): *m/z* 447.0 ([M–H]^–^).

#### 2-(*N*-Acetylsulfamoyl)-*N*-{2,3,5,6-tetrafluoro-3’-(^2^H_3_)methoxy-[1,1'-biphenyl]-4-yl}thiophene-3-carboxamide (14)

ZnCl_2_ (3 mg) was added to a stirred solution of compound **13** (0.10 g, 0.22 mmol) in Ac_2_O (1 mL). The mixture was stirred at rt for 1 h, concentrated and purified by preparative HPLC (gradient A) to afford compound **14** (55 mg, yield: 50%) as a colorless solid. ^1^H-NMR (400 MHz, CD_3_OD) δ = 7.75 (d, *J* = 5.2 Hz, 1H), 7.43–7.40 (m, 2H), 7.06–7.04 (m, 3H), 1.97 (s, 3H) ppm. ^13^C-NMR (126 MHz, DMSO-*d*_*6*_) δ = 160.8, 159.3, 143.4 (m), 141.1 (m), 129.9, 129.1, 127.8, 122.3, 116.9 (m), 115.8, 114.9, 54.5 (m), 25.6 (m) ppm. LCMS (ESI): *m/z* 504.1 ([M–H]^–^).

#### 2-(Methylsulfonyl)-*N*-{2,3,5,6-tetrafluoro-3’-(^2^H_3_)methoxy-[1,1'-biphenyl]-4-yl}thiophene-3-carboxamide (15)

*m*-CPBA (0.14 g, 0.79 mmol) was added to a stirred solution of compound **15a** (0.17 g, 0.39 mmol) in CH_2_Cl_2_ (2 mL). The mixture was stirred at rt for 1 h, concentrated and purified by preparative HPLC (gradient A) to obtain compound **15** (15 mg, yield: 8%) as a colorless solid. ^1^H-NMR (500 MHz, CD_3_OD) δ = 8.01 (d, *J* = 5.0 Hz, 1H), 7.60 (d, *J* = 5.0 Hz, 1H), 7.44 (t, *J* = 7.8 Hz, 1H), 7.07–7.04 (m, 3H), 3.50 (s, 3H) ppm. ^13^C-NMR (126 MHz, DMSO-*d*_*6*_) δ = 161.1, 159.3, 143.5 (m), 142.3 (ddt, *J* = 245.9, 14.4, 3.5 Hz), 138.5, 133.3, 129.9, 129.6, 127.7, 122.2, 117.9 (t, *J* = 17.7 Hz), 115.8, 114.97, 54.5 (m), 45.10 ppm. LCMS (ESI): *m/z* 463.0 ([M+H]^+^).

#### 2-(Methylthio)-*N*-{2,3,5,6-tetrafluoro-3’-(^2^H_3_)methoxy-[1,1'-biphenyl]-4-yl}thiophene-3-carboxamide (15a)

(Methylthio)thiophene-3-carboxylic acid (**15b**, 0.50 g, 2.9 mmol) was added to oxalyl chloride (5 mL) at 0°C. The mixture was stirred at rt for 2 h and concentrated under *vacuo* to afford the crude acid chloride intermediate. NaH (530 mg, 60%wt, 13 mmol) was added to a solution of 2,3,5,6-tetrafluoro-3’-(^2^H_3_)methoxy-[1,1'-biphenyl]-4-amine (**12b**, 0.79 g, 2.9 mmol) in anhydrous THF (5 mL) at 0°C. The mixture was stirred at 0°C for 10 min. The acid chloride intermediate was then added, and the mixture was stirred at rt for 6 h, quenched with saturated aq. NH_4_Cl and extracted with EtOAc (3 × 30 mL). The combined organic layer was dried over Na_2_SO_4_, filtered, concentrated and purified by CC (PE:EtOAc = 1:0 to 2:1) to give compound **15a** (0.25 g, yield: 20%) as a colorless solid. LCMS (ESI): *m/z* 431.1 ([M+H]^+^).

#### 2-(*S*-Methylsulfonimidoyl)-*N*-{2,3,5,6-tetrafluoro-3’-(^2^H_3_)methoxy-[1,1'-biphenyl]-4-yl}thiophene-3-carboxamide (16)

(NH_4_)_2_CO_3_ (31 mg, 0.32 mmol) and PhI(OAc)_2_ (0.15 g, 0.47 mmol) were added to a stirred solution of compound **15a** (70 mg, 0.16 mmol) in MeOH (2 mL). The mixture was stirred at rt for 14 h, concentrated and purified by preparative HPLC (gradient A) to afford compound **16** (18 mg, yield: 24%) as a colorless solid. ^1^H-NMR (500 MHz, CD_3_OD) δ = 7.92 (d, *J* = 4.5 Hz, 1H), 7.58 (d, *J* = 5.0 Hz, 1H), 7.43 (t, *J* = 8.0 Hz, 1H), 7.07–7.04 (m, 3H), 3.50 (s, 3H) ppm. ^13^C-NMR (126 MHz, DMSO-*d*_*6*_) δ = 161.2, 159.3, 148.0, 143.4 (m), 142.3 (ddt, *J* = 247.5, 14.9, 3.4 Hz), 137.1, 131.8, 129.9 (d, *J* = 2.6 Hz), 127.6, 122.2, 117.9 (t, *J* = 17.7 Hz), 115.8, 114.9, 54.5 (m), 46.60 ppm. LCMS (ESI): *m/z* 462.3 ([M+H]^+^).

#### 2-(2-Hydroxy-3,4-dioxocyclobut-1-en-1-yl)-*N*-{2,3,5,6-tetrafluoro-3’-(^2^H_3_)methoxy-[1,1'-biphenyl]-4-yl}thiophene-3-carboxamide (17)

1N HCl (0.5 mL) was added to a stirred solution of compound **17a** (0.20 g, 0.34 mmol) in AcOH (3 mL). The mixture was stirred at rt overnight, cooled down to 0°C, diluted with MeCN (3 mL), adjusted to pH=7 with 15% NaOH and purified by preparative HPLC (gradient A) to furnish compound **17** (53 mg, yield: 32%) as a yellow solid. ^1^H-NMR (400 MHz, CD_3_OD) δ = 7.67–7.63 (m, 2H), 7.45–7.40 (m, 1H), 7.06–7.03 (m, 3H) ppm. ^13^C-NMR (126 MHz, DMSO-*d*_*6*_) δ = 210.1, 194.5, 168.8, 160.8, 159.3, 143.4 (m), 142.5 (m), 132.6, 131.2, 130.9, 129.9, 127.8, 126.7, 122.3, 117.3 (m), 115.7, 114.9, 54.5 (m) ppm. LCMS (ESI): *m/z* 479.1 ([M–H]^–^).

#### 2-(1-Hydroxy-2,3-diisopropoxy-4-oxocyclobut-2-en-1-yl)-*N*-{2,3,5,6-tetrafluoro-3’-(^2^H_3_)methoxy-[1,1'-biphenyl]-4-yl}thiophene-3-carboxamide (17a)

To a stirred solution of compound **12a** (0.40 g, 0.86 mmol) in anhydrous THF (4 mL) *n*-BuLi (2.5 M in THF, 0.42 mL) was added at –78°C. The mixture was stirred at –78°C for 30 min. 3,4-diisopropoxycyclo-but-3-ene-1,2-dione (0.19 g, 0.96 mmol) was then added and the mixture stirred at –78°C for another 30 min, quenched with aq. NH_4_HCO_3_ and purified by preparative HPLC (gradient A) to afford compound **17b** (0.20 g, yield: 40%) as a colorless solid. LCMS (ESI): *m/z* 581.3 ([M–H]^–^).

#### 2-(5-Oxo-4,5-dihydro-1,2,4-oxadiazol-3-yl)-*N*-{2,3,5,6-tetrafluoro-3’-(^2^H_3_)methoxy-[1,1'-biphenyl]-4-yl}thiophene-3-carboxamide (18)

CDI (40 mg, 0.25 mmol) and DBU (42 mg, 0.28 mmol) were added to a stirred solution of compound **18a** (0.10 g, 0.23 mmol) in 1,4-dioxane (1 mL). The solution was stirred at 100°C for 3 h, cooled down to rt, concentrated and purified by preparative HPLC (gradient B) to afford compound **18** (38 mg, yield: 36%) as a colorless solid. ^1^H-NMR (400 MHz, DMSO-*d*_6_) δ = 12.79 (br s, 1H), 11.01 (br s, 1H), 8.09 (d, *J* = 4.8 Hz, 1H), 7.50–7.56 (m, 2H), 7.14–7.09 (m, 3H) ppm. ^13^C-NMR (126 MHz, DMSO-*d*_6_) δ = 159.3, 159.2, 143. (m), 142.3 (m), 136.7, 131.5, 129.9, 128.7, 127.6, 122.2, 118.1 (t, *J* = 17.6 Hz), 115.8, 115.0, 54.5 (m) ppm. LCMS (ESI): *m/z* 469.0 ([M+H]^+^).

#### 2-(*N*’-Hydroxycarbamimidoyl)-*N*-{2,3,5,6-tetrafluoro-3’-(^2^H_3_)methoxy-[1,1'-biphenyl]-4-yl}thiophene-3-carboxamide (18a)

Hydroxylamine hydrochloride (51 mg, 0.73 mmol) and Hünig's base (95 mg, 0.74 mmol) were added to a solution of compound **18b** (0.20 g, 0.49) in MeOH (2 mL). The solution was stirred at 70°C for 4 h, cooled down to rt, concentrated and purified by preparative HPLC (gradient B) to give compound **18a** (0.10 g, yield: 46%) as a colorless solid. LCMS (ESI): *m/z* 443.2 ([M+H]^+^).

#### 2-Cyano-*N*-{2,3,5,6-tetrafluoro-3’-(^2^H_3_)methoxy-[1,1'-biphenyl]-4-yl}thiophene-3-carboxamide (18b)

Cyanuric chloride (0.35 g, 1.9 mmol) was added to a stirred solution of compound **18c** (0.40 g, 0.94 mmol) in DMF (5 mL). The solution was stirred at 0°C for 3 h, concentrated and purified by preparative HPLC (gradient B) to give compound **18b** (0.30 g, yield: 78%) as a colorless solid. LCMS (ESI): *m/z* 410.0 ([M+H]^+^).

#### *N*^3^-{2,3,5,6-Tetrafluoro-3’-(^2^H_3_)methoxy-[1,1'-biphenyl]-4-yl}thiophene-2,3-dicarboxamide (18c)

To a stirred solution of compound **12** (0.50 g, 1.2 mmol) in CH_2_Cl_2_ (5 mL) NH_4_Cl (0.19 g, 3.6 mmol), EDCI (0.45 g, 2.9 mmol) and DMAP (0.13 g, 1.1 mmol) were added at rt. The solution was stirred at 60°C for 3 h, cooled down to rt, diluted with water and extracted with EtOAc (3 × 20 mL). The organic layer was washed with brine (50 mL), dried over Na_2_SO_4_, filtered, concentrated and purified by preparative HPLC (gradient B) to give compound **18c** (0.40 g, yield: 80%) as a colorless solid. LCMS (ESI): *m/z* 428.1 ([M+H]^+^).

#### *N*-{2,3,5,6-Tetrafluoro-3’-(^2^H_3_)methoxy-[1,1'-biphenyl]-4-yl}-2-(2*H*-tetrazol-5-yl)thiophene-3-carboxamide (19)

NaN_3_ (48 mg, 0.73 mmol) and NH_4_Cl (27 mg, 0.50 mmol) were added to a stirred solution of compound **18b** (0.10 g, 0.24 mmol) in DMF (1 mL). The solution was stirred at 125°C for 15 h, cooled down to rt, diluted with water and extracted with EtOAc (3 × 10 mL). The combined organic layer was dried over Na_2_SO_4_, filtered, concentrated and purified by preparative HPLC (gradient A) to obtain compound **19** (51 mg, yield: 46%) as a colorless solid. ^1^H-NMR (500 MHz, CD_3_OD) δ = 7.99 (d, *J* = 5.5 Hz, 1H), 7.80 (d, *J* = 5.0 Hz, 1H), 7.44 (dd, *J* = 7.0, 9.0 Hz, 1H), 7.08–7.05 (m, 3H) ppm. ^13^C NMR (126 MHz, DMSO-*d*_6_) δ = 159.4, 159.3, 158.2, 157.9, 152.8, 143.5 (m), 142.3 (m), 136.1, 132.2, 129.9, 129.8, 127.7, 126.3, 122.3, 117.8 (t, *J* = 17.7 Hz), 116.1 (m), 115.8, 114.9, 54.5 (m) ppm.LCMS (ESI): m/z 453.2 ([M+H]^+^).

#### 4-(5-Oxo-4,5-dihydro-1,2,4-oxadiazol-3-yl)-*N*-{2,3,5,6-tetrafluoro-3’-(^2^H_3_)methoxy-[1,1'-biphenyl]-4-yl}thiophene-3-carboxamide (21)

CDI (109 mg, 0.67 mmol) was added to a stirred solution of compound **21a** (0.20 g, 0.45 mmol) in DMF (3 mL). The mixture was stirred at 100°C under a nitrogen atmosphere for 15 h, cooled down to rt, diluted with water (50 mL) and extracted with EtOAc (3 × 40 mL). The combined organic layer was washed with brine (3 × 10 mL), dried over Na_2_SO_4_, filtered, concentrated and purified by preparative HPLC (gradient C) to afford compound **21** (45 mg, yield: 21%) as a colorless powder. ^1^H-NMR (500 MHz, DMSO-*d*_6_) δ = 12.57 (s, 1H), 8.53 (d, *J* = 3.1 Hz, 1H), 8.21 (d, *J* = 3.1 Hz, 1H), 7.47 (t, *J* = 7.9 Hz, 1H), 7.15–7.08 (m, 3H) ppm. ^13^C-NMR (126 MHz, DMSO-*d*_6_) δ = 160.38, 159.31, 155.3 (m), 143.4 (m), 142.3 (m), 133.7, 133.5, 132.2, 129.9, 127.7, 124.0, 122.2, 117.7 (t, *J* = 17.8 Hz), 116.1 (m), 115.8, 114.9, 54.5 (m) ppm. LCMS (ESI): *m/z* 469.0 ([M+H]^+^).

#### (*E*)-4-(*N*’-Hydroxycarbamimidoyl)-*N*-{2,3,5,6-tetrafluoro-3’-(^2^H_3_)methoxy-[1,1'-biphenyl]-4-yl}thiophene-3-carboxamide (21a)

To a stirred solution of compound **21b** (4.0 g, 9.8 mmol) in EtOH (20 mL) a 50% aqueous solution of hydroxylamine (1.3 mL, 20 mmol) was added at 0°C. The mixture was stirred at 50°C for 5 h, cooled down to rt and diluted with water (100 mL). The solid was collected by filtration, washed with water (3 × 50 mL) and dried *in vacuo* to give the compound **21a** (3.8 g, yield: 88%) as an off-white solid. LCMS (ESI): *m/z* 443.0 [M+H]^+^.

#### 4-Cyano-*N*-{2,3,5,6-tetrafluoro-3’-(^2^H_3_)methoxy-[1,1'-biphenyl]-4-yl}thiophene-3-carboxamide (21b)

To a stirred suspension of compound **21c** (5.0 g, 12 mmol) in anhydrous THF (30 mL) Burgess reagent (5.6 g, 23 mmol) was added under argon atmosphere at 0–5°C. The mixture was stirred for 1 h at rt, diluted with water (200 mL), separated by filtration, washed with water (3 × 50 mL) and dried *in vacuo* to obtain compound **21b** (4.5 g, yield: 94%) as an off-white solid. LCMS (ESI): m/z 409.9 ([M+H]^+^).

#### *N*-{2,3,5,6-Tetrafluoro-3’-(^2^H_3_)methoxy-[1,1'-biphenyl]-4-yl}thiophene-3,4-dicarboxamide (21c)

EDCI (8.9 g, 57 mmol) was added to a stirred solution of compound **20** (10 g, 23 mmol), DMAP (2.8 g, 23 mmol) and NH_4_Cl (12 g, 220 mmol) in DMF (100 mL). The mixture was stirred at 60°C overnight and then allowed to reach rt, diluted with water (150 mL) and extracted with EtOAc (3 × 100 mL). The combined organic layer was dried over Na_2_SO_4_, filtered, concentrated and purified by CC (DCM:MeOH = 1:0 to 12:1) to obtain the crude product as an off-white solid, which was resuspended in MeCN (200 mL) and stirred at rt for 2 h, filtered and dried *in vacuo* at 60°C to afford compound **21c** (5.0 g, yield: 50%) as a colorless solid. LCMS (ESI): m/z 428.0 ([M+H]^+^).

#### *N*-{2,3,5,6-Tetrafluoro-3’-(^2^H_3_)methoxy-[1,1'-biphenyl]-4-yl}-4-(2*H*-tetrazol-5-yl)thiophene-3-carboxamide (22)

NaN_3_ (56 mg, 0.86 mmol) and NH_4_Cl (47 mg, 0.88 mmol) were added to a stirred solution of compound **21b** (0.30 g, 0.73 mmol) in DMF (5 mL). The solution was stirred in a sealed tube at 120°C for 15 h, cooled down to rt, diluted with water and extracted with EtOAc (3 × 40 mL). The combined organic layer was dried over Na_2_SO_4_, filtered, concentrated and purified by preparative HPLC (gradient C) to afford compound **22** (82 mg, yield: 25%) as a colorless powder. ^1^H-NMR (300 MHz, DMSO-*d*_6_) δ = 10.96 (s, 1H), 8.54 (d, *J* = 3.1 Hz, 1H), 8.22 (d, *J* = 3.1 Hz, 1H), 7.47 (t, *J* = 7.8 Hz, 1H), 7.15–7.04 (m, 3H) ppm. ^13^C-NMR (126 MHz, DMSO-*d*_6_) δ = 160.9, 159.3, 151.6, 142.3 (m), 143.4 (m), 133.8, 133.5, 130.9, 129.9, 127.7, 125.1, 122.2, 117.6 (t, *J* = 17.7 Hz), 116.3 (m), 115.8, 114.9, 54.5 (m) ppm. LCMS (ESI): *m/z* = 453.0 ([M+H]^+^).

#### *N*-{2,3,5,6-Tetrafluoro-3’-(^2^H_3_)methoxy-[1,1'-biphenyl]-4-yl}-4-(5-thioxo-4,5-dihydro-1,2,4-oxadiazol-3-yl)thiophene-carboxamide (23)

1,1'-thiocarbonyldiimidazole (0.10 g, 0.56 mmol) and DIPEA (0.1 mL) were added to a stirred solution of compound **21a** (0.20 g, 0.45 mmol) in DMF (3 mL). The suspension was stirred at 100°C under a nitrogen atmosphere for 15 h, cooled down to rt, diluted with water and extracted with EtOAc (3 × 40 mL). The combined organic layer was washed with brine, dried over Na_2_SO_4_, filtered, concentrated and purified by preparative HPLC (gradient C) to furnish compound **23** as a colorless powder (73 mg, 33%). ^1^H-NMR (300 MHz, DMSO-*d*_6_) δ = 10.90 (s, 1H), 8.58 (d, *J* = 3.1 Hz, 1H), 8.30 (d, *J* = 3.1 Hz, 1H), 7.47 (t, *J* = 7.9 Hz, 1H), 7.18–7.04 (m, 3H) ppm. ^13^C-NMR (126 MHz, DMSO-*d*_6_) δ = 160.3, 159.3, 143.4 (m), 142.3 (m), 133.8, 133.7, 133.3, 129.9, 127.7, 122.2, 117.8 (t, *J* = 17.8 Hz), 116.0 (m), 115.8, 114.9, 54.5 (m) ppm. LCMS (ESI): *m/z* 484.9 ([M+H]^+^).

#### 4-(2-Methyl-5-oxo-2,5-dihydro-1,2,4-oxadiazol-3-yl)-*N*-{2,3,5,6-tetrafluoro-3’-(^2^H_3_)methoxy-[1,1'-biphenyl]-4-yl}thiophene-3-carboxamide (24)

1,1'-carbonyldiimidazole (0.11 g, 6.8 mmol) and Hünig's base (0.1 mL) were added to a stirred solution of compound **21b** (0.20 g, 4.4 mmol) in DMF (5 mL). The suspension was stirred at 80°C under nitrogen for 15 h, cooled down to rt, diluted with water (50 mL) and 0.6N HCl (1 mL) and extracted with EtOAc (3 × 30 mL). The combined organic layer was washed with brine, dried over Na_2_SO_4_, filtered, concentrated and purified by preparative HPLC (gradient C) to afford compound **24** (0.13 g, yield: 62%) as an off-white powder. ^1^H-NMR (500 MHz, DMSO-*d*_6_) δ = 10.89 (s, 1H), 8.62 (d, *J* = 3.1 Hz, 1H), 8.31 (d, *J* = 3.0 Hz, 1H), 7.47 (t, *J* = 7.9 Hz, 1H), 7.15–7.08 (m, 3H), 3.57 (s, 3H) ppm. ^13^C-NMR (126 MHz, DMSO-*d*_6_) δ = 163.6, 162.8, 160.4, 159.3, 142.4 (m), 143.4 (m), 134.0, 133.3, 133.2, 129.9, 127.6, 124.4, 122.2, 118.0 (t, *J* = 17.9 Hz), 115.9 (m), 115.7, 115.0, 54.5 (m), 36.0 ppm. LCMS (ESI): *m/z* 483.1 ([M+H]^+^).

#### 4-(*N*-Hydroxy-*N*-methylcarbamimidoyl)-*N*-{2,3,5,6-tetrafluoro-3’-(^2^H_3_)methoxy-[1,1'-biphenyl]-4-yl}thiophene-3-carboxamide (24a)

*N*-Methylhydroxylamine hydrochloride (1.3 g, 16 mmol) and Hünig's base (2.6 mL) were added to a solution of compound **21b** (2.0 g, 4.9 mmol) in EtOH (15 mL). The suspension was stirred at 80°C for 5 h, cooled down to rt, concentrated, diluted with water (100 mL) and extracted with EtOAc (3 × 20 mL). The combined organic layer was washed with brine, dried over Na_2_SO_4_, filtered, concentrated and purified by CC (DCM:MeOH = 99:1 to 11:1) to afford compound **24a** (1.2 g, 54%) as a colorless powder. LCMS (ESI): *m/z* 457.0 ([M+H]^+^).

#### 4-(2-Methyl-5-thioxo-2,5-dihydro-1,2,4-oxadiazol-3-yl)-*N*-{2,3,5,6-tetrafluoro-3’-(^2^H_3_)methoxy-[1,1'-biphenyl]-4-yl}thiophene-3-carboxamide (25)

1,1′-thiocarbonyldiimidazol (0.12 g, 6.7 mmol) and Hünig's base (0.1 mL) were added to a stirred solution of compound **21b** (0.20 g, 4.4 mmol) in DMF (5 mL). The suspension was stirred at 80°C under a nitrogen atmosphere for 15 h, cooled down to rt, diluted with water (50 mL) and 0.6N HCl (1 mL) and extracted with EtOAc (3 × 30 mL). The combined organic layer was washed with brine, dried over Na_2_SO_4_, filtered, concentrated and purified by preparative HPLC (gradient C) to afford compound **25** (0.13 g, yield: 60%) as an off-white powder. ^1^H-NMR (500 MHz, DMSO-*d*_6_) δ = 10.93 (s, 1H), 8.68 (d, *J* = 3.0 Hz, 1H), 8.38 (d, *J* = 3.0 Hz, 1H), 7.47 (t, *J* = 8.0 Hz, 1H), 7.14–7.08 (m, 3H), 3.77 (s, 3H) ppm. ^13^C-NMR (126 MHz, DMSO-*d*_6_) δ = 192.0, 160.7, 159.8, 143.9, 142.8, 134.9, 134.3, 134.1, 130.4, 128.1, 123.2, 122.7, 118.5 (m), 116.2, 115.5, 54.9 (m), 37.1 ppm. LCMS (ESI): *m/z* 499.1 ([M+H]^+^).

#### 4-(5-Oxo-4,5-dihydro-1,3,4-oxadiazol-2-yl)-*N*-{2,3,5,6-tetrafluoro-3’-(^2^H_3_)methoxy-[1,1'-biphenyl]-4-yl}thiophene-3-carboxamide (26)

1,1'-carbonyldiimidazole (0.11 mg, 6.8 mmol) was added to a solution of compound **26a** (0.20 g, 0.45 mmol) in DMF (3 mL). The mixture was stirred at rt under a nitrogen atmosphere for 4 h and was then diluted with water (50 mL). The precipitate formed was filtered, washed with water (3 × 25 mL) and dried *in vacuo* to yield compound **26** (0.11 g, 35%) as an off-white solid. ^1^H-NMR (300 MHz, DMSO-*d*_6_) δ = 12.52 (s, 1H), 10.79 (s, 1H), 8.35 (d, *J* = 3.1 Hz, 1H), 8.25 (d, *J* = 3.1 Hz, 1H), 7.47 (t, *J* = 7.9 Hz, 1H), 7.17-7.05 (m, 3H) ppm. ^13^C-NMR (126 MHz, DMSO-*d*_6_) δ = 161.6, 159.8, 154.9, 150.9, 143.9 (m), 142.8 (m), 134.5, 132.8, 131.9, 130.4, 128.2, 124.4, 122.7, 118.3 (t, *J* = 17.7 Hz), 116.5 (m), 116.2, 115.4, 55.1 (m) ppm. LCMS (ESI): m/z = 469.0 (M+H)^+^.

#### 4-(Hydrazinecarbonyl)-*N*-{2,3,5,6-tetrafluoro-3’-(^2^H_3_)methoxy-[1,1'-biphenyl]-4-yl}thiophene-3-carboxamide (26a)

Oxalyl chloride (0.4 mL) was added to a stirred mixture of compound **20** (1.0 g, 2.3 mmol) and DMF (one drop) in anhydrous DCM (10 mL) at 0–5°C. The mixture was stirred for 30 min at rt and then for 30 min at 50°C, concentrated *in vacuo* and co-evaporated with DCM (3 × 5 mL). The solid was diluted in THF (15 mL) and Hünig's base (0.1 mL) and hydrazine hydrate (3 mL, 80%) were added at 5°C. The mixture was stirred at rt for 15 h and diluted with water. The resulting solid was collected by filtration, washed with water and dried *in vacuo* to yield compound **26a** (0.51 g, yield: 49%) as a colorless solid. LCMS (ESI): *m/z* 443.0 ([M+H]^+^).

#### *N*-(2,3,5,6-Tetrafluoro-3’-(^2^H_3_)methoxy-[1,1'-biphenyl]-4-yl)-4-{5-thioxo-4,5-dihydro-1,3,4-oxadiazol-2-yl}thiophene-3-carboxamide (27)

1,1'-thiocarbonyldiimidazole (0.12 g, 0.67 mmol) and DBU (75 mg, 0.49 mmol) were added to a stirred solution of compound **26a** (0.20 g, 0.45 mmol) in DMF (3 mL). The suspension was stirred at 100°C under a nitrogen atmosphere for 15 h, cooled down to rt, diluted with water (50 mL) and 0.2M HCl (10 mL) and extracted with EtOAc (3 × 40 mL). The combined organic layer was washed with brine, dried over Na_2_SO_4_, filtered, concentrated and purified by preparative HPLC (gradient C) to afford compound **27** (58 mg, yield: 26%) as a white powder. ^1^H-NMR (500 MHz, DMSO-*d*_6_) δ = 14.67 (s, 1H), 10.84 (s, 1H), 8.43 (d, *J* = 3.1 Hz, 1H), 8.38 (d, *J* = 3.1 Hz, 1H), 7.47 (t, *J* = 8.0 Hz, 1H), 7.15–7.08 (m, 3H) ppm. ^13^C-NMR (126 MHz, DMSO-*d*_6_) δ = 178.0, 161.3, 159.8, 157.4, 143.9 (m), 142.8 (m), 134.5, 133.5, 130.4, 128.1, 122.7, 122.7, 118.3 (t, *J* = 17.8 Hz), 116.4 (m), 116.2, 115.5, 55.0 (m) ppm. LCMS (ESI): *m/z* 485.0 ([M+H]^+^).

#### 4-(2-Oxido-3*H*-1,2,3,5-oxathiadiazol-4-yl)-*N*-{2,3,5,6-tetrafluoro-3’-(^2^H_3_)methoxy-[1,1'-biphenyl]-4-yl}thiophene-3-carboxamide (28)

Under a nitrogen atmosphere a solution of thionyl chloride (50 µL) in anhydrous DCM (0.5 mL) was added dropwise to a solution of compound **21a** (0.25 g, 0.57 mmol) and pyridine (90 µL) in anhydrous DCM (5 mL) at −50°C. After being stirred for 1 h at −50°C, the mixture was allowed to warm to rt and stirred for 2 h, quenched with water (10 mL) and 0.2 N HCl (1 mL) and extracted with DCM (3 × 10 mL). The combined organic layer was dried over Na_2_SO_4_, filtered, concentrated and purified by preparative HPLC (gradient C) to afford compound **28** as a colorless powder (80 mg, 29%). ^1^H-NMR (300 MHz, DMSO-*d*_6_) δ = 11.78 (s, 1H), 10.85 (s, 1H), 8.48 (d, *J* = 3.1 Hz, 1H), 8.24 (d, *J* = 3.1 Hz, 1H), 7.47 (t, *J* = 7.9 Hz, 1H), 7.18–7.04 (m, 3H) ppm. ^13^C-NMR (126 MHz, DMSO-*d*_6_) δ = 160.9, 159.3, 143.4 (m), 142.3 (m), 134.3, 133.1, 132.2, 129.9, 127.7, 122.2, 117.7 (m), 116.3 (m), 115.8, 114.9, 54.6 (m) ppm. LCMS (ESI): m/z 488.9 ([M+H]^+^).

#### *N*-{2,3,5,6-Tetrafluoro-3’-(^2^H_3_)methoxy-[1,1'-biphenyl]-4-yl}-4-(2*H*-tetrazol-5-yl)-2,5-dihydrofuran-3-carboxamide(30)

NaN_3_ (0.18 g, 2.8 mmol) and NH_4_Cl (30 mg, 0.56 mmol) were added to a stirred solution of compound **30b** (0.10 g, 0.25 mmol) in DMF (4 mL). The mixture was stirred in a sealed tube at 125°C for 15 h, cooled down to rt, diluted with water and extracted with EtOAc (3 × 10 mL). The combined organic layer was dried over Na_2_SO_4_, filtered, concentrated and purified by preparative HPLC (gradient A) to afford compound **30** (40 mg, yield: 36%) as a colorless powder. ^1^H-NMR (400 MHz, CD_3_OD) δ = 7.45–7.41 (m, 1H), 7.07–7.03 (m, 3H), 5.43–5.40 (m, 2H), 5.15–5.12 (m, 2H) ppm. ^13^C-NMR (126 MHz, DMSO-*d*_6_) δ 160.9, 159.8, 154.0, 144.0 (m), 142.6 (m), 132.0, 130.4, 128.3, 127.1, 122.7, 117.8 (t, *J* = 17.6 Hz), 116.9 (m), 116.2, 115.4, 78.6, 77.8, 54.9 (m) ppm. LCMS (ESI): *m/z* 438.8 ([M+H]^+^).

#### 4-Cyano-*N*-{2,3,5,6-tetrafluoro-3’-(^2^H_3_)methoxy-[1,1'-biphenyl]-4-yl}-2,5-dihydrofuran-3-carboxamide (30b)

To a stirred solution of compound **30c** (0.15 mg, 0.36 mmol) in DMF (5 mL) cyanuric chloride (0.14 g, 0.76 mmol) was added at 0°C. The mixture was stirred at rt for 3 h, diluted with water (15 mL) and extracted with EtOAc (3 × 10 mL). The combined organic layer was dried over Na_2_SO_4_, filtered, concentrated and purified by CC (PE:EtOAc = 4:1) to afford compound **30b** (0.10 g, yield: 70%) as a colorless solid. LCMS (ESI): *m/z* 396.0 ([M+H]^+^).

#### *N*-{2,3,5,6-Tetrafluoro-3’-(^2^H_3_)methoxy-[1,1'-biphenyl]-4-yl}-2,5-dihydrofuran-3,4-dicarboxamide (30c)

To a stirred solution of compound **29** (0.30 g, 0.73 mmol) in DMF (10 mL) NH_4_Cl (0.12 g, 2.2 mmol), EDCI (0.14 mg, 0.90 mmol) and DMAP (97 mg, 0.79 mmol) were added. The mixture was stirred at 60°C for 15 h and then allowed to reach rt, diluted with water (150 mL) and extracted with EtOAc (3 × 20 mL). The combined organic layer was dried over Na_2_SO_4_, filtered, concentrated and purified by CC (PE: EtOAc = 5:1) to give compound **30c** (0.15 g, yield: 52%) as a yellow solid. LCMS (ESI): m/z 414.0 ([M+H]^+^).

#### *N*-{2,3,5,6-Tetrafluoro-3’-(^2^H_3_)methoxy-[1,1'-biphenyl]-4-yl}-2-(2*H*-tetrazol-5-yl)benzamide (31)

NEt_3_•HCl (76 mg, 0.55 mmol) and NaN_3_ (36 mg, 0.55 mmol) were added to a solution of compound **31a** (0.15 g, 0.37 mmol) in anhydrous DMF (2 mL). The mixture was stirred under microwave irradiation at 120°C for 4 h, cooled down to rt, concentrated and purified by preparative HPLC (gradient A) to afford compound **29** (13 mg, yield: 8%) as a colorless solid. ^1^H-NMR (400 MHz, CD_3_OD) δ = 7.85 (d, *J* = 7.2 Hz, 2H), 7.72–7.64 (m, 2H), 7.42 (t, *J* = 8.0 Hz, 1H), 7.06–7.02 (m, 3H) ppm. ^13^C-NMR (126 MHz, DMSO) δ = 166.2, 159.3, 156.6, 143.3 (m), 142.3 (m), 134.5, 130.9, 130.0, 129.9, 129.8, 129.2, 127.7, 125.5, 122.2, 117.5, 116.5, 115.7, 114.9, 54.5 (m) ppm. LCMS (ESI): *m/z* 447.3 ([M+H]^+^).

#### 2-Cyano-*N*-{2,3,5,6-tetrafluoro-3’-(^2^H_3_)methoxy-[1,1'-biphenyl]-4-yl}benzamide (31a)

To a stirred solution of 2-cyanobenzoic acid (0.40 g, 2.7 mmol) in anhydrous CH_2_Cl_2_ (5 mL) SOCl_2_ (0.65 g, 5.5 mmol) was added at 0°C. The mixture was stirred at 0°C for 2 h and concentrated under *vacuo* to afford the crude acid chloride intermediate. To a stirred solution of 2,3,5,6-tetrafluoro-3’-(^2^H_3_)-methoxy-[1,1'-biphenyl]-4-amine (**12b**, 0.75 g, 2.7 mmol) in anhydrous THF (5 mL) NaH (0.55 g, 60%wt) was added at 0°C and the mixture was stirred at 0°C for 30 min. Then the crude acid chloride intermediate was added at 0°C and the mixture was stirred at 0°C for 2 h, quenched with saturated aq. NH_4_Cl and extracted with EtOAc (3 × 20 mL). The combined organic layer was dried over Na_2_SO_4_, filtered and concentrated under reduced pressure to afford compound **31a** (0.20 g, yield: 18%) as a yellow solid. LCMS (ESI): *m/z* 404.2 ([M+H]^+^).

#### *N*-{3-Fluoro-3’-methoxy-[1,1'-biphenyl]-4-yl}-2-(2*H*-tetrazol-5-yl)cyclopent-1-ene-1-carboxamide (32)

NaN_3_ (46 mg, 0.71 mmol) and NH_4_Cl (38 mg, 0.71 mmol) were added to a stirred solution of compound **32a** (0.20 g, 0.59 mmol) in DMF (3 mL). The mixture was then stirred for 15 h in a sealed tube at 120°C, cooled down to rt, diluted with water and extracted with EtOAc (3 × 10 mL). The organic layer was washed with brine, dried over Na_2_SO_4_, filtered, concentrated and purified by preparative HPLC (gradient C) to afford compound **30** (20 mg, yield 9%) as an off-white powder. ^1^H-NMR (500 MHz, DMSO-*d*_6_) δ = 10.93 (s, 1H), 8.05 (t, *J* = 8.3 Hz, 1H), 7.64 (dd, *J* = 12.3, 2.1 Hz, 1H), 7.55 (d, *J* = 8.5 Hz, 1H), 7.38 (t, *J* = 7.9 Hz, 1H), 7.30–7.21 (m, 2H), 6.95 (dd, *J* = 8.2, 2.5 Hz, 1H), 3.83 (s, 3H) ppm. ^13^C-NMR (126 MHz, DMSO-*d*_6_) δ = 164.3, 160.3, 154.8 (d, *J* = 246.2 Hz), 140.9, 140.4 (d, *J* = 1.9 Hz), 138.1 (d, *J* = 7.2 Hz), 130.5, 128.6, 125.7 (d, *J* = 12.2 Hz), 125.4, 122.9 (d, *J* = 2.9 Hz), 119.3, 114.1 (d, *J* = 20.5 Hz), 113.9, 112.5, 55.7, 36.7, 36.6, 21.9 ppm. LCMS (ESI): *m/z* 380.1 (M+H]^+^).

#### 2-Cyano-*N*-(3-fluoro-3’-methoxy-[1,1'-biphenyl]-4-yl)cyclopent-1-ene-1-carboxamide (32a)

To a stirred suspension of compound **32b** (7.0 g, 2.0 mmol) in anhydrous THF (125 mL) Burgess reagent (9.4 g, 3.9 mmol) was added under an argon atmosphere at 0–5°C. The mixture was stirred for 1 h at rt and diluted with water (300 mL). The precipitate formed was separated by filtration, washed with water (3 × 100 mL) and dried *in vacuo* to obtain the crude product, which was recrystallized from EtOAc at 50°C to give compound **32a** (6.3 g, yield: 87%) as a colorless solid. ^1^H-NMR (80 MHz, DMSO-*d*_6_) δ = 9.79 (s, 1H), 7.61–6.88 (m, 7H), 3.72 (s, 3H), 2.67 (t, *J* = 6.7 Hz, 4H), 1.92 (quint, *J* = 7.3 Hz, 2H) ppm. LCMS (ESI): *m/z* 337.0 ([M+H]^+^).

#### *N*-(3-Fluoro-3’-methoxy-[1,1'-biphenyl]-4-yl)cyclopent-1-ene-1,2-dicarboxamide (32b)

To a stirred mixture of vidofludimus (**7**, 20 g, 56 mmol) and DMF (2 drops) in anhydrous DCM (150 mL) oxalyl chloride (8.0 mL) was added at 0–5°C within 1 min. The mixture was stirred at rt for 30 min, then heated to 50°C, stirred for another 30 min and concentrated *in vacuo*. The remaining residue was dissolved in DCM (50 mL) and concentrated again under reduced pressure. The resulting solid was treated with PE (20 mL), filtered and washed with PE. The solid was dried *in vacuo* to afford crude acid chloride (21 g), concentrated and resuspended with water (200 mL). The precipitate formed was filtered and washed with water (3 × 50 mL) and dried *in vacuo* to afford compound **32b** (7.7 g; yield 81%) as an off-white powder. LCMS (ESI): *m/z* 355.0 ([M+H]^+^).

### In vitro Characterization

#### Hybrid reporter gene assays

NR modulation was determined in Gal4 hybrid reporter gene assays in HEK293T cells (German Collection of Microorganisms and Cell Culture GmbH, DSMZ) using pFR-Luc (Stratagene, La Jolla, CA, USA; reporter), pRL-SV40 (Promega, Madison, WI, USA; internal control) and pFA-CMV-hNR-LBD^47,48^ plasmids coding for the hinge region and ligand binding domain of the canonical isoform of the respective NR. Cells were cultured in Dulbecco’s modified Eagle’s medium (DMEM), high glucose supplemented with 10% fetal calf serum (FCS), sodium pyruvate (1 mM), penicillin (100 U/mL), and streptomycin (100 μg/mL) at 37°C and 5% CO_2_ and seeded in 96-well plates (3×10^4^ cells/well). After 24 h, medium was changed to Opti-MEM without supplements and cells were transiently transfected using Lipofectamine LTX reagent (Invitrogen, Carlsbad, CA, USA) according to the manufacturer’s protocol. Five hours after transfection, cells were incubated with the test compounds in Opti-MEM supplemented with penicillin (100 U/mL), streptomycin (100 μg/mL) and 0.1% DMSO for 16 h before luciferase activity was measured using the Dual-Glo Luciferase Assay System (Promega) according to the manufacturer’s protocol on a Tecan Spark luminometer (Tecan Deutschland GmbH, Crailsheim, Germany). Firefly luminescence was divided by Renilla luminescence and multiplied by 1000 resulting in relative light units (RLU) to normalize for transfection efficiency and cell growth. Fold activation was obtained by dividing the mean RLU of test compound by the mean RLU of the untreated control. All samples were tested in at least three biologically independent experiments in duplicates. For dose-response curve fitting and calculation of EC_50_ values, the equation “[Agonist] vs. response -- Variable slope (four parameters)” was used in GraphPad Prism (version 7.00, GraphPad Software, La Jolla, CA, USA).

#### DHODH inhibition assay

Inhibition of DHODH was measured in vitro using an *N-*terminally truncated recombinant DHODH enzyme as described previously^20^. The final assay mixture contained 60 *μ*M 2,6-dichloroindophenol, 50 *μ*M decylubiquinone, 100 *μ*M dihydroorotate, and the DHODH protein whose concentration was adjusted in a way that an average slope of approx. 0.2 AU/min served as the positive control (no inhibitor). Measurements were performed in 50 mM TrisHCl, 150 mM KCl, and 0.1% Triton X-100 at pH 8.0 and at 30°C with at least six different concentrations of a test compound. The reaction was started by adding dihydroorotate and measuring the absorption at 600 nm for 2 min. Each test compound concentration used for IC_50_ calculation was tested in at least three independent experiments.

#### Evaluation of Nurr1-Regulated Gene Expression

N27 rat dopaminergic neural cells (SCC048, Sigma-Aldrich, Darmstadt, Germany) were cultured in RPMI 1640 medium (Gibco, Thermo Fisher Scientific, Waltham, MA, USA) supplemented with 10% FCS, penicillin (100 U/mL), and streptomycin (100 μg/mL) at 37°C and 5% and seeded in 12-well plates (3×10^5^ cells/well). After 8 h, the medium was changed to RPMI 1640 medium (Gibco, Thermo Fisher Scientific) supplemented with 0.2% FCS, penicillin (100 U/mL), and streptomycin (100 μg/mL), and the cells were incubated for another 22 h, before the medium was changed again to RPMI 1640 medium (Gibco, Thermo Fisher Scientific) supplemented with 0.2% FCS, penicillin (100 U/mL), and streptomycin (100 μg/mL), additionally containing either **32** (0.3, 1 or 3 μM) in 0.1% DMSO or 0.1% DMSO alone. After 21 h of incubation, the medium was removed, cells were washed with phosphate-buffered saline (PBS), and after full aspiration of residual liquids immediately frozen at –80°C until further procession. Total RNA was isolated using peqGOLD Total RNA Kit (VWR International, Darmstadt, Germany) following the manufacturer’s instructions. RNA concentration and purity were assessed using a NanoDrop One UV-vis spectrophotometer (Thermo Fisher Scientific) at 260/280 nm. Right before reverse transcription (RT), RNA was linearized at a concentration of 133 ng/μL at 65°C for 10 min and then immediately incubated on ice for at least 1 min. Reverse transcription was performed using 2 μg of total RNA, 20 U Recombinant RNasin Ribonuclease Inhibitor (Promega, Mannheim, Germany), 100 U SuperScript IV Reverse Transcriptase including 5× First Strand Buffer and 0.1 M dithiothreitol (Thermo Fisher Scientific), 3.75 ng of linear acrylamide, 625 ng of random hexamer primers (Merck, Darmstadt, Germany), and 11.25 nmol of deoxynucleoside triphosphate mix (2.8 nmol each ATP, TTP, CTP, GTP; Thermo Fisher Scientific) at a volume of 22.45 μL at 50°C for 10 min and 80°C for 10 min using a Thermal cycler XT96 (VWR International). A quantitative polymerase chain reaction (qPCR) was conducted using a qTOWERiris (Analytik Jena, Jena, Germany) and a SYBR green-based detection method. 0.2 μL of prepared cDNA was added to 6 pmol each of forward and reverse primer, 0.8 U Taq DNA Polymerase (New England Biolabs, Ipswich, MA, USA), 40 ppm SYBR Green I (Sigma-Aldrich), 15 nmol of deoxynucleoside triphosphate mix (as indicated above), 60 nmol of MgCl_2_, 4 μg of bovine serum albumin (Thermo Fisher Scientific), 20% BioStab PCR Optimizer II (Merck, Darmstadt, Germany), and 10% Taq buffer without detergents (Thermo Fisher Scientific), topped up to a final volume of 20 μL with ddH_2_O. Samples underwent 40 cycles of 15 s denaturation at 95°C, 15 s of primer annealing at primer-specific temperatures and 20 s of elongation at 68°C. PCR product specificity was evaluated using a melting curve analysis ranging from 65 to 95°C. Nurr1 target gene expression was normalized to rGAPDH mRNA expression per sample using the ΔCt-method. The following primers and annealing temperatures were used: rGAPDH (59.4°C): 5’-CAG CCG CAT CTT CTT GTG C-3’ (fwd), 5’-AAC TTG CCG TGG GTA GAG TC-3’ (rev); rTH (59.4°C): 5’-TGG GGA GCT GAA GGC TTA TG-3’ (fwd), 5’-AGA GAA TGG GCG CTG GAT AC-3’ (rev); rFLRT2 (59.0°C): 5’-AAG GAG ACA AGG CTA CCA GAT TAC-3’ (fwd), 5’-GCA AAG CGT GAT GCC AAG TA-3’ (rev); rSESN3 (62.4°C): 5’-TCG GCC AAC TAC CTG CTC TG-3’ (fwd), 5’-CGT GTT TGC TTG GAC AAC TTC CT-3’ (rev); rCRMP4 (58.0°C): 5’-TGT CCT ACC AGG GCA AGA A-3’ (fwd), 5’-ATC AGA TTG TCT CCA ATT TGC TTT A-3’ (rev); rBDNF (58.0°C): 5’-AGT CTA GAA CCT TGG GGA CC-3’ (fwd), 5’-GCC TTC ATG CAA CCG AAG TA-3’ (rev); rBIRC5 (59.4°C): 5’-TCC ACT GCC CTA CCG AGA AT-3’ (fwd), 5’-AGG GGA GTG CTT CCT ATG CT-3’ (rev); rSOD2 (59.4°C): 5’-CGG GGG CCA TAT CAA TCA CA-3’ (fwd), 5’-TCC AGC AAC TCT CCT TTG GG-3’ (rev); rCCND2 (59.0°C): 5’-CAA GTT TGC CAT GTA CCC GC-3’ (fwd), 5’-GCT TTG AGA CAA TCC ACA TCG G-3’ (rev); rXIAP (61.1°C): 5’-TCA CTT GGG GAA TCT GTG GTA AG-3’ (fwd), 5’-TCC CAG ATG TTT GGA GCT TTT CT-3’ (rev).

#### Isothermal titration calorimetry (ITC)

ITC experiments were conducted on an Affinity ITC instrument (TA Instruments, New Castle, DE, USA) at 25 °C with a stirring rate of 75 rpm. Nurr1 LBD protein (15 μM, expressed as described previously^18^) in buffer (20 mM Tris, pH 7.5, 100 mM NaCl, 5% glycerol) containing 5% DMSO was titrated with **32** (100 μM in the same buffer containing 1–4% DMSO) in 21 injections (1 × 1 µL and 20 × 4 μL) with an injection interval of 120 s. As control experiments, the test compound was titrated to the buffer, and the buffer was titrated to the Nurr1 LBD protein under otherwise identical conditions. The heats of the compound−protein titration were analyzed using NanoAnalyze software (version 3.11.0, TA Instruments) with independent binding model.

#### Nurr1 homodimerization assay

Modulation of Nurr1 LBD homodimerization by **32** was studied in a homogenous time-resolved fluorescence resonance energy transfer (HTRF) based assay. Biotinylated recombinant Nurr1 LBD protein and sGFP-Nurr1 LBD protein (FRET acceptor) were expressed and purified as described previously^28^. Terbium cryptate as streptavidin conjugate (Tb-SA; Cisbio Bioassays, Codolet, France) was used as FRET donor for stable coupling to biotinylated recombinant Nurr1 LBD protein. sGFP-Nurr1 LBD protein was titrated from 0.5 µM against a fixed concentration of Tb-SA (0.375 nM) conjugated Nurr1 LBD protein (0.188 nM). Free sGFP was added to keep the total GFP content stable at 0.5 μM. Assay solutions were prepared in HTRF assay buffer (25 mM HEPES pH 7.5, 10% (m/v) glycerol, 5 mM DTT) supplemented with 0.1% (w/v) CHAPS as well as 1% DMSO with **32** at fixed concentrations or DMSO alone as negative control (apo). The FRET donor complex formed from biotinylated Nurr1 LBD (final concentration 0.188 nM) and Tb-SA (0.375 nM) was kept constant while the concentration of sGFP-labeled protein was varied. Samples were equilibrated at RT for 2 h before fluorescence intensities (FI) after excitation at 340 nm were recorded at 520 nm for sGFP acceptor fluorescence and 620 nm for Tb-SA donor fluorescence on a Tecan SPARK plate reader (Tecan Group Ltd.). FI520 nm was divided by FI620 nm and multiplied with 10,000 to give a dimensionless HTRF signal.

## Supplementary Material

Molecular formula strings

Supporting info.

## Figures and Tables

**Table 1:** Evaluation of diverse carboxylic acid bioisosteres. Data for vidofludimus (7)^17^ and 8^21^ for comparison.

ID	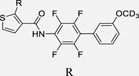	EC_50_ (Nurr1) ^a^ (max. activation)	IC_50_ (DHODH) ^b^	selectivity index DHODH/Nurr1 ^c^	log*P* ^d^	*pK*_a_^e^
**7**	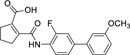	0.4±0.2 μM (3.1±0.4-fold)	0.61±0.07 μM	1.5	3.4	4.6
**8**	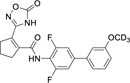	1.0±0.1 μM (2.0±0.1-fold)	9±4 μM	9	4.5	7.7
**12**	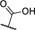	0.005±0.002 μM (2.2±0.1-fold)	0.0019 ± 0.0005 μM	0.4	4.3	2.7
**13**	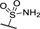	1.1 ± 0.1 μM (2.09 ± 0.08-fold)	3.33±0.06 μM	3.0	3.8	9
**14**	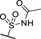	0.9 ± 0.1 μM (1.89 ± 0.06-fold)	4±1 μM	4.4	3.7	4.1
**15**	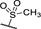	1.2 ± 0.1 μM (2.05 ± 0.08-fold)	49±1% inhibition @100 μM	~100	3.9	-
**16**	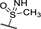	1.2 ± 0.2 μM (2.17 ± 0.11-fold)	76±6% inhibition @100μM	~25	4.0	-
**17**	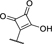	0.10 ± 0.05 μM (1.97 ± 0.43-fold)	0.7±0.5 μM	7	4.6	1.1
**18**	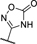	0.05 ± 0.01 μM (1.86 ± 0.11-fold)	0.66±0.08 μM	13	4.1	5.7
**19**	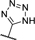	0.021 ± 0.002 μM (2.14 ± 0.11-fold)	0.6±0.2 μM	28	3.6	3.7

a Nurr1 modulation (mean ± S.E.M., n ≥ 3) was determined in a Gal4-Nurr1 hybrid reporter gene assay^28^.

b DHODH inhibition (mean ± S.E.M., n ≥ 3) was determined in a colorimetric assay on recombinant human protein^20^.

c The selectivity index refers to (IC_50_(DHODH) / EC_50_(Nurr1)).

d Log*P* was predicted with ALOGPS^29^.

e p*K*_a_ values were predicted with MolGpka^30^ and only provide an estimate but aligned with the experimentally determined^26,31^ relative order.

**Table 2:** SAR of polar heterocycles as bioisosters.

ID	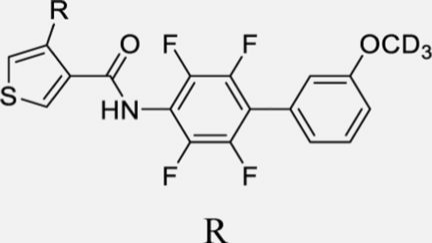	EC_50_ (Nurr1) ^a^ (max. activation)	IC_50_ (DHODH)^b^	selectivity index DHODH/Nurr1 ^c^	log*P* ^d^	*pK*_a_^e^
**20**	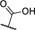	0.0025±0.0001 μM (2.2±0.1-fold)	0.0022±0.0002 μM	0.9	4.3	3.6
**21**	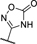	0.03±0.01 μM (2.2±0.1-fold)	0.40±0.06 μM	13	4.1	6.7
**22**	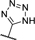	0.011±0.002 μM (2.5±0.2-fold)	0.043±0.006 μM	3.9	3.6	4.3
**23**	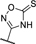	0.036±0.003 μM (1.77±0.04-fold)	0.062±0.004 μM	1.7	4.9	5.7
**24**	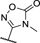	2.6±0.3 μM (1.9±0.2-fold)	11.1±0.6 μM	4.2	4.5	-
**25**	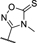	0.9±0.1 μM (2.3±0.2-fold)	40±8% inhibition @100 μM	>100	5.1	-
**26**	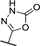	0.017±0.002 μM (1.8±0.1-fold)	0.10±0.01 μM	5.9	3.9	6.3
**27**	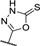	0.16±0.05 μM (1.7±0.1-fold)	0.96±0.09 μM	6.0	4.5	5.1
**28**	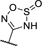	0.039±0.005 μM (2.4±0.1-fold)	1.2±0.2 μM	30	3.7	4.6

a Nurr1 modulation (mean ± S.E.M., n ≥ 3) was determined in a Gal4-Nurr1 hybrid reporter gene assay^28^.

b DHODH inhibition (mean ± S.E.M., n ≥ 3) was determined in a colorimetric assay on recombinant human protein^20^.

c The selectivity index refers to (IC_50_(DHODH) / EC_50_(Nurr1)).

d Log*P* was predicted with ALOGPS^29^.

e p*K*_a_ values were predicted with MolGpka^30^ and only provide an estimate but aligned with the experimentally determined^26,31^ relative order.

**Table 3:** Scaffold variation in tetrazole-based Nurr1 agonists.

ID	structure	EC50 (Nurr1) ^a^ (max. activation)	IC_50_ (DHODH)^b^	selectivity index DHODH/Nurr1 ^c^	log*P* ^d^	*pK*_a_^e^
**29**	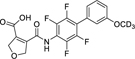	0.5±0.01 μM (2.5±0.1-fold)	0.06±0.02 μM	0.12	3.5	3.2
**30**	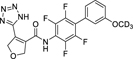	(0.22±0.05-μM (2.0±0.2-fold)	3±1 μM	13.6	2.5	4.5
**11**	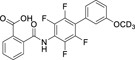	0.06±0.02 μM (2.1±0.2-fold)	0.015±0.003 μM	0.3	4.5	3.4
**31**	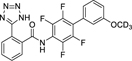	0.14±0.01 μM (1.9±0.1-fold)	0.26±0.04 μM	1.9	3.7	3.8
**7** (vidofludimus)	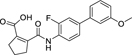	(0.4±0.2-μM) (3.1±0.4-fold)	0.61±0.07 μM	1.5	3.4	4.6
**32**	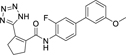	0.09±0.01 μM (2.5±0.1-fold)	10±2 μM	110	3.3	4.6

a Nurr1 modulation (mean ± S.E.M., n ≥ 3) was determined in a Gal4-Nurr1 hybrid reporter gene assay^28^.

b DHODH inhibition (mean ± S.E.M., n ≥ 3) was determined in a colorimetric assay on recombinant human protein^20^.

c The selectivity index refers to (IC_50_(DHODH) / EC_50_(Nurr1)).

d Log*P* was predicted with ALOGPS^29^.

e p*K*_a_ values were predicted with MolGpka^30^ and only provide an estimate but aligned with the experimentally determined^26,31^ relative order.

## Abbreviations

AD: Alzheimer’s disease
BDNF: brain-derived neurotrophic factor
BIRG5: baculoviral inhibitor of apoptosis repeat-containing 5
CAR: constitutive androstane receptor
CCND2: cyclin D2
CRMP4: collapsin response mediator protein 4
DHODH: dihydroorotate dehydrogenase
FLRT2: fibronectin leucine rich transmembrane protein 2
FXR: farnesoid X receptor
HTRF: homogenous time-resolved fluorescence resonance energy transfer
ITC: isothermal titration calorimetry
LBD: ligand binding domain
NOR1: neuron derived orphan receptor 1
Nur77: nerve growth factor IB
Nurr1: nuclear receptor related 1
PD: Parkinson’s disease
PPAR: peroxisome proliferator-activated receptor
PXR: pregnane X receptor
RAR: retinoic acid receptor
RXR: retinoid X receptor
Sesn3: sestrin 3
SOD2: superoxide dismutase 2
TH: tyrosine hydroxylase
THR: thyroid hormone receptor
VDR: vitamin D receptor
XIAP: X-linked inhibitor of apoptosis protein
