# Supplementary material for: Carboxylic Acid Bioisosteres Boost Nurr1 Agonist Selectivity
Source: J Med Chem. Author manuscript; Available in PMC 2025 Jul 30. (PMC7617969; doi:10.1021/acs.jmedchem.5c01140)
Supplement: Supporting info. [file EMS207341-supplement-Supporting_info_.pdf]

- Supporting Information -

Carboxylic acid bioisosteres boost Nurr1 agonist selectivity

Tanja Stiller[1], Christian Gege[2], Wael Saeb[3], Jan Vietor[1], Úrsula López-García[1], Romy Busch[1], Hella Kohlhof[2], Daniel Vitt[2], and Daniel Merk[1]*

[1] Ludwig-Maximilians-Universität (LMU) München, Department of Pharmacy, 81377 Munich, Germany

[2] Immunic AG, 82166 Gräfelfing, Germany

[3] RebisLab R&D GmbH, 82152 Planegg-Martinsried, Germany

* daniel.merk@cup.lmu.de

Table of Contents

NMR spectra, MS spectra and LCMS data of **11**, **13–19, 21–28, 30–32** S2

NMR spectra, MS spectra and LCMS data of **11, 13–19, 21–28, 30–32**

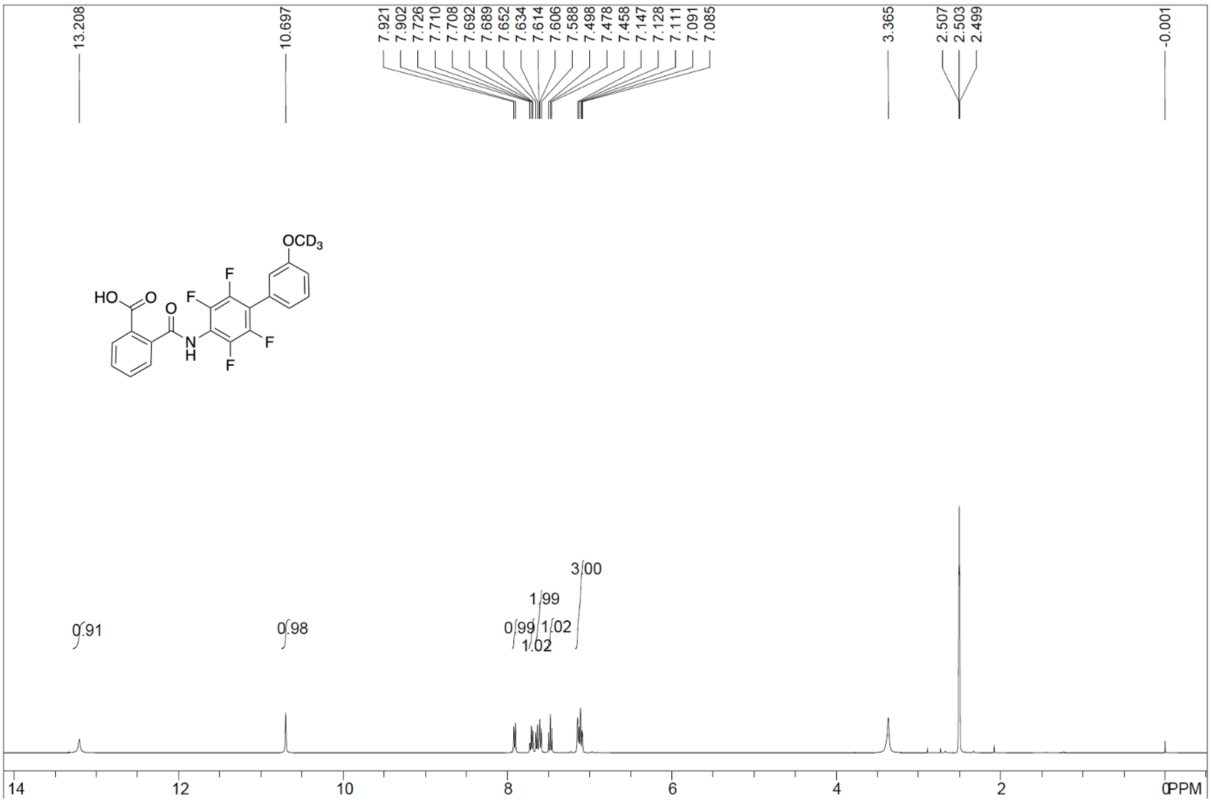

^{1}H-NMR (400 MHz, DMSO-d_6) of compound **11**

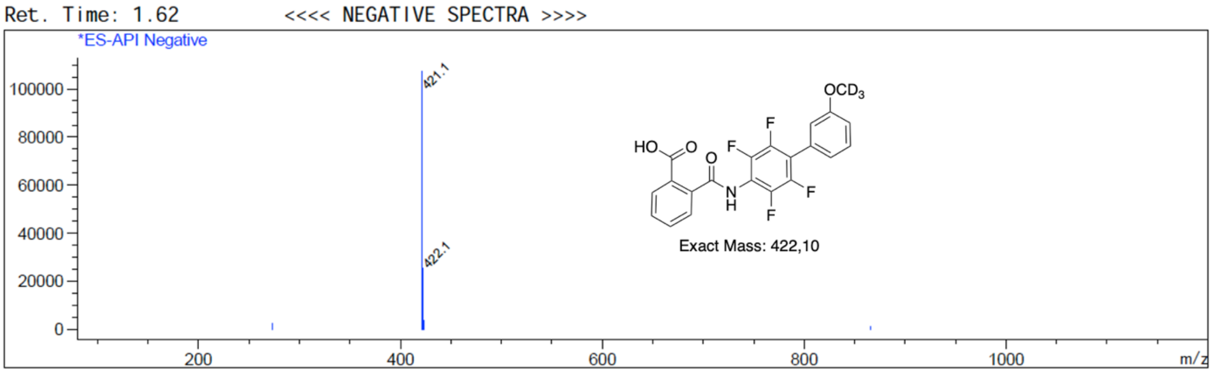

MS of compound **11**

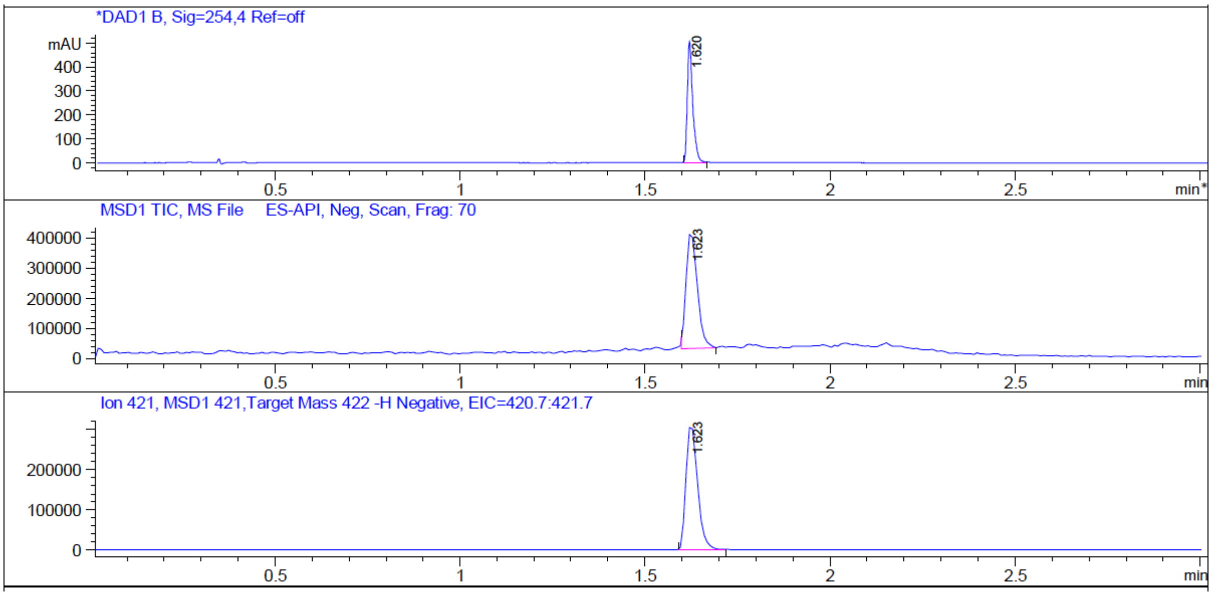

Chromatographic purity analysis of compound **11**

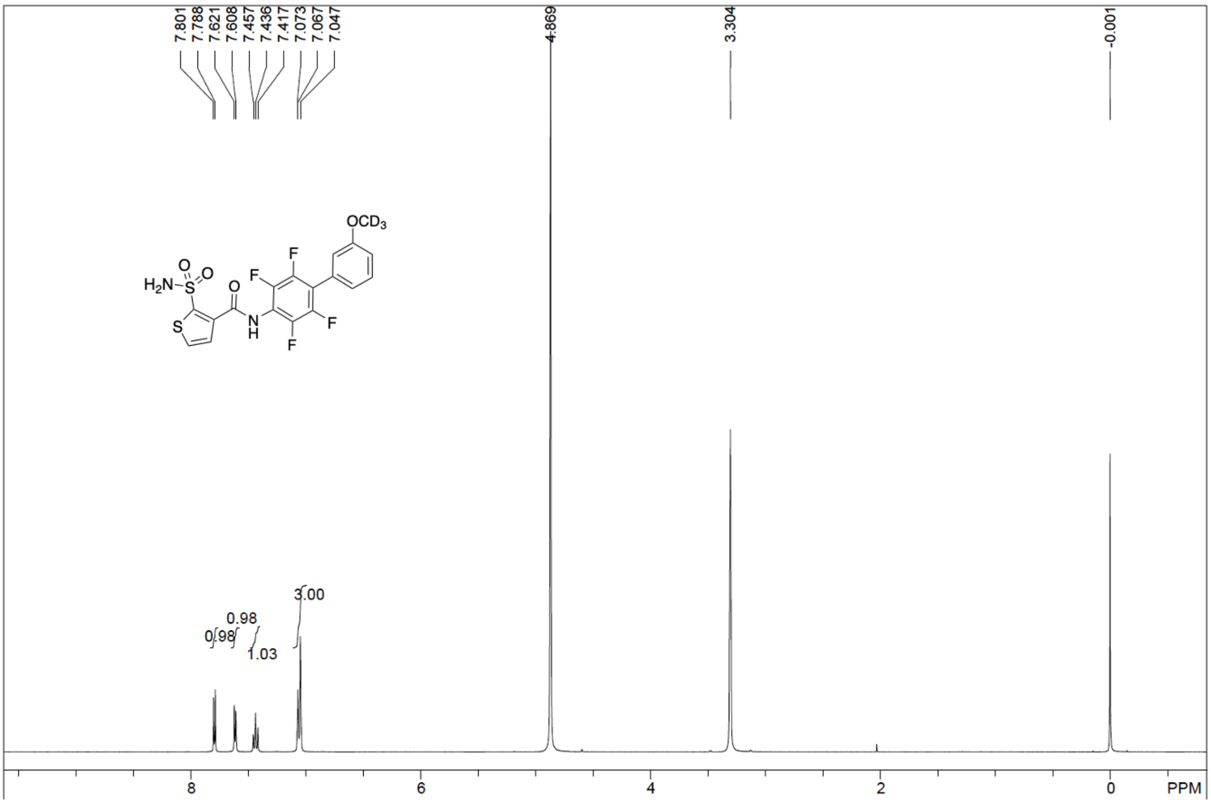

^{1}H-NMR (400 MHz, CD_3OD) of compound **13**

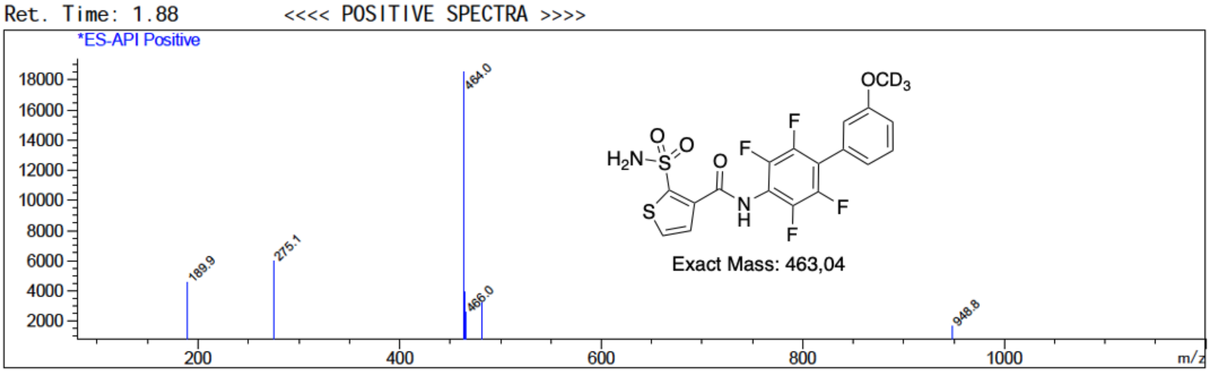

MS of compound **13**

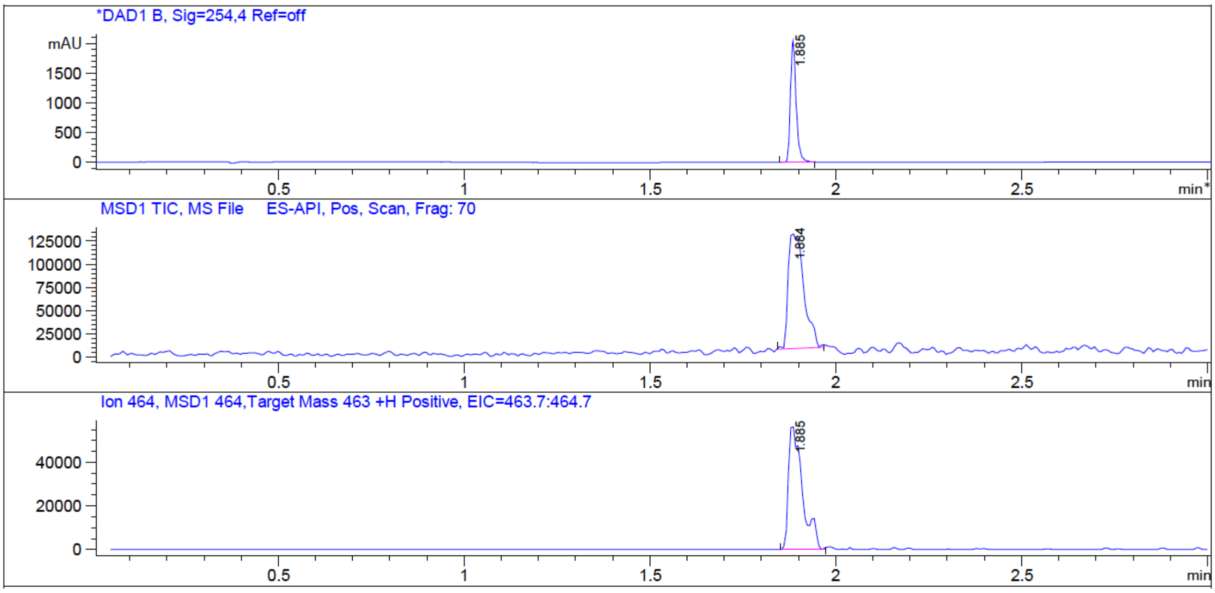

Chromatographic purity analysis of compound **13**

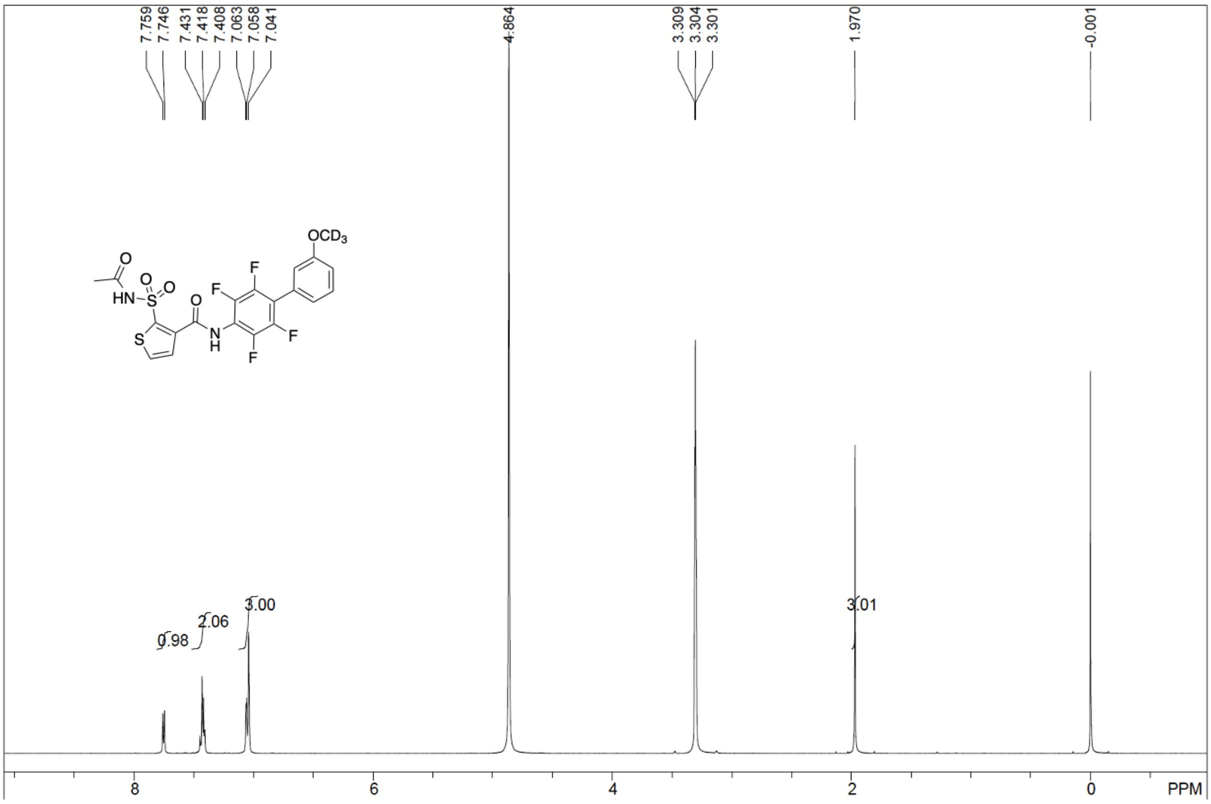

^{1}H-NMR (400 MHz, CD_3OD) of compound **14**

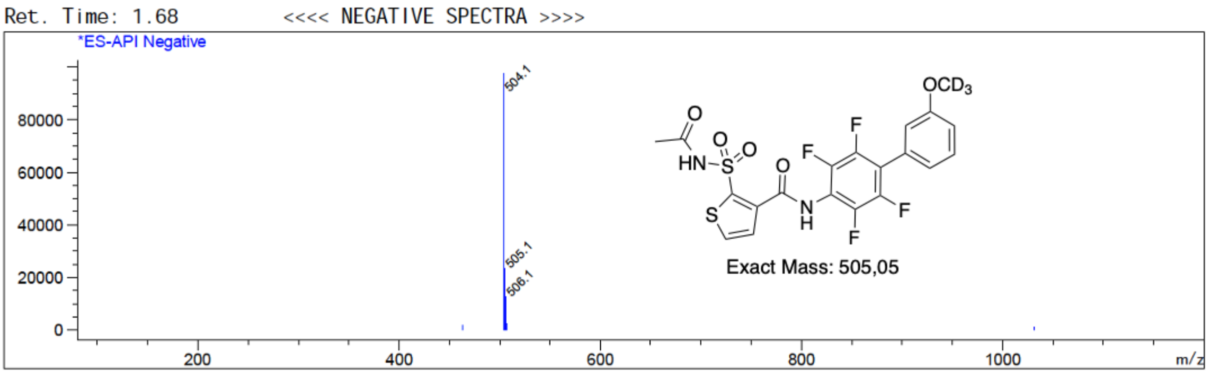

MS of compound **14**

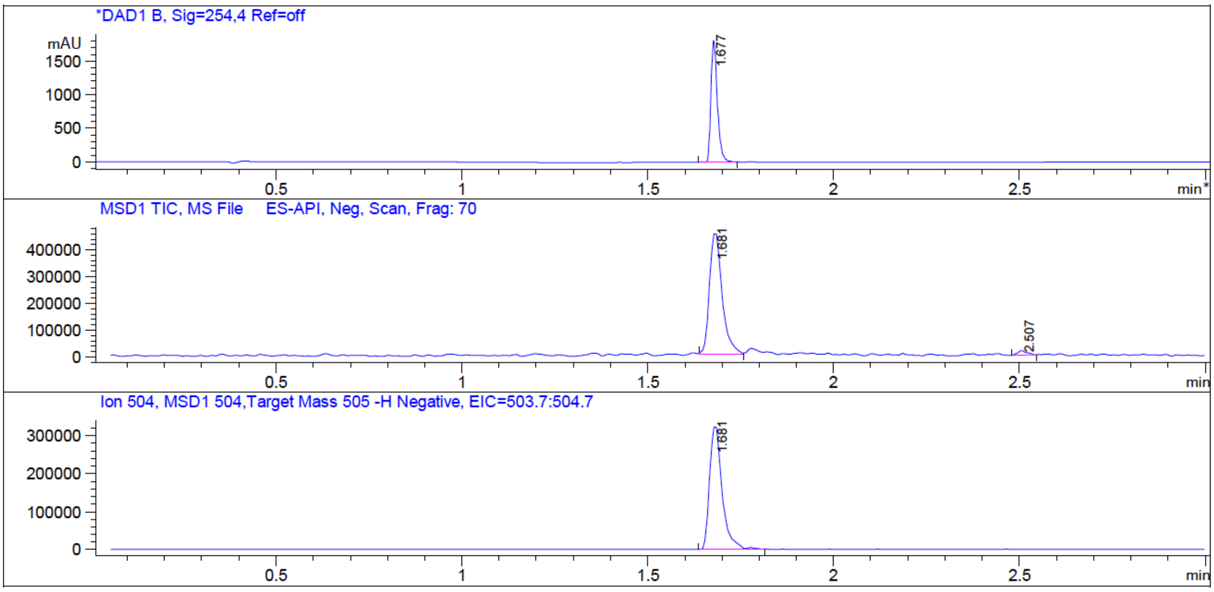

Chromatographic purity analysis of compound **14**

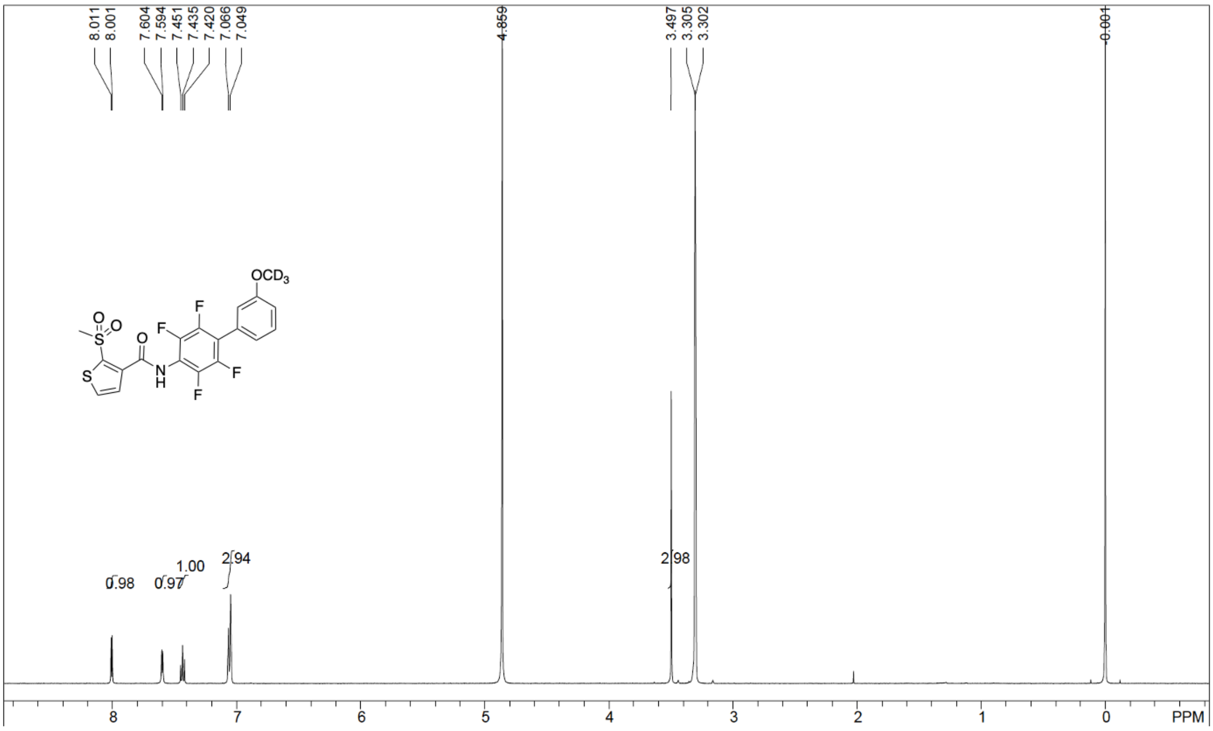

^{1}H-NMR (500 MHz, CD_3OD) of compound **15**

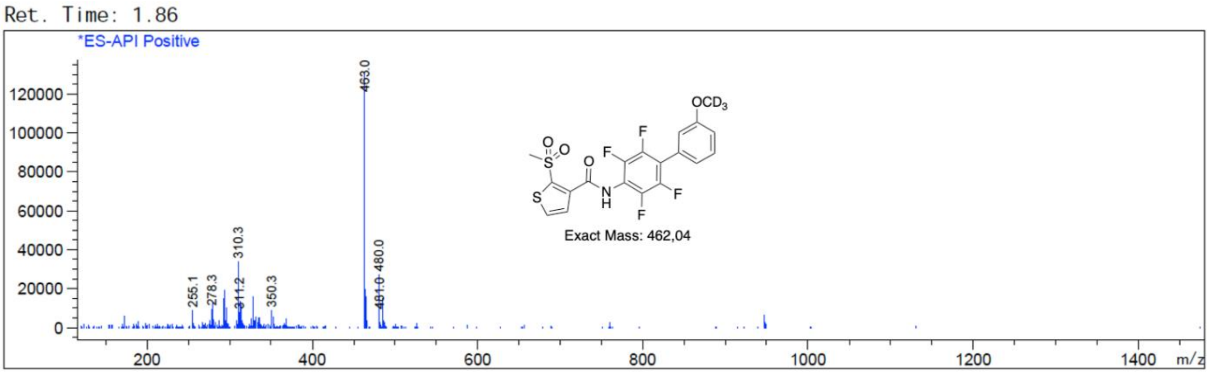

MS of compound **15**

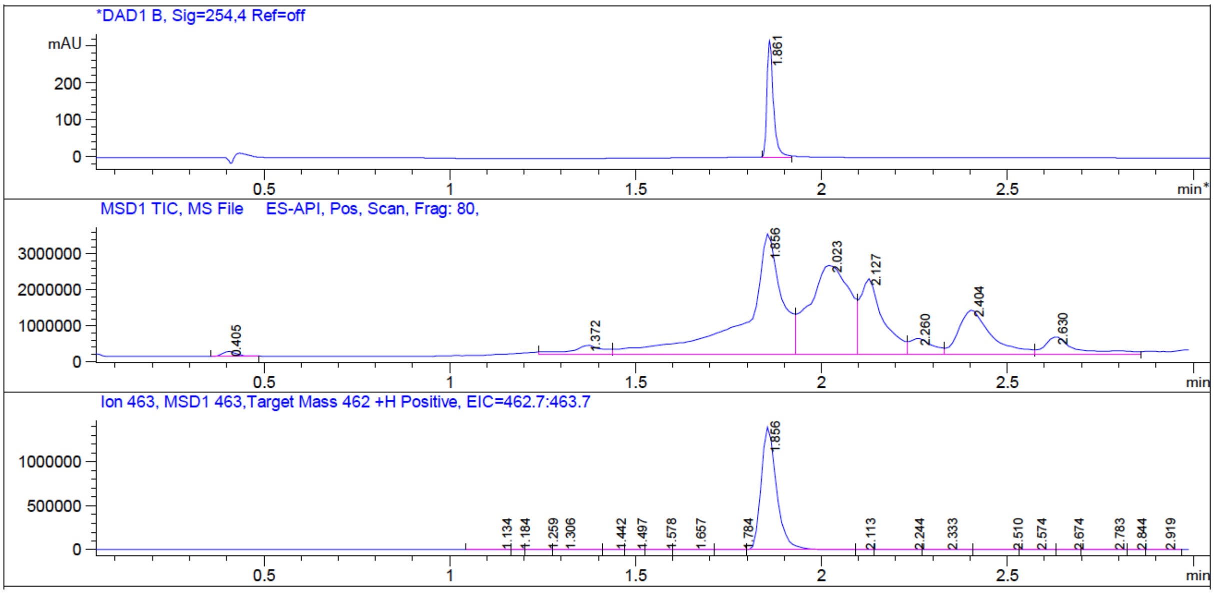

Chromatographic purity analysis of compound **15**

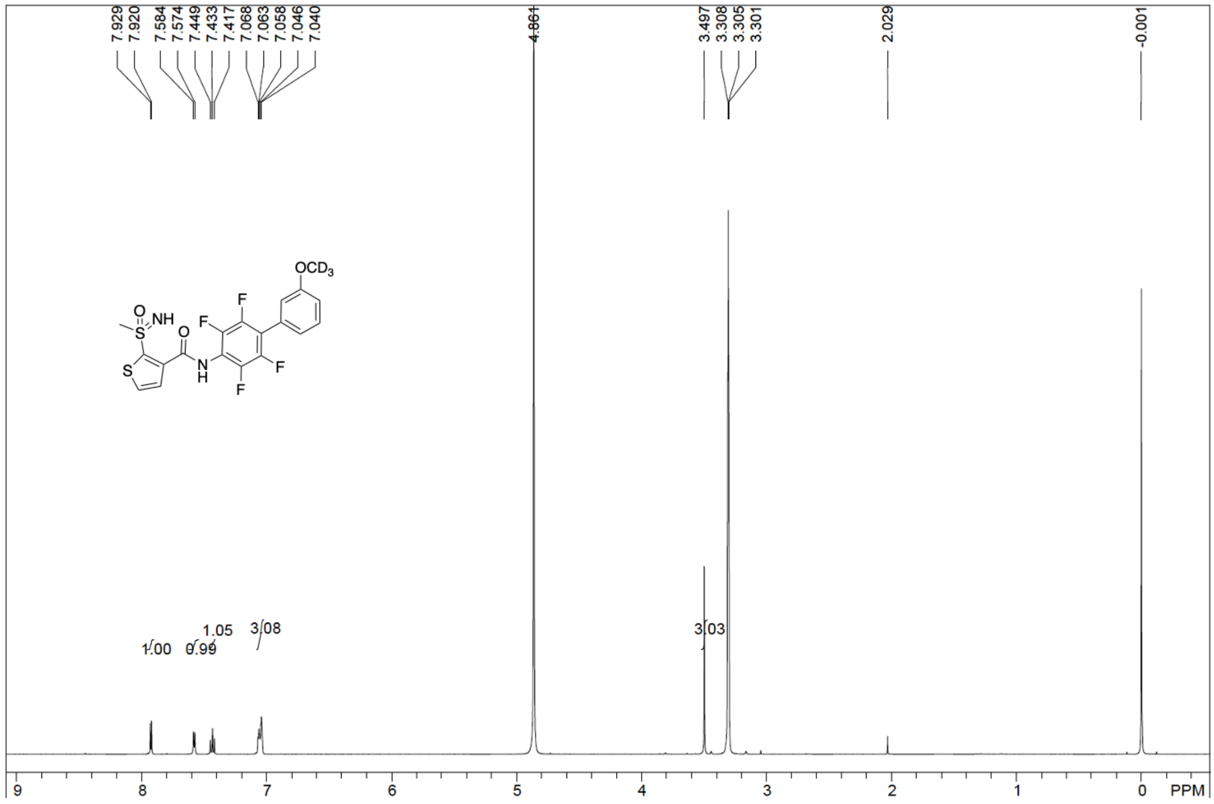

^{1}H-NMR (500 MHz, CD_3OD) of compound **16**

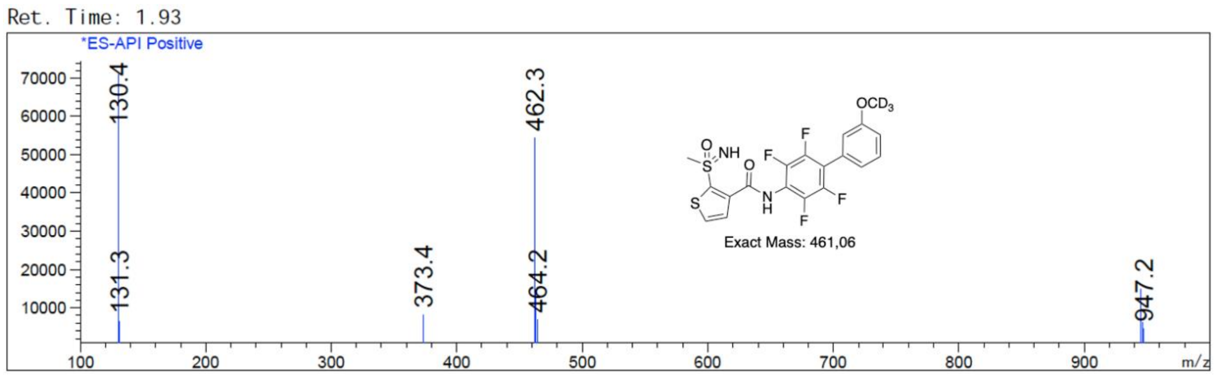

MS of compound **16**

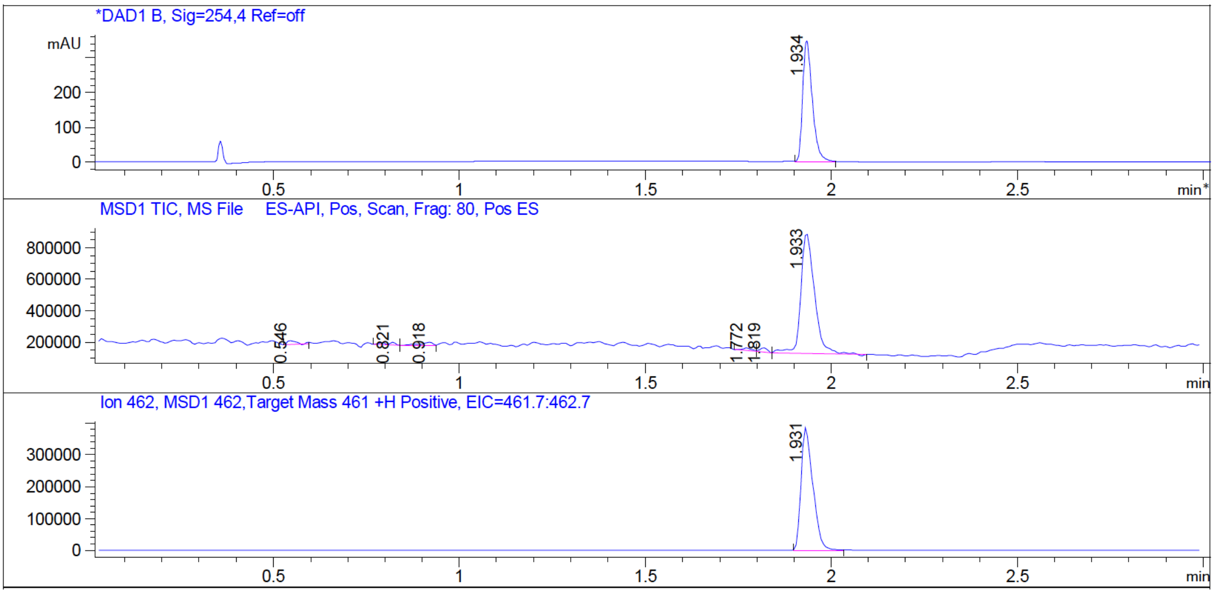

Chromatographic purity analysis of compound **16**

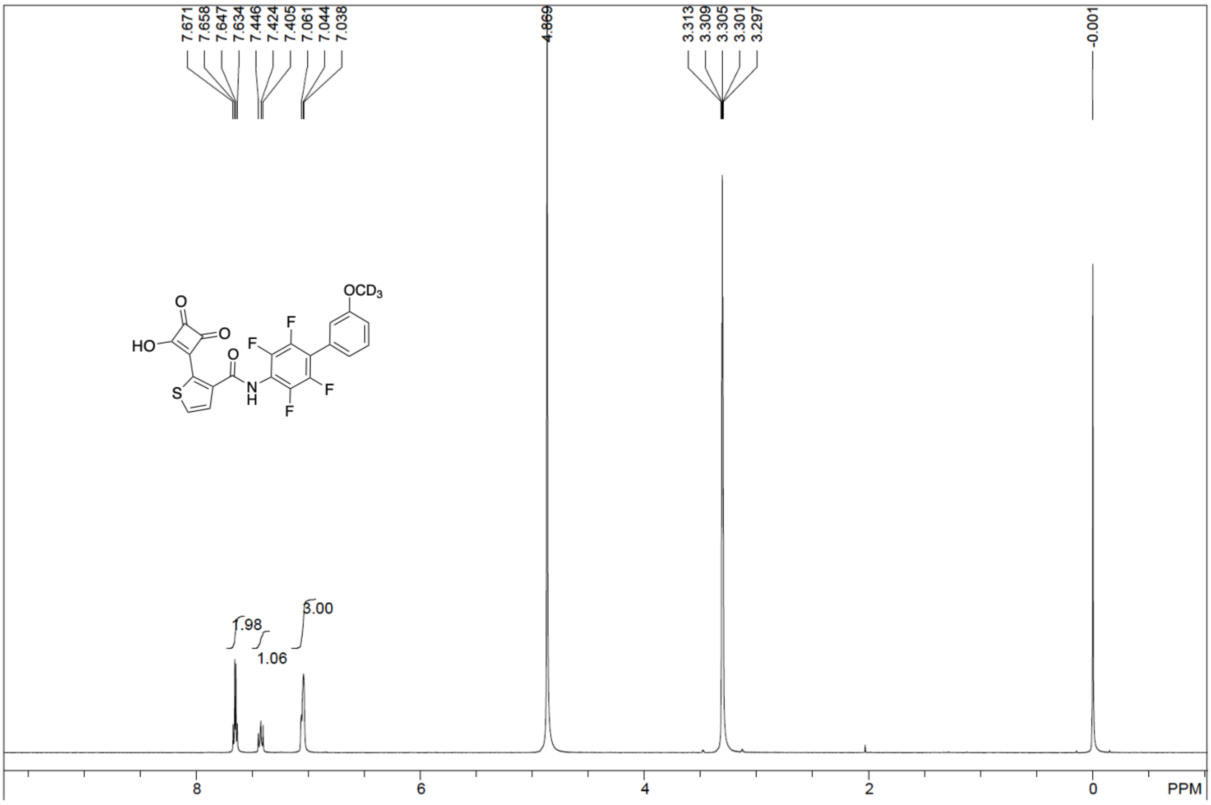

^{1}H-NMR (400 MHz, CD_3OD) of compound **17**

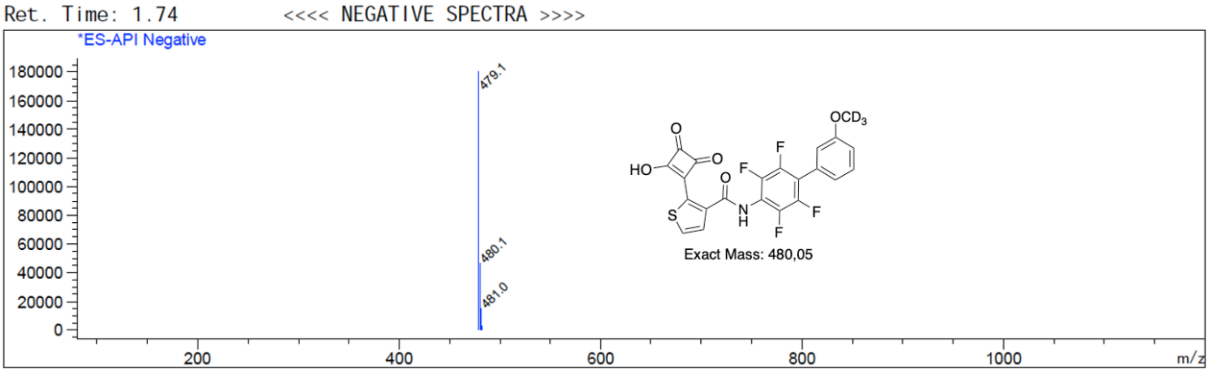

MS of compound **17**

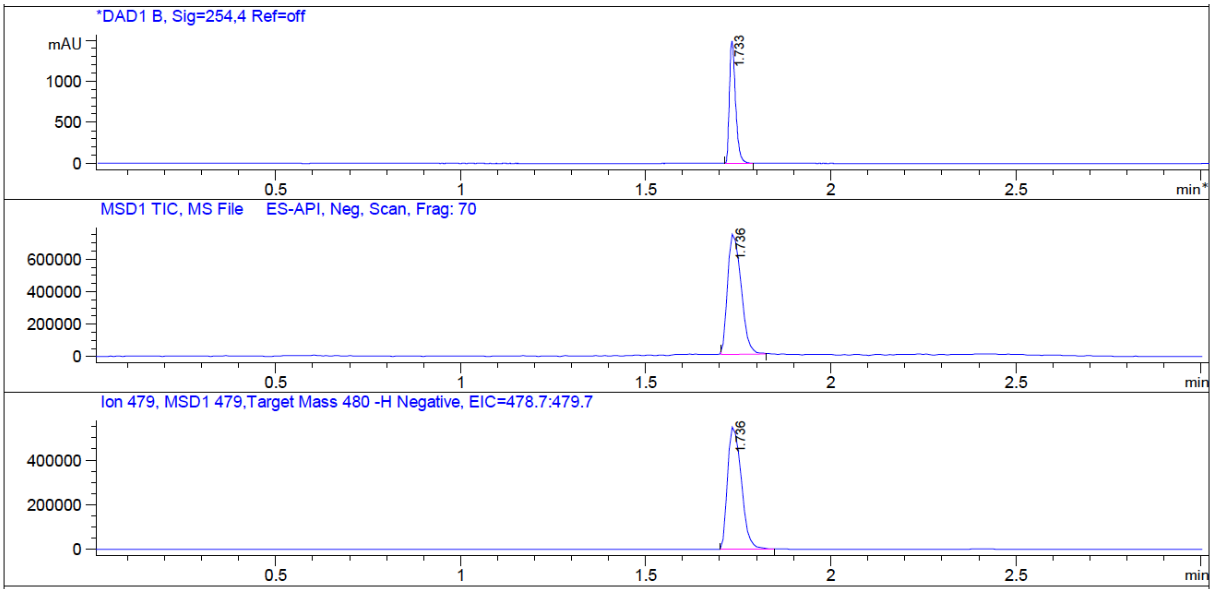

Chromatographic purity analysis of compound **17**

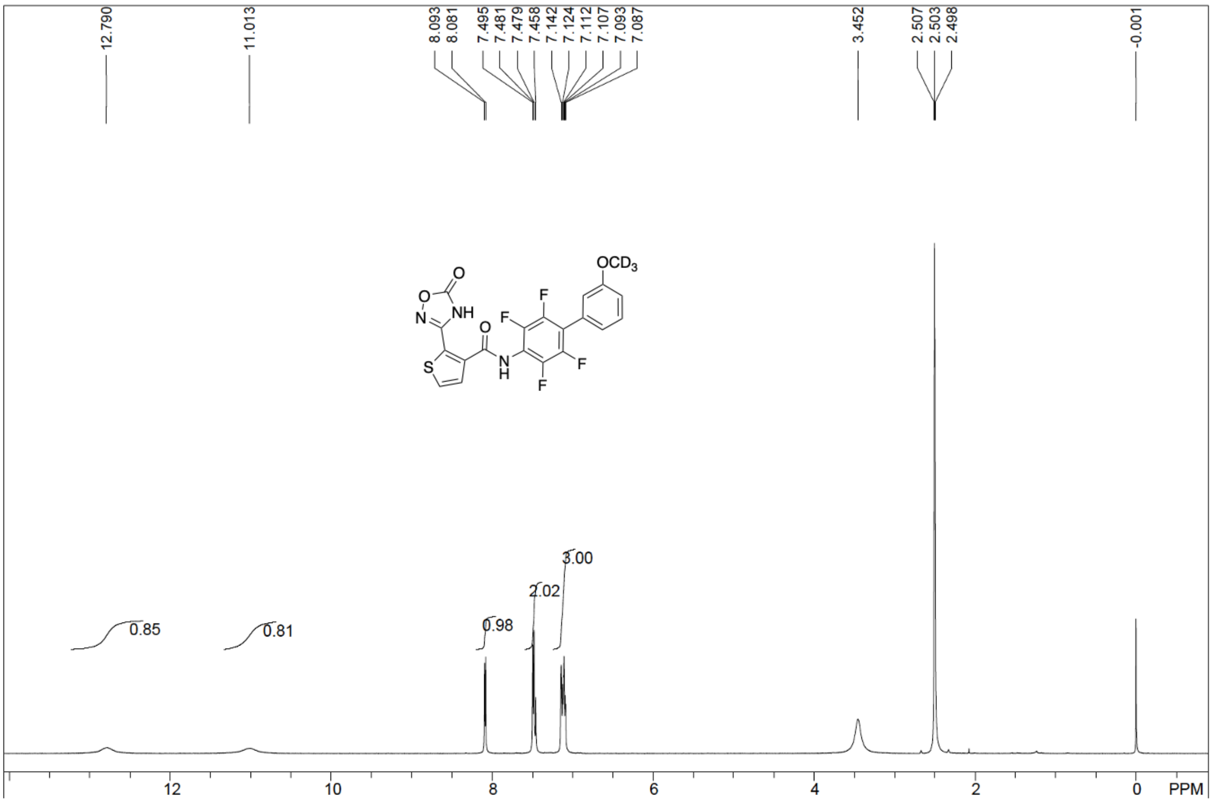

^{1}H-NMR (400 MHz, DMSO-d_6) of compound **18**

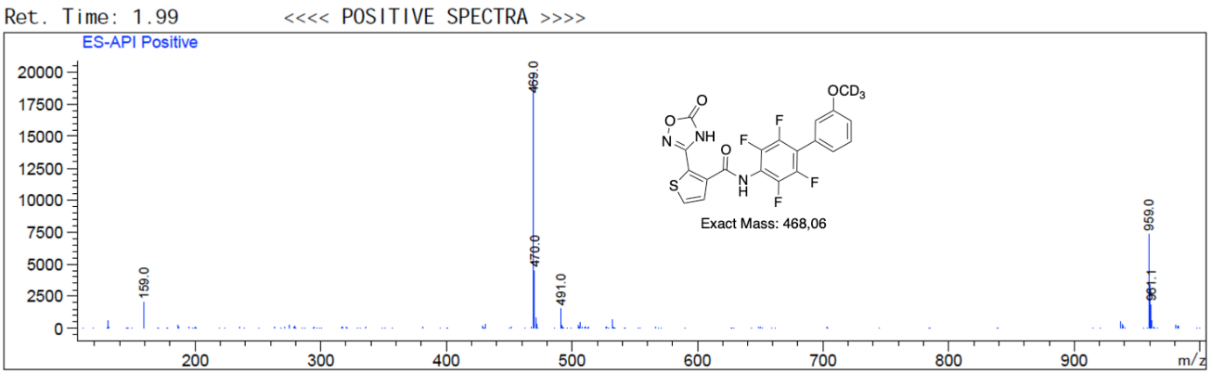

MS of compound **18**

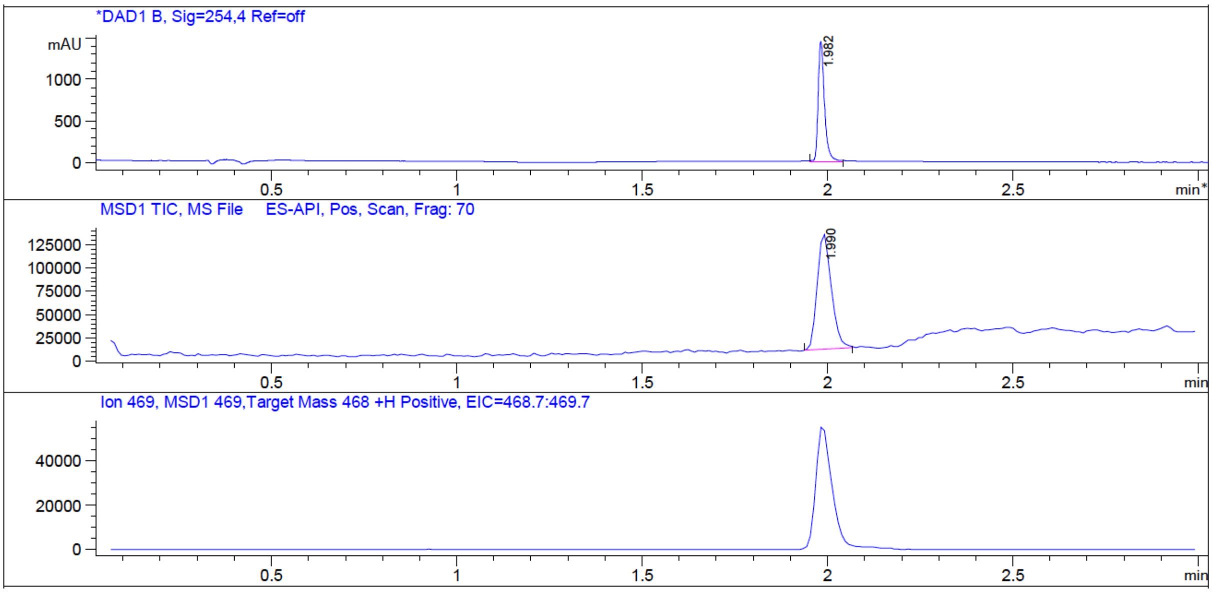

Chromatographic purity analysis of compound **18**

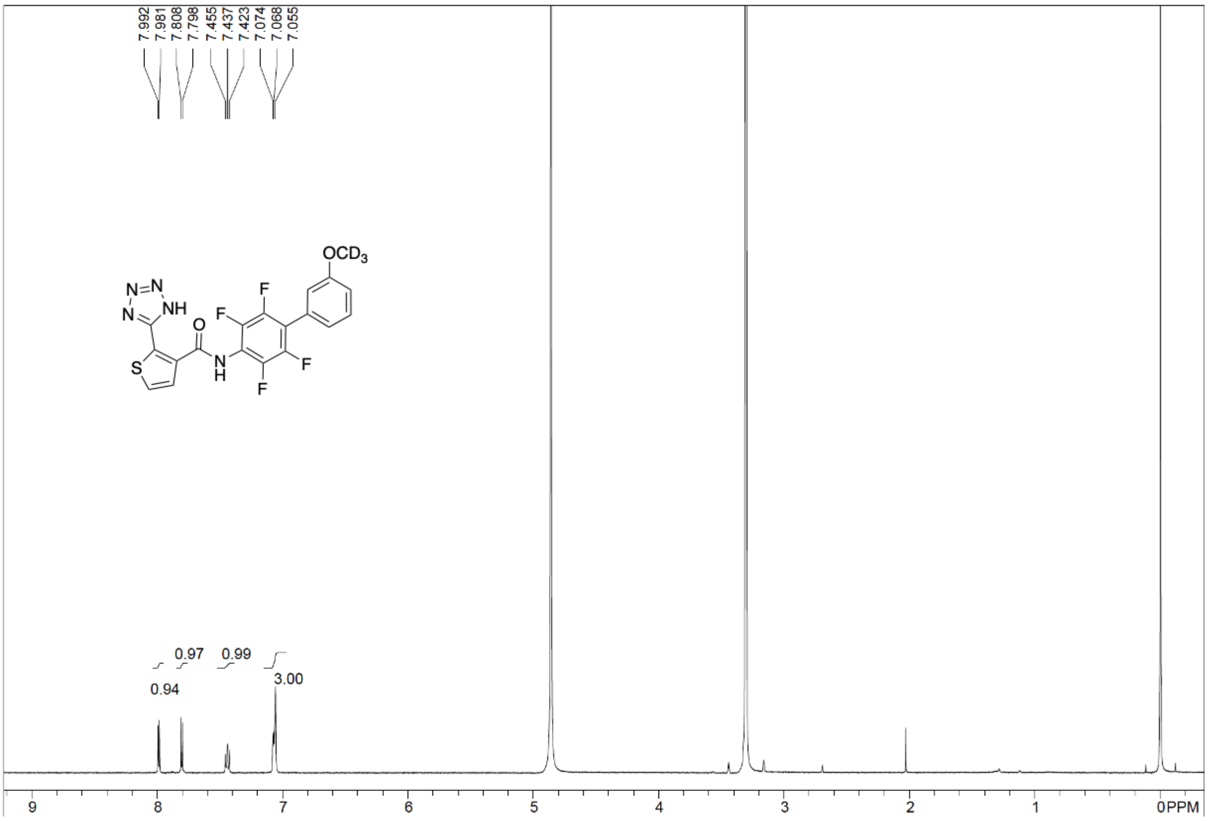

^{1}H-NMR (500 MHz, CD_3OD) of compound **19**

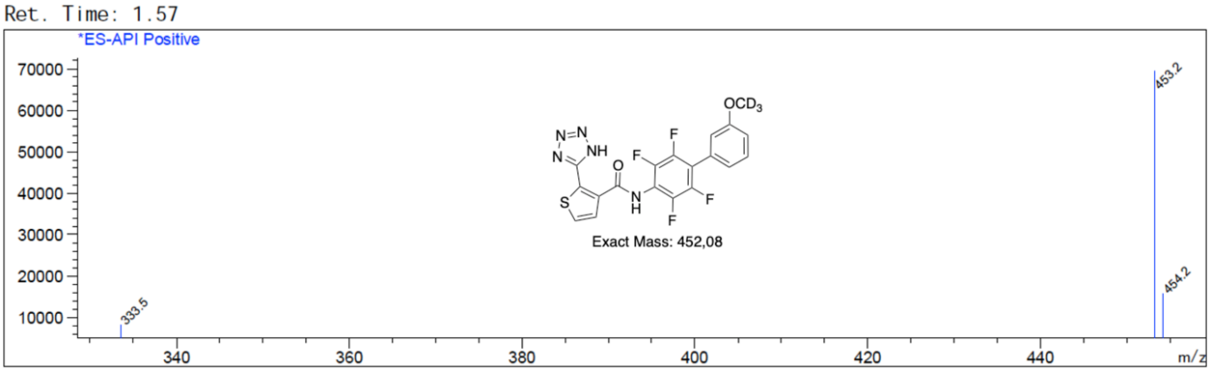

MS of compound **19**

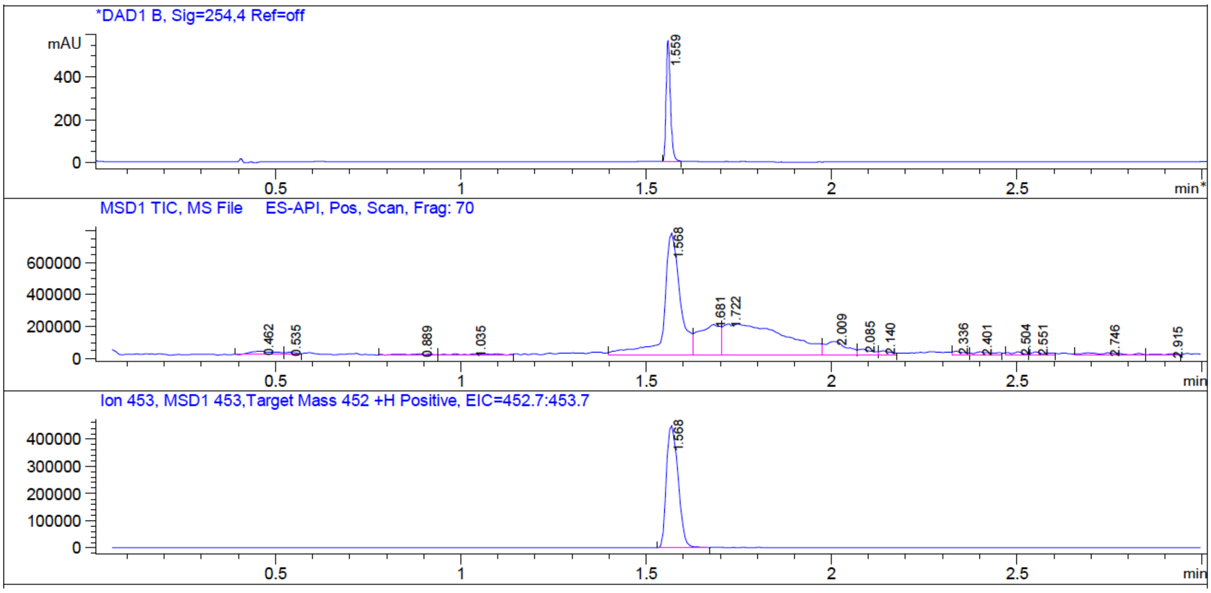

Chromatographic purity analysis of compound **19**

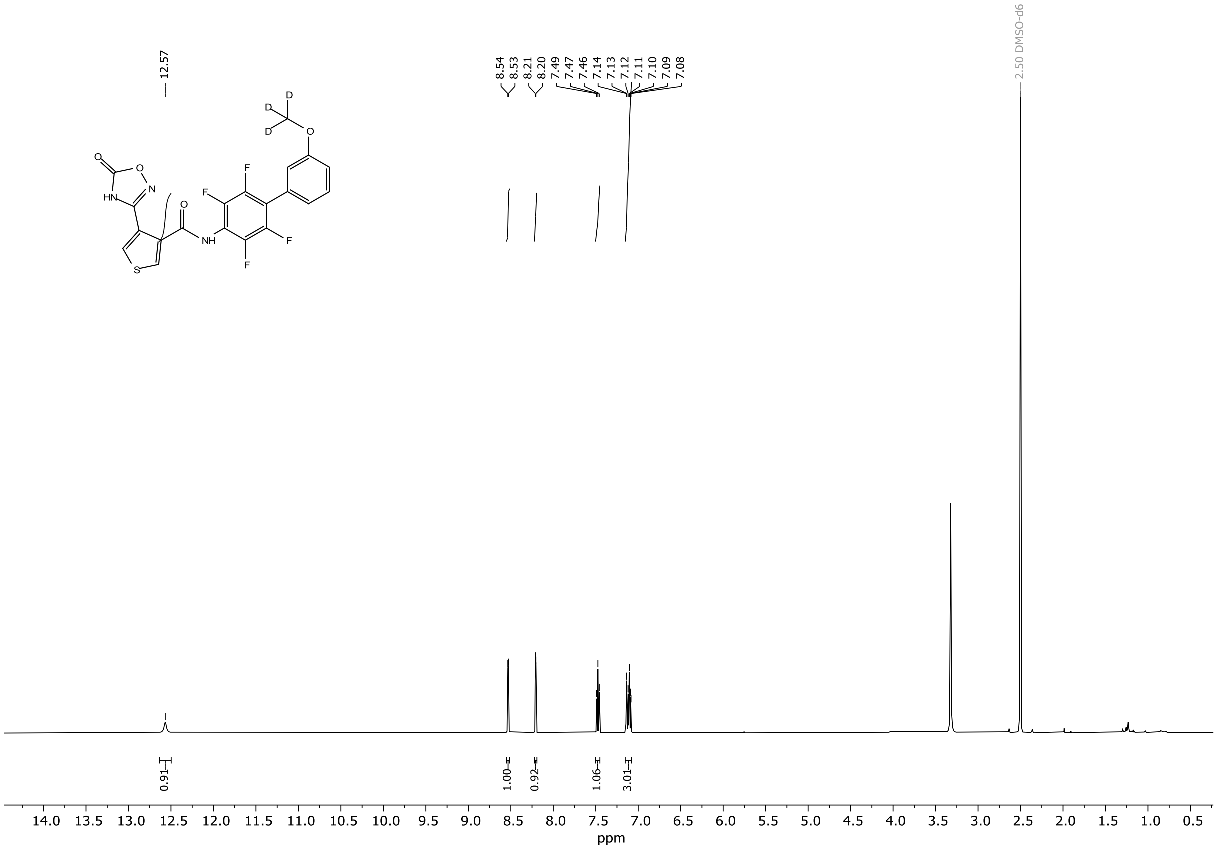

^{1}H-NMR (500 MHz, DMSO-d_6) of compound **21**

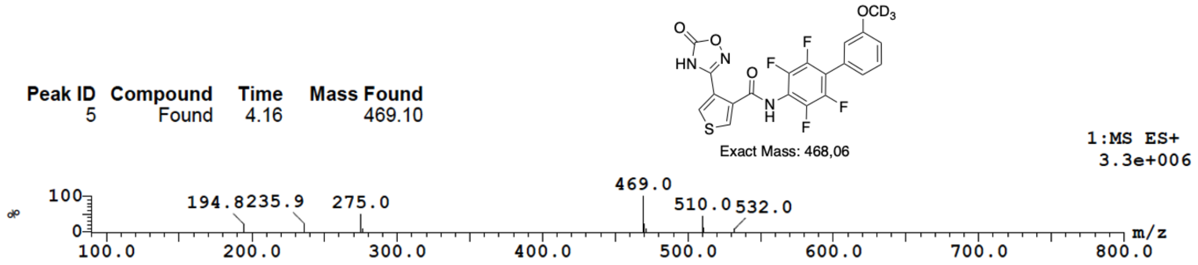

MS of compound **21**

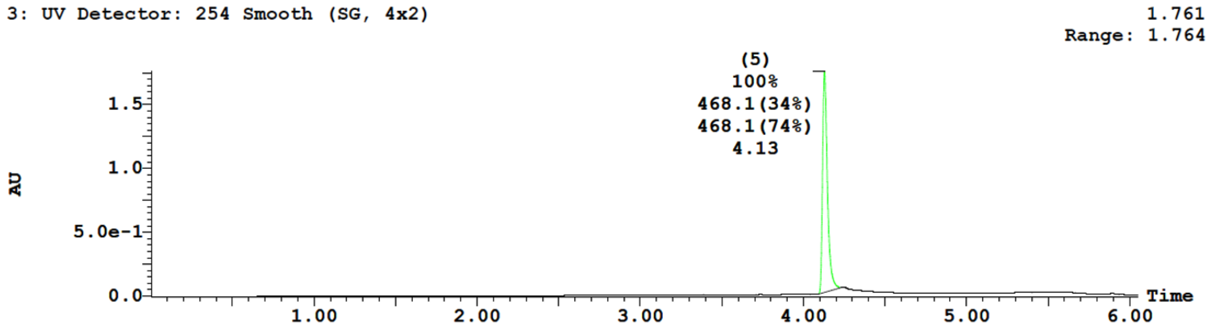

Peak Number	Compound	Time	AreaAbs	Area %Total	Width	Height	Mass Found
5	Found	4.13	6e+004	100.00	0	2e+006	468.10, 468.10

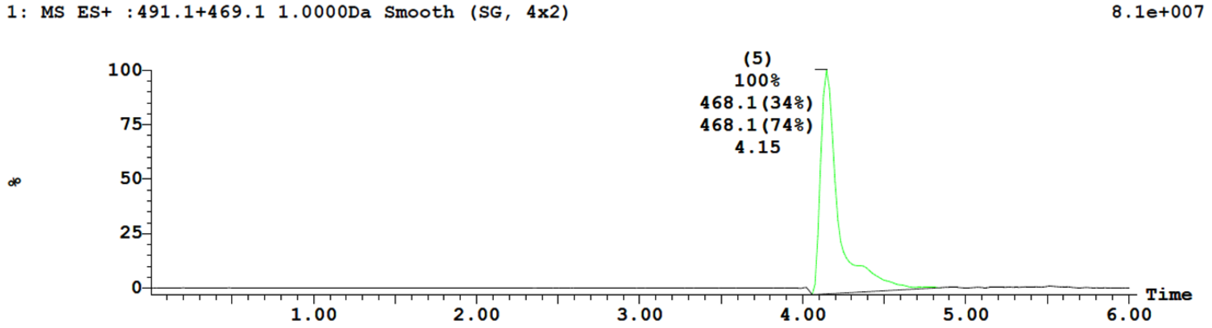

Peak Number	Compound	Time	AreaAbs	Area %Total	Width	Height	Mass Found
5	Found	4.15	1e+007	100.00	1	8e+007	468.10, 468.10

Chromatographic purity analysis of compound **21**

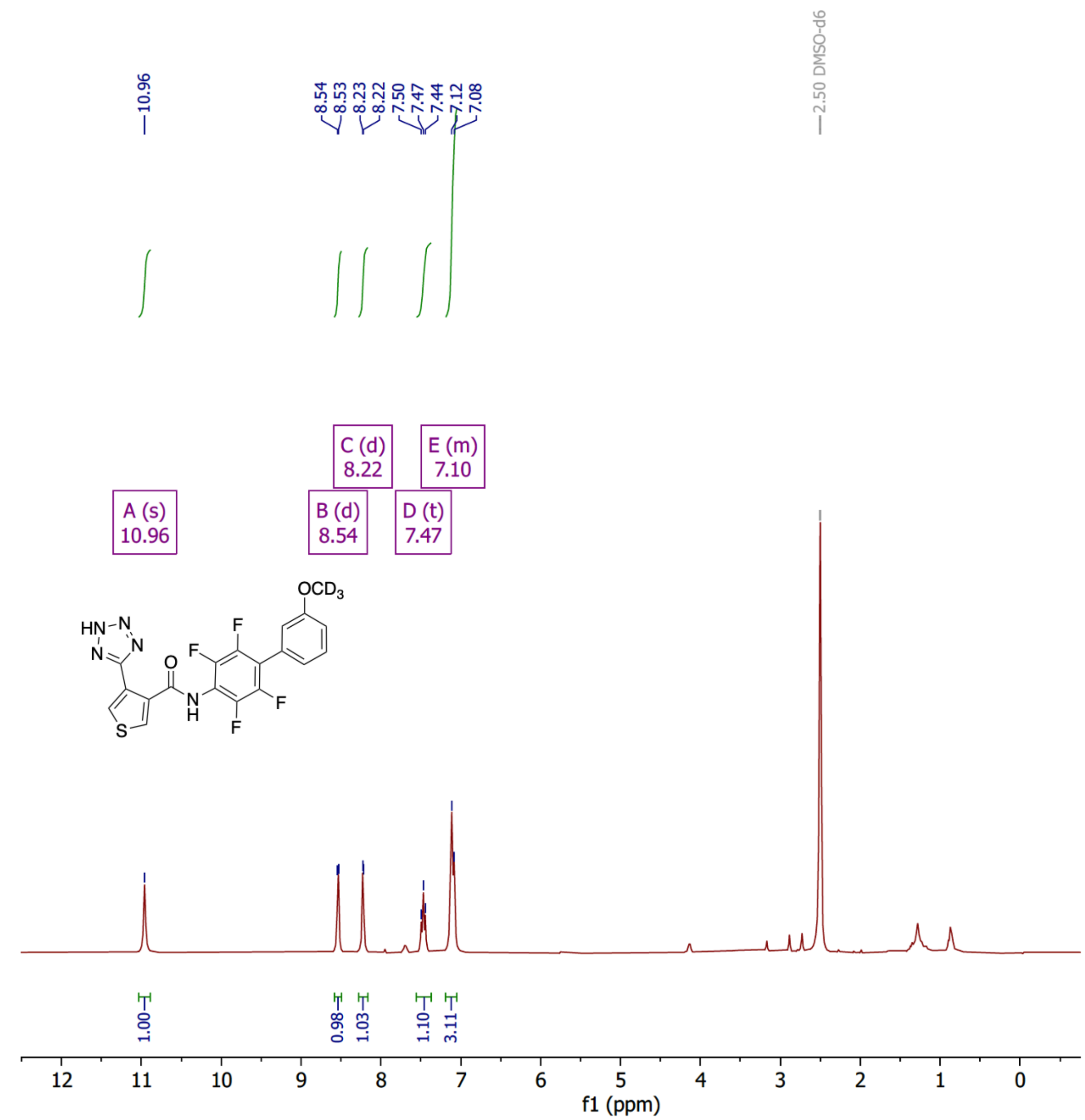

^{1}H-NMR (300 MHz, DMSO-d_6) of compound **22**

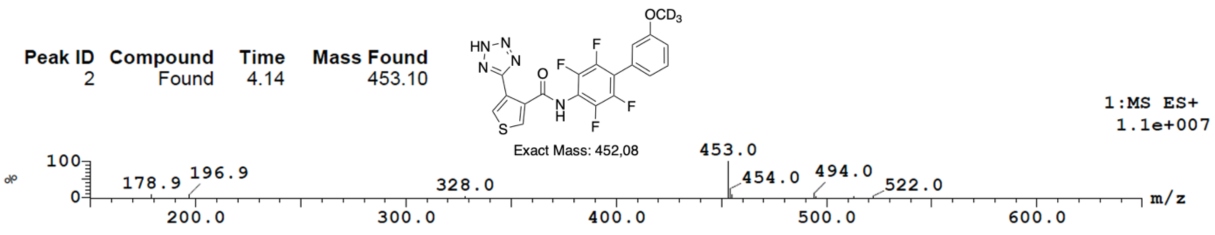

MS of compound **22**

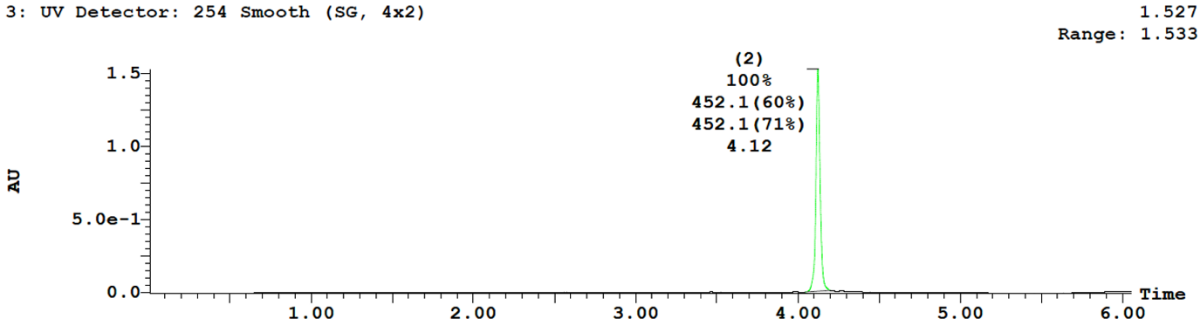

Peak Number	Compound	Time	AreaAbs	Area %Total	Width	Height	Mass Found
2	Found	4.12	5e+004	100.00	0	2e+006	452.10, 452.10

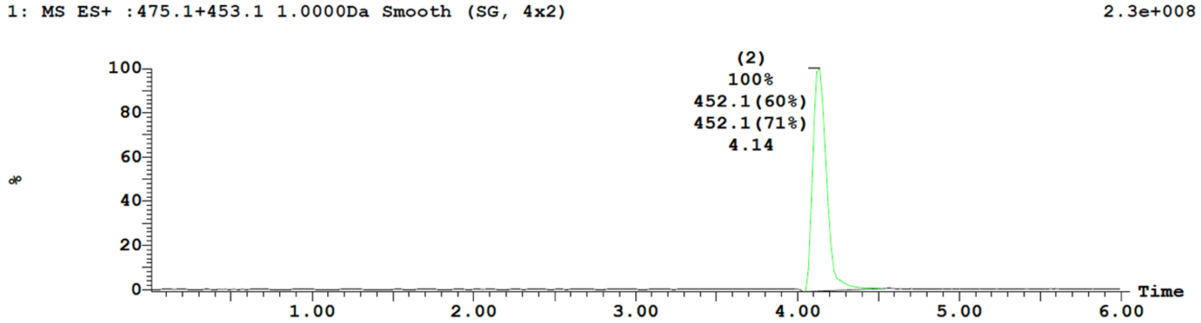

Peak Number	Compound	Time	AreaAbs	Area %Total	Width	Height	Mass Found
2	Found	4.14	2e+007	100.00	0	2e+008	452.10, 452.10

Chromatographic purity analysis of compound **22**

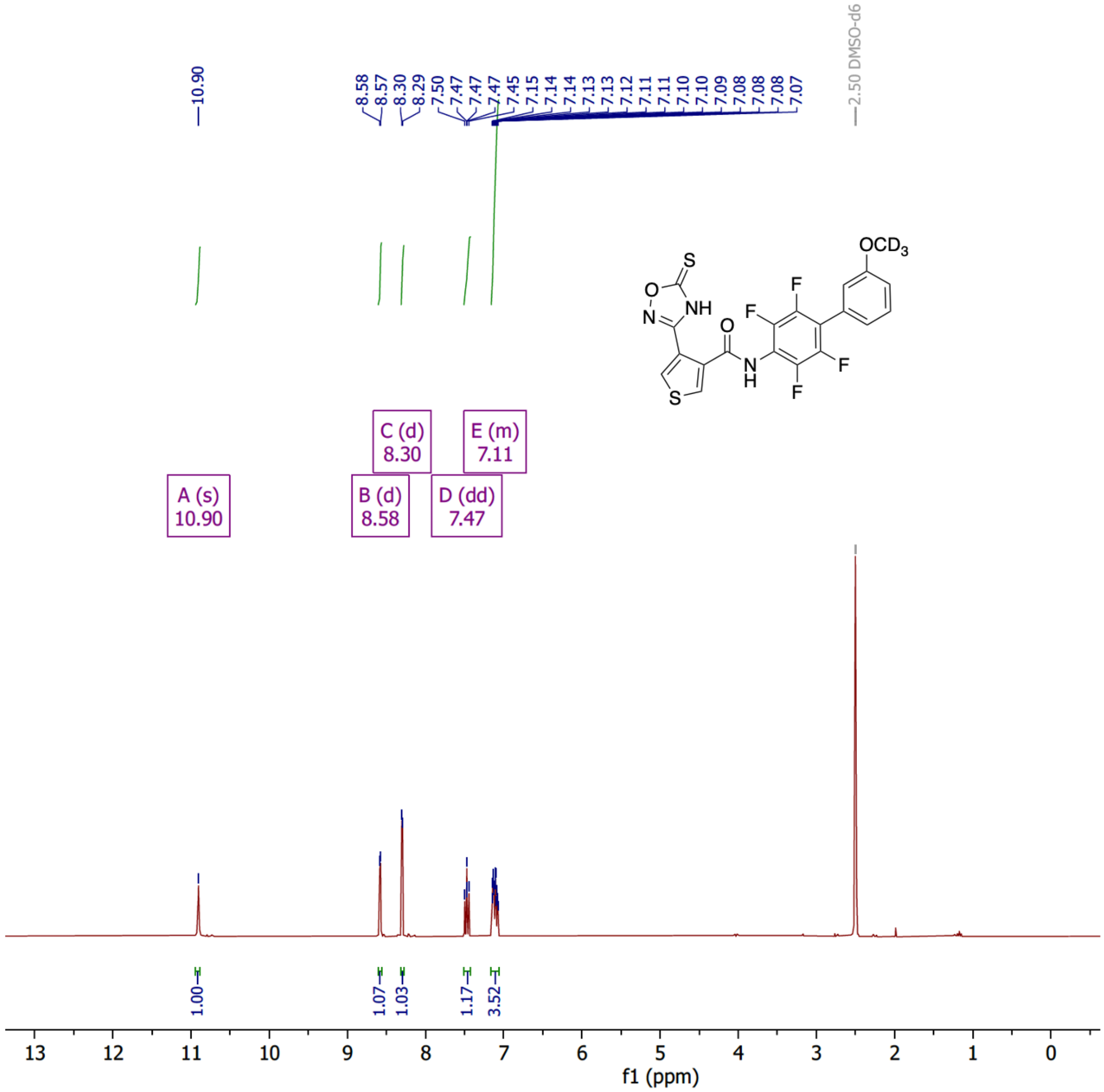

^{1}H-NMR (300 MHz, DMSO-d_6) of compound **23**

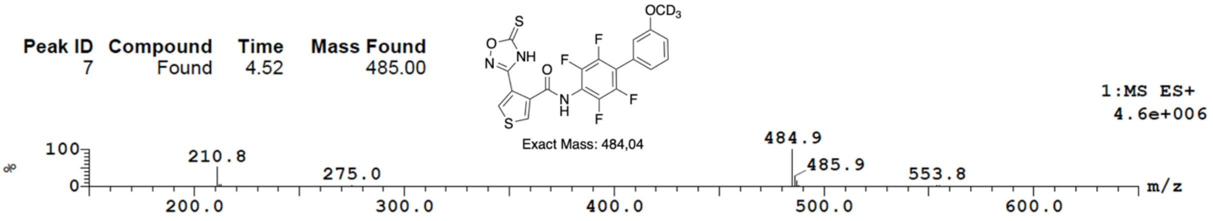

MS of compound **23**

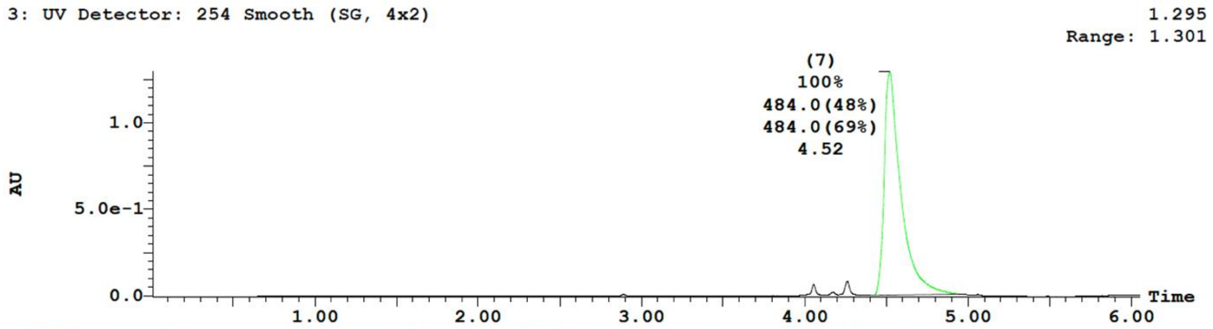

Peak Number	Compound	Time	AreaAbs	Area %Total	Width	Height	Mass Found
7	Found	4.52	2e+005	100.00	1	1e+006	484.00, 484.00

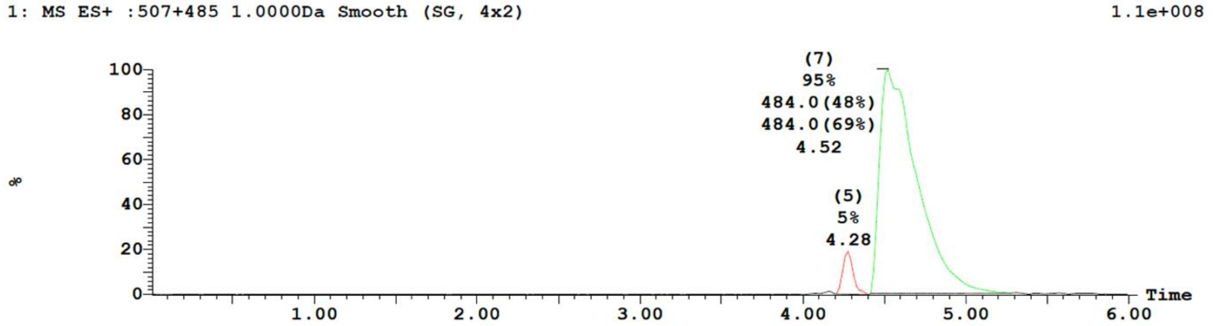

Peak Number	Compound	Time	AreaAbs	Area %Total	Width	Height	Mass Found
5		4.28	2e+006	5.14	0	2e+007	
7	Found	4.52	3e+007	94.86	1	1e+008	484.00, 484.00

Chromatographic purity analysis of compound **23**

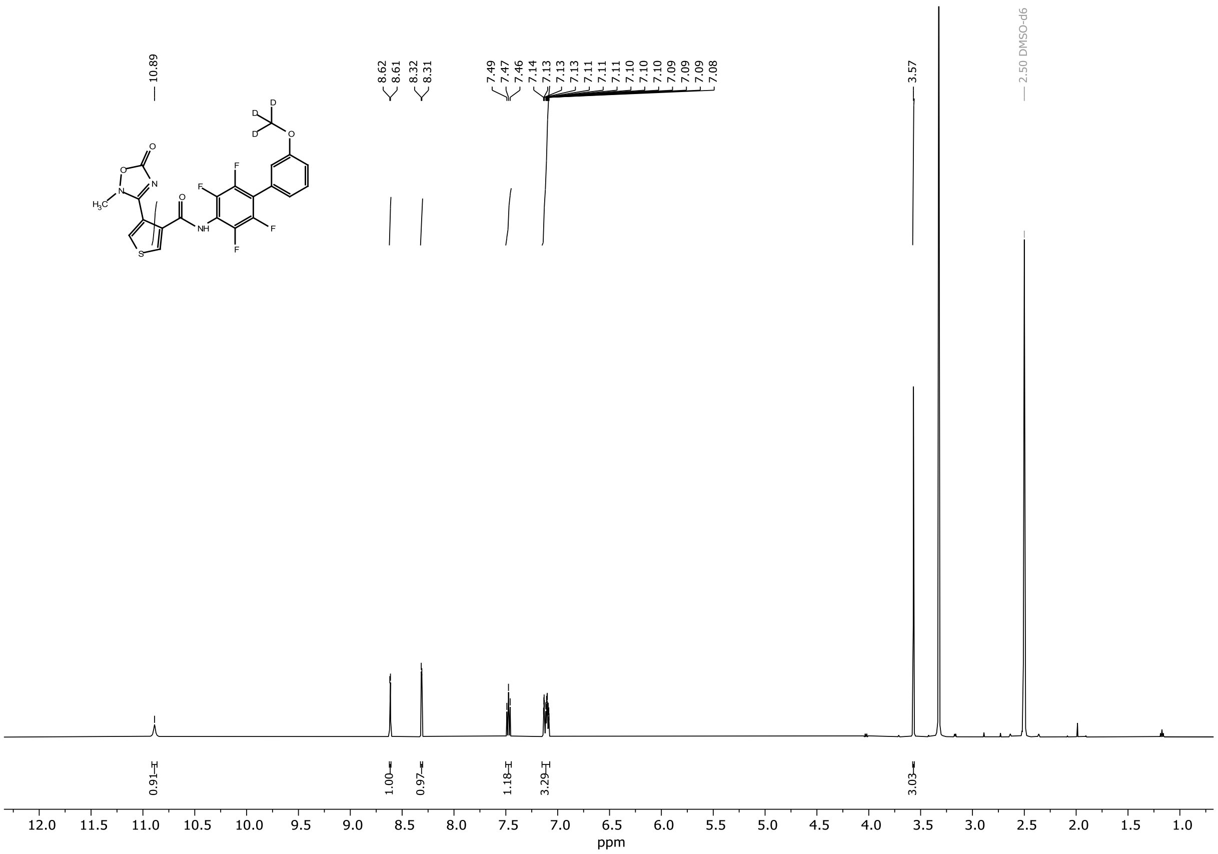

^{1}H-NMR (500 MHz, DMSO-d_6) of compound **24**

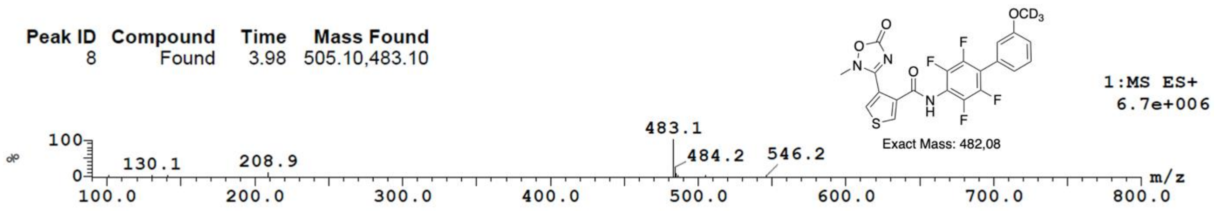

MS of compound **24**

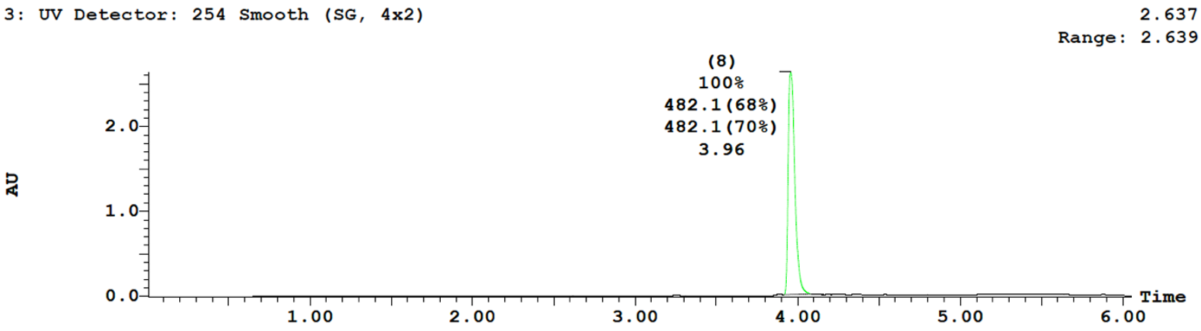

Peak Number	Compound	Time	AreaAbs	Area %Total	Width	Height	Mass Found
8	Found	3.96	1e+005	100.00	0	3e+006	482.10, 482.10

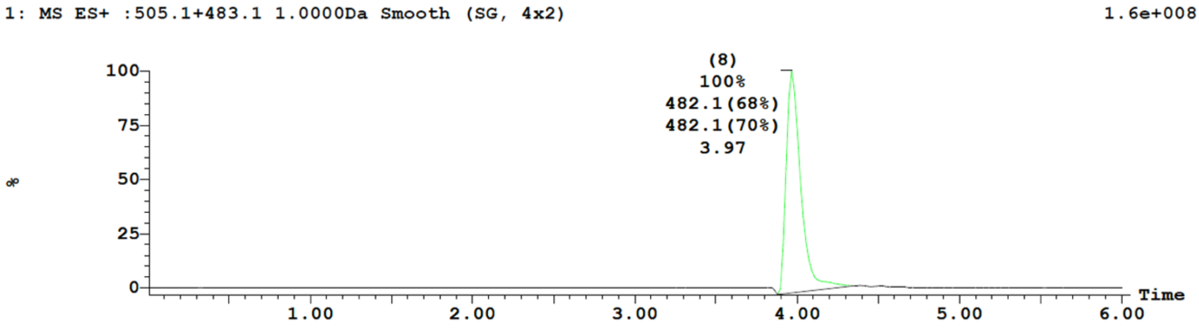

Peak Number	Compound	Time	AreaAbs	Area %Total	Width	Height	Mass Found
8	Found	3.97	2e+007	100.00	0	2e+008	482.10, 482.10

Chromatographic purity analysis of compound **24**

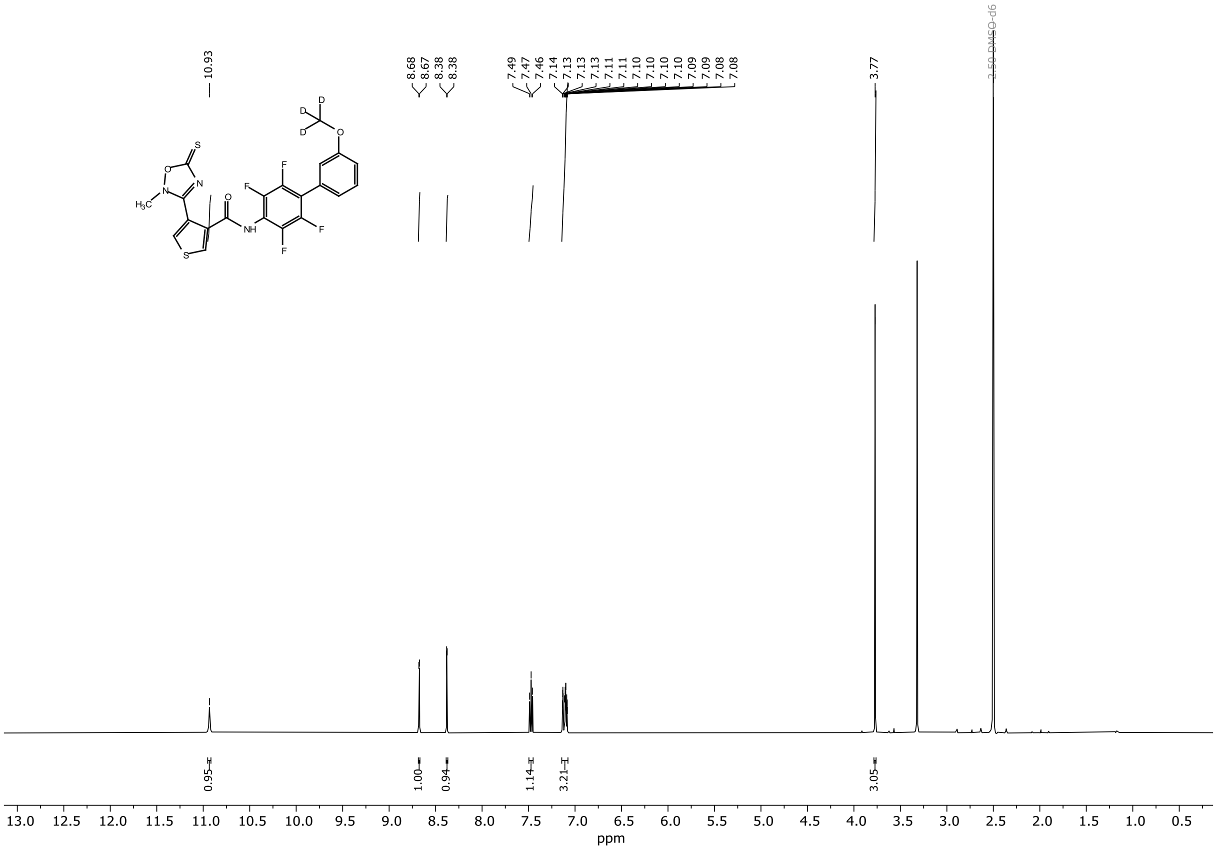

^{1}H-NMR (500 MHz, DMSO-d_6) of compound **25**

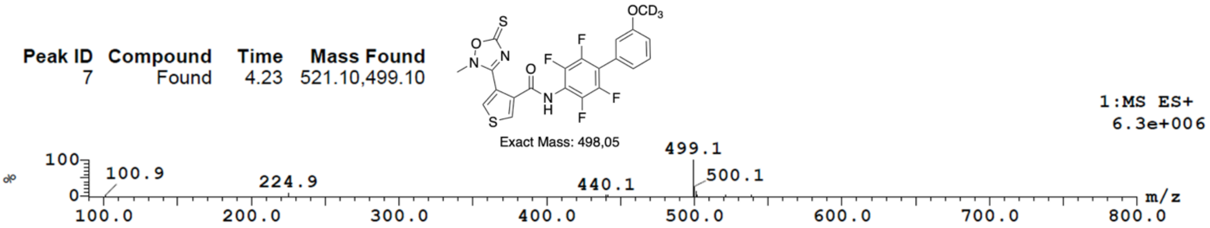

MS of compound **25**

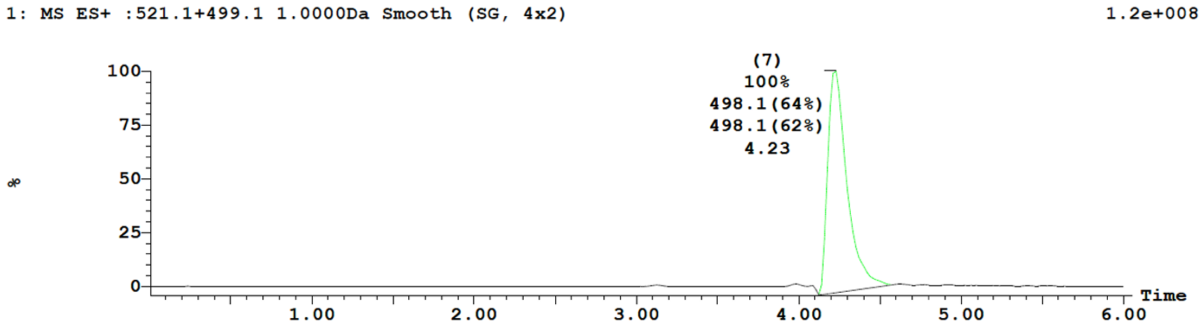

Peak Number	Compound	Time	AreaAbs	Area %Total	Width	Height	Mass Found
7	Found	4.23	2e+007	100.00	0	1e+008	498.10, 498.10

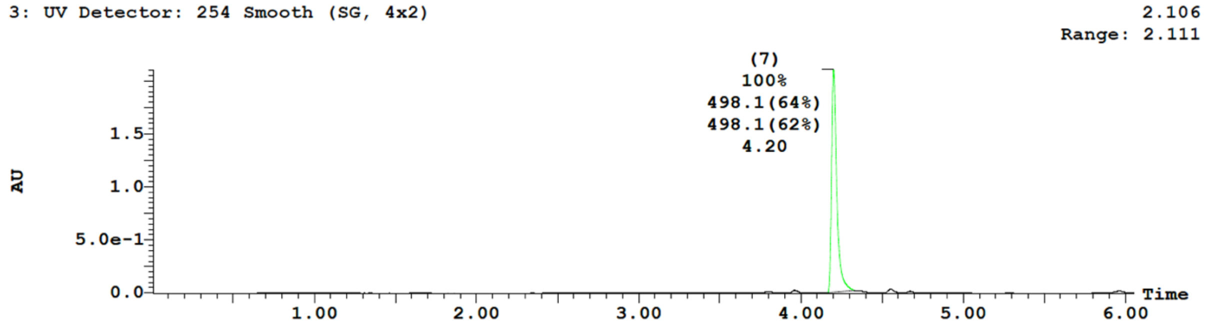

Peak Number	Compound	Time	AreaAbs	Area %Total	Width	Height	Mass Found
7	Found	4.20	8e+004	100.00	0	2e+006	498.10, 498.10

Chromatographic purity analysis of compound **25**

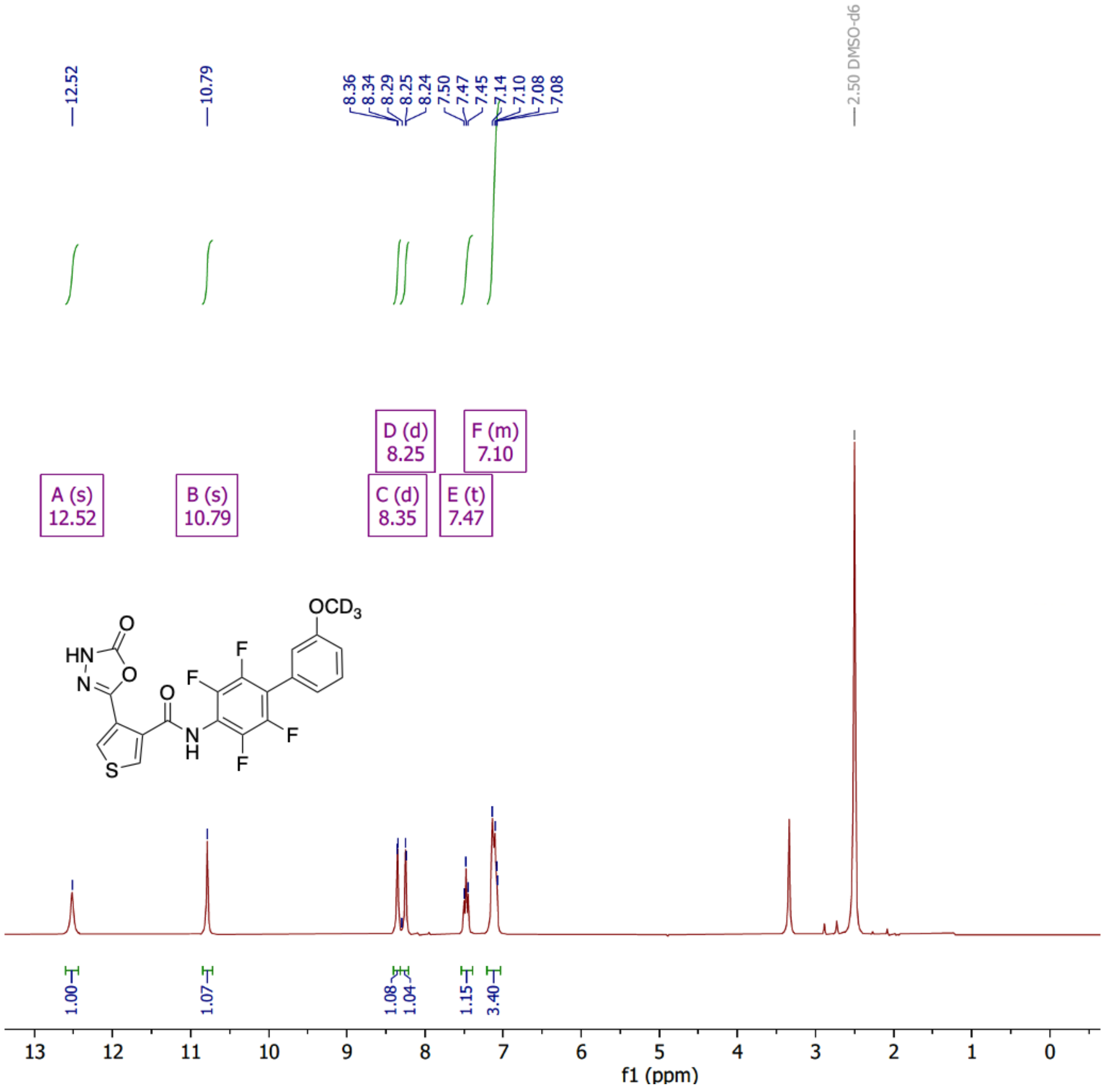

^{1}H-NMR (300 MHz, DMSO-d_6) of compound **26**

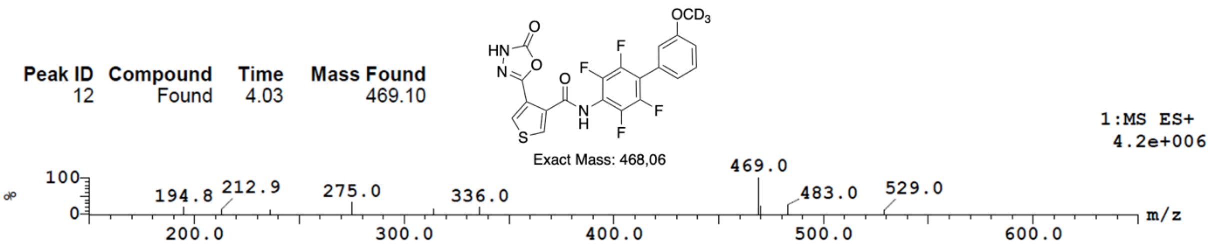

MS of compound **26**

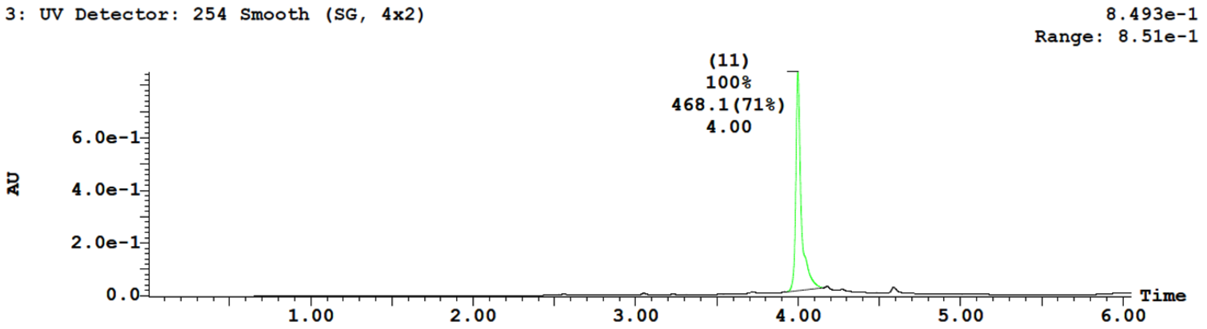

Peak Number	Compound	Time	AreaAbs	Area %Total	Width	Height	Mass Found
11	Found	4.00	3e+004	100.00	0	8e+005	468.10

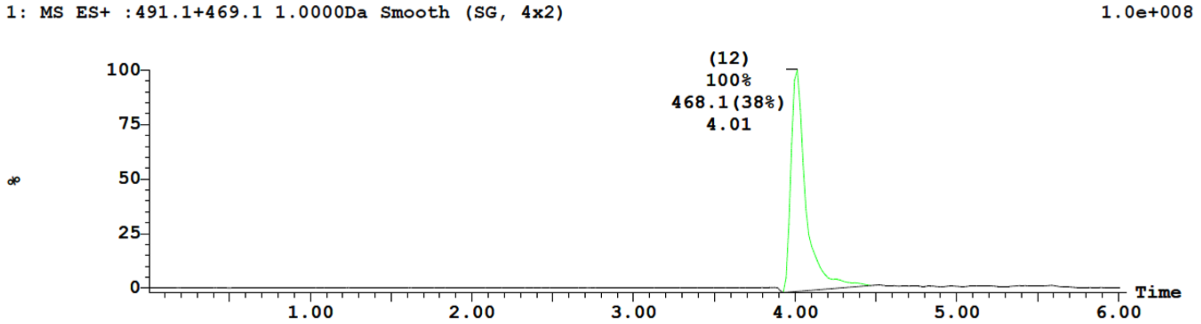

Peak Number	Compound	Time	AreaAbs	Area %Total	Width	Height	Mass Found
12	Found	4.01	1e+007	100.00	1	1e+008	468.10

Chromatographic purity analysis of compound **26**

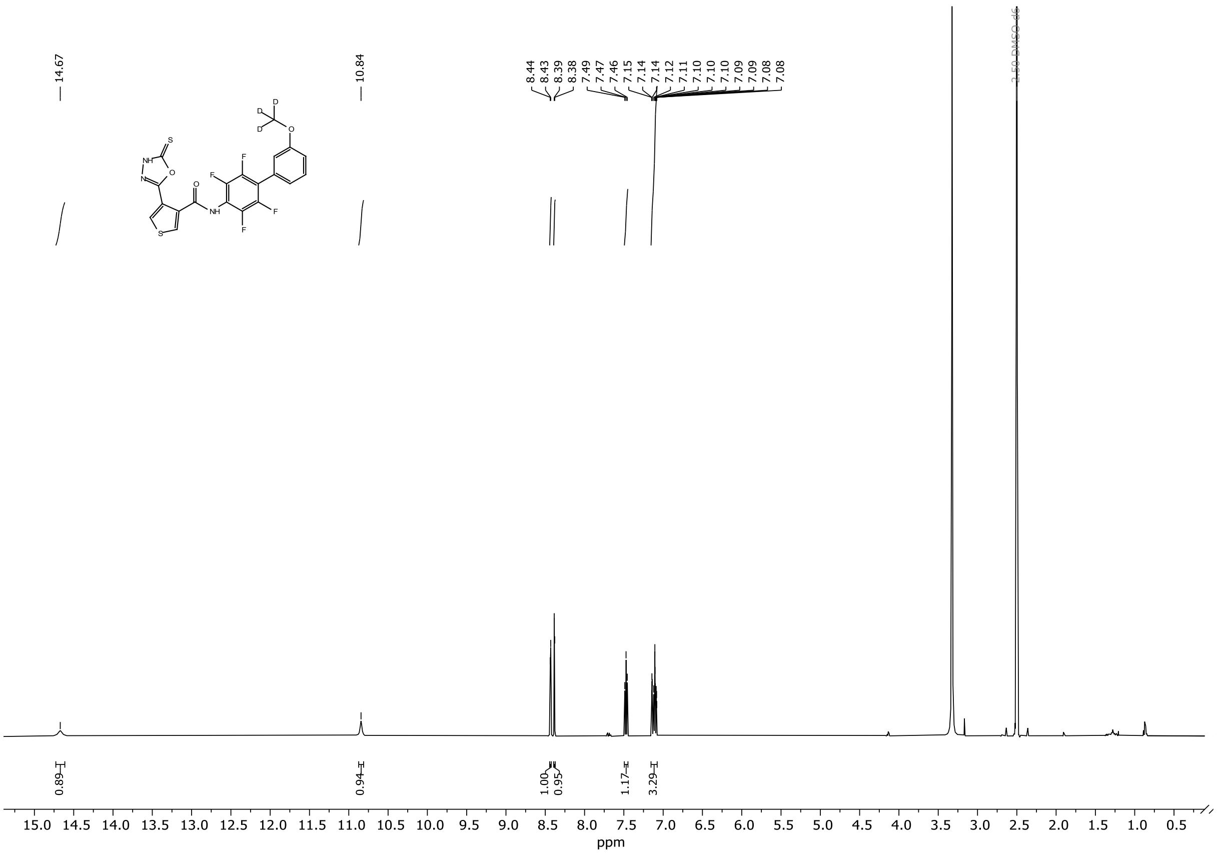

^{1}H-NMR (500 MHz, DMSO-d_6) of compound **27**

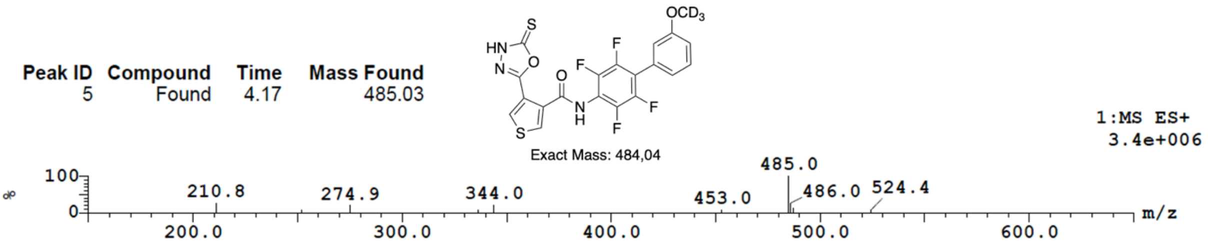

MS of compound **27**

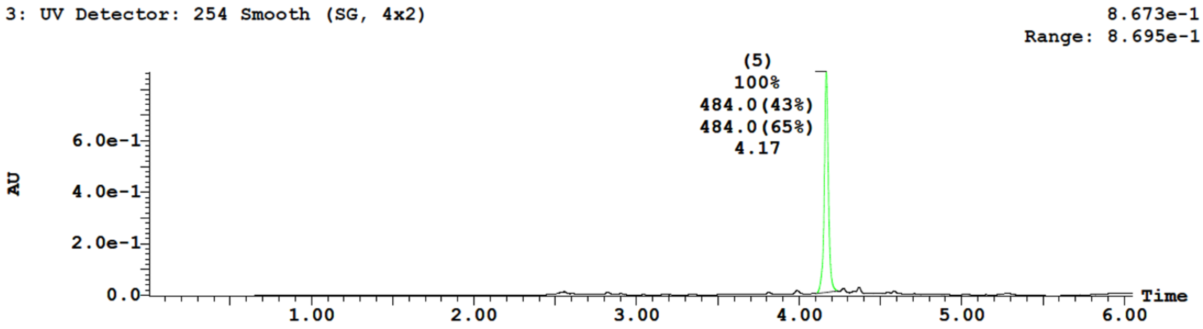

Peak Number	Compound	Time	AreaAbs	Area %Total	Width	Height	Mass Found
5	Found	4.17	2e+004	100.00	0	9e+005	484.03, 484.03

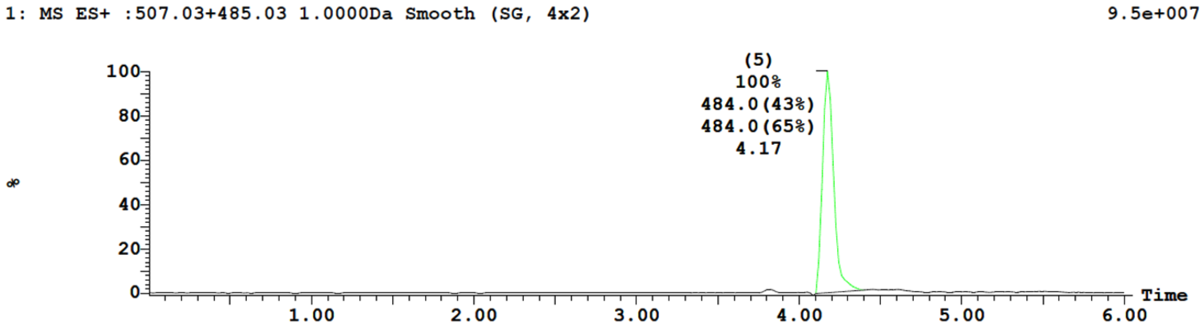

Peak Number	Compound	Time	AreaAbs	Area %Total	Width	Height	Mass Found
5	Found	4.17	8e+006	100.00	0	9e+007	484.03, 484.03

Chromatographic purity analysis of compound **27.**

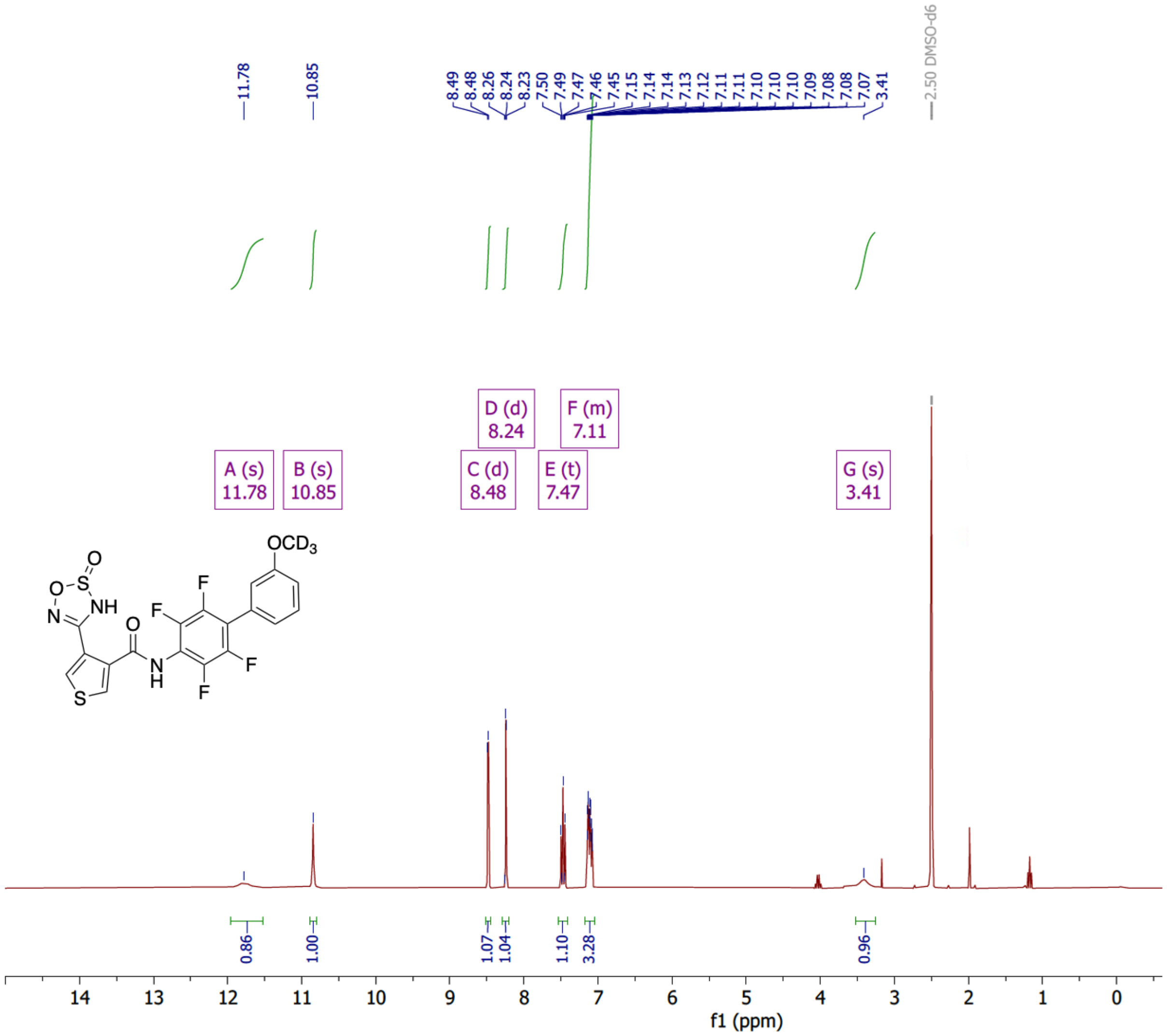

^{1}H-NMR (300 MHz, DMSO-d_6) of compound **28**

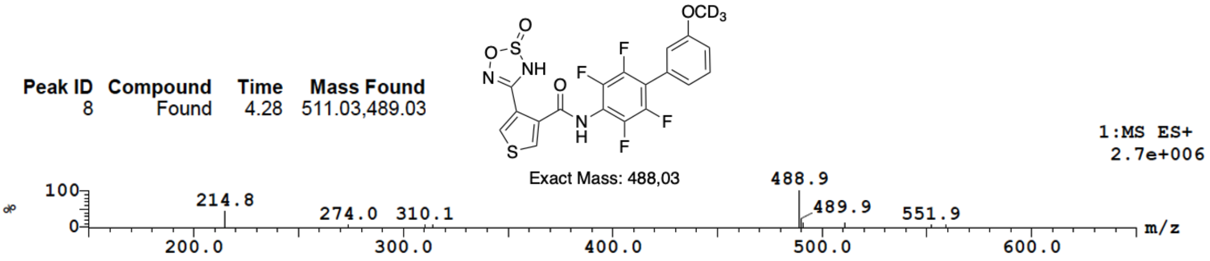

MS of compound **28**

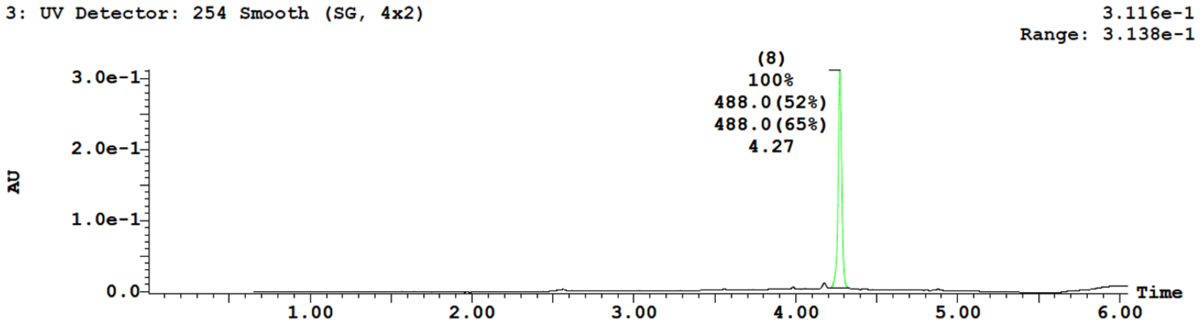

Peak Number	Compound	Time	AreaAbs	Area %Total	Width	Height	Mass Found
8	Found	4.27	8e+003	100.00	0	3e+005	488.03, 488.03

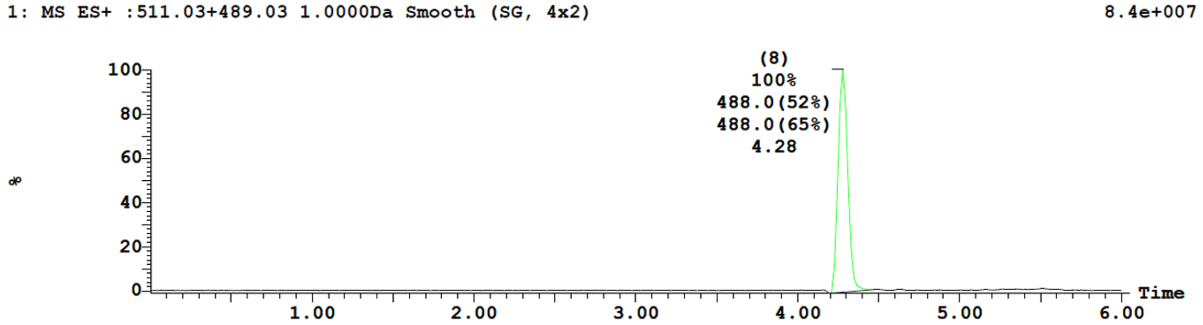

Peak Number	Compound	Time	AreaAbs	Area %Total	Width	Height	Mass Found
8	Found	4.28	6e+006	100.00	0	8e+007	488.03, 488.03

Chromatographic purity analysis of compound **28**

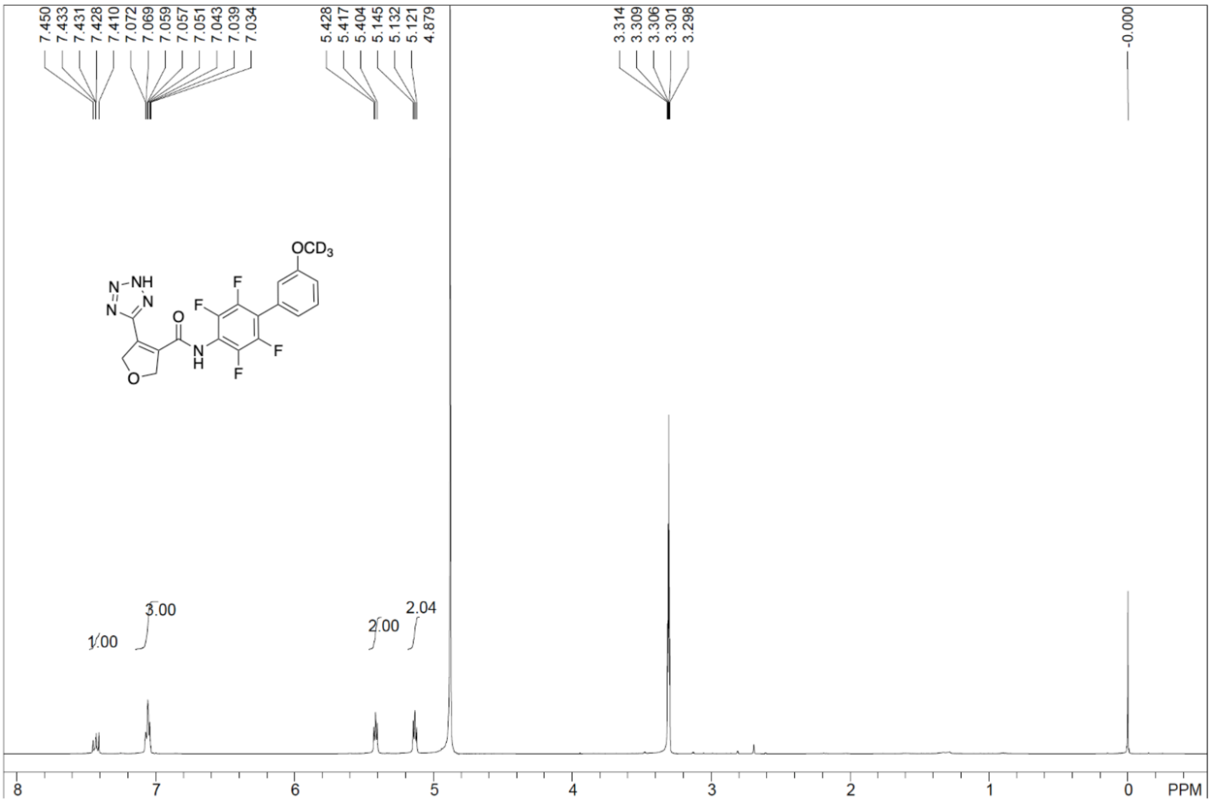

^{1}H-NMR (400 MHz, CD_3OD) of compound **30**

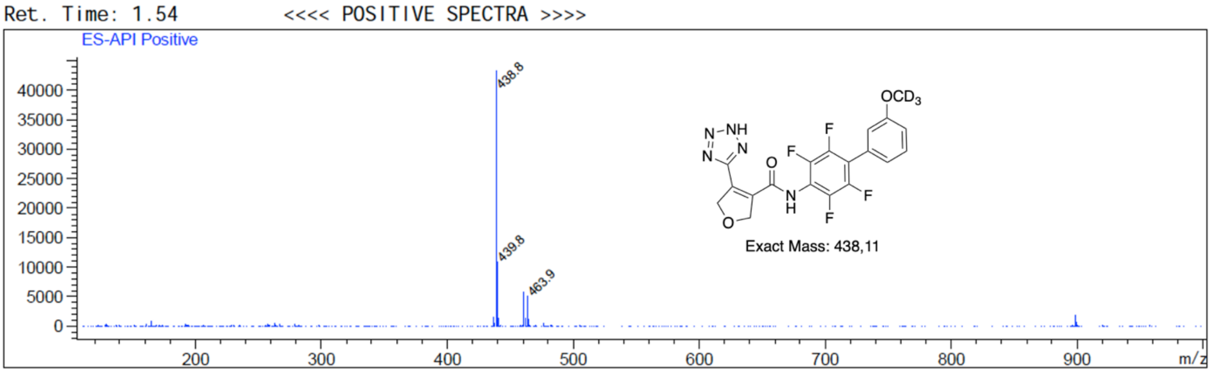

MS of compound **30**

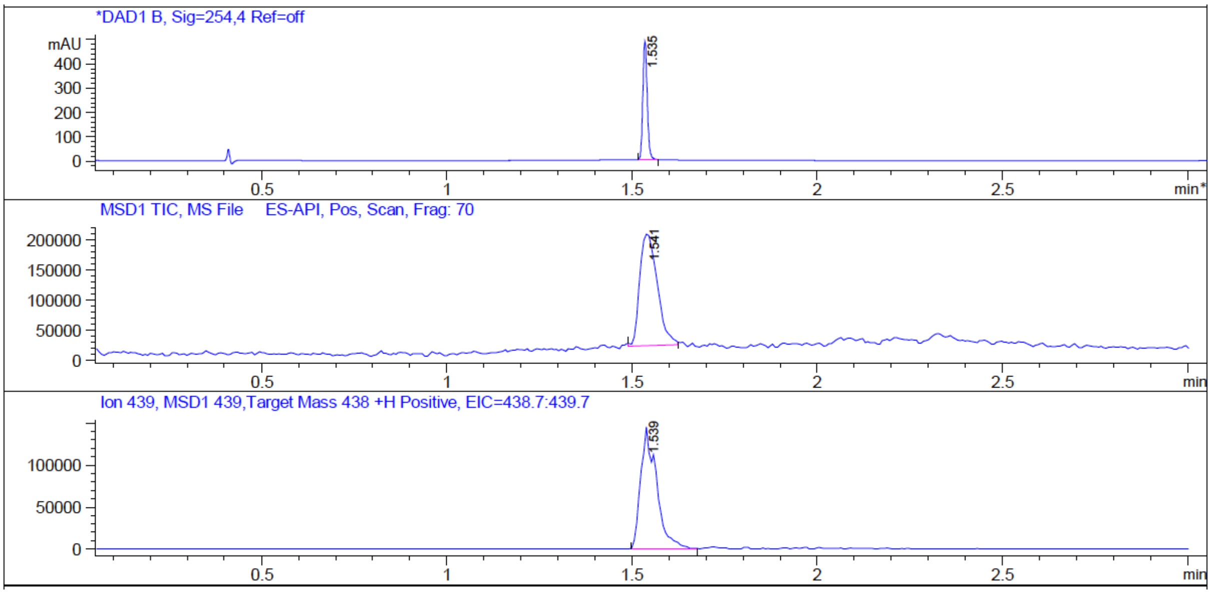

Chromatographic purity analysis of compound **30**

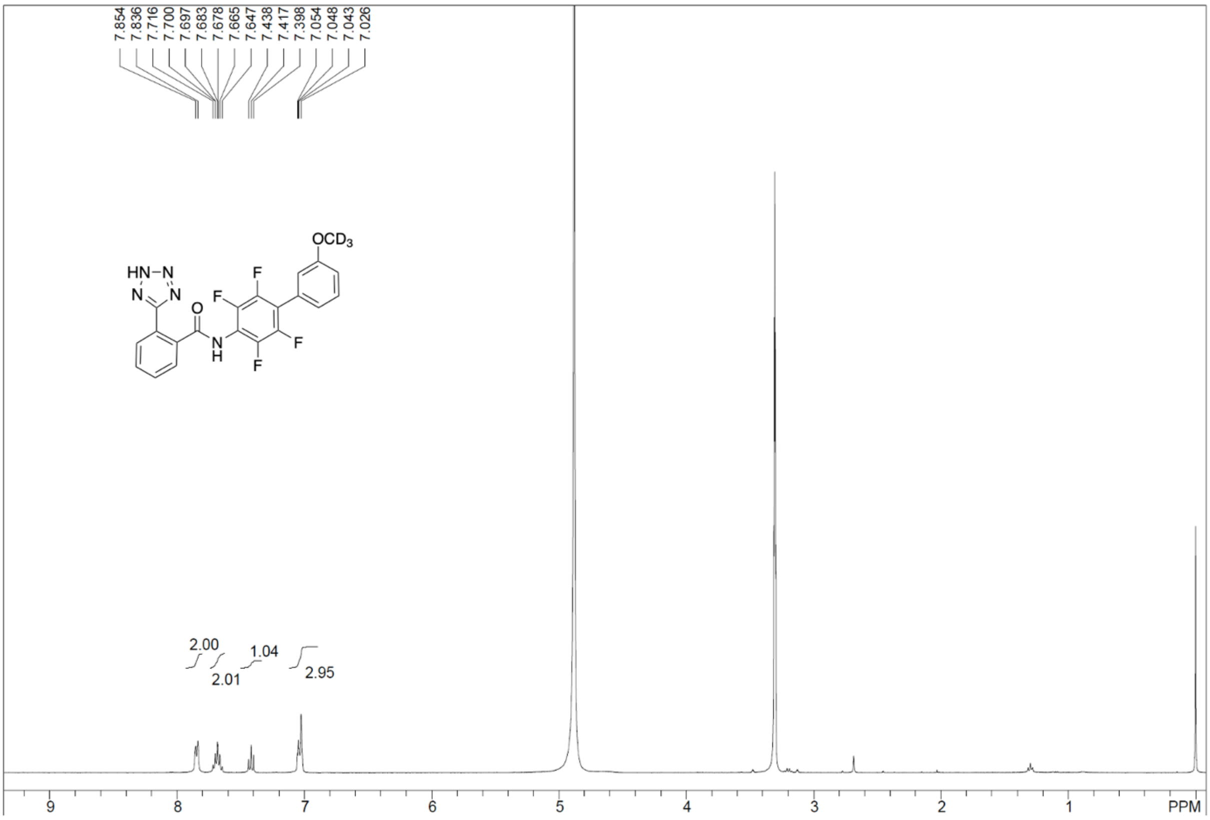

^{1}H-NMR (400 MHz, CD_3OD) of compound **31**

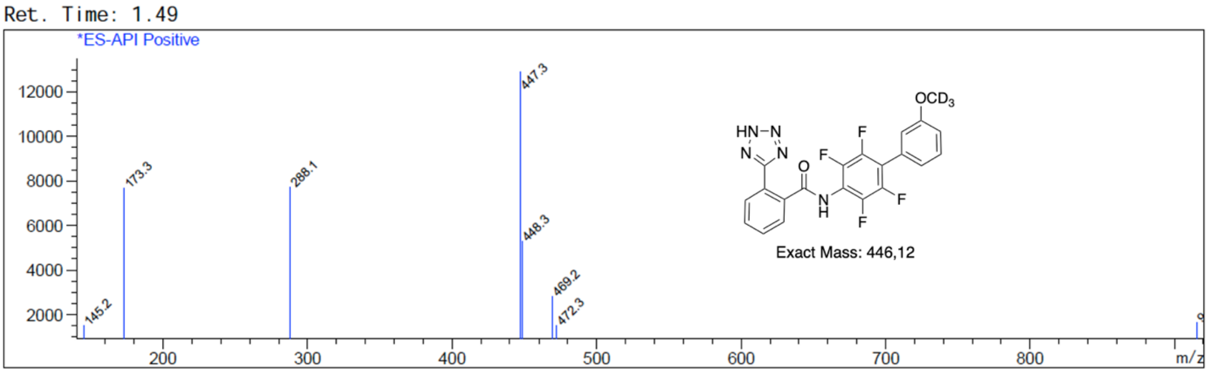

MS of compound **31**

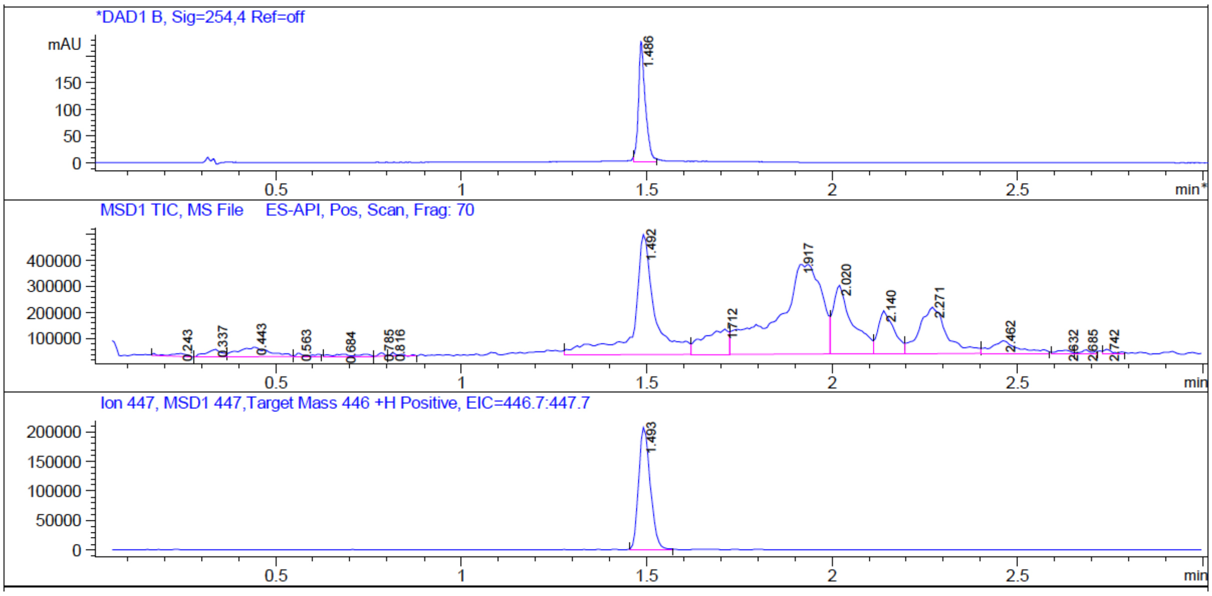

Chromatographic purity analysis of compound **31**

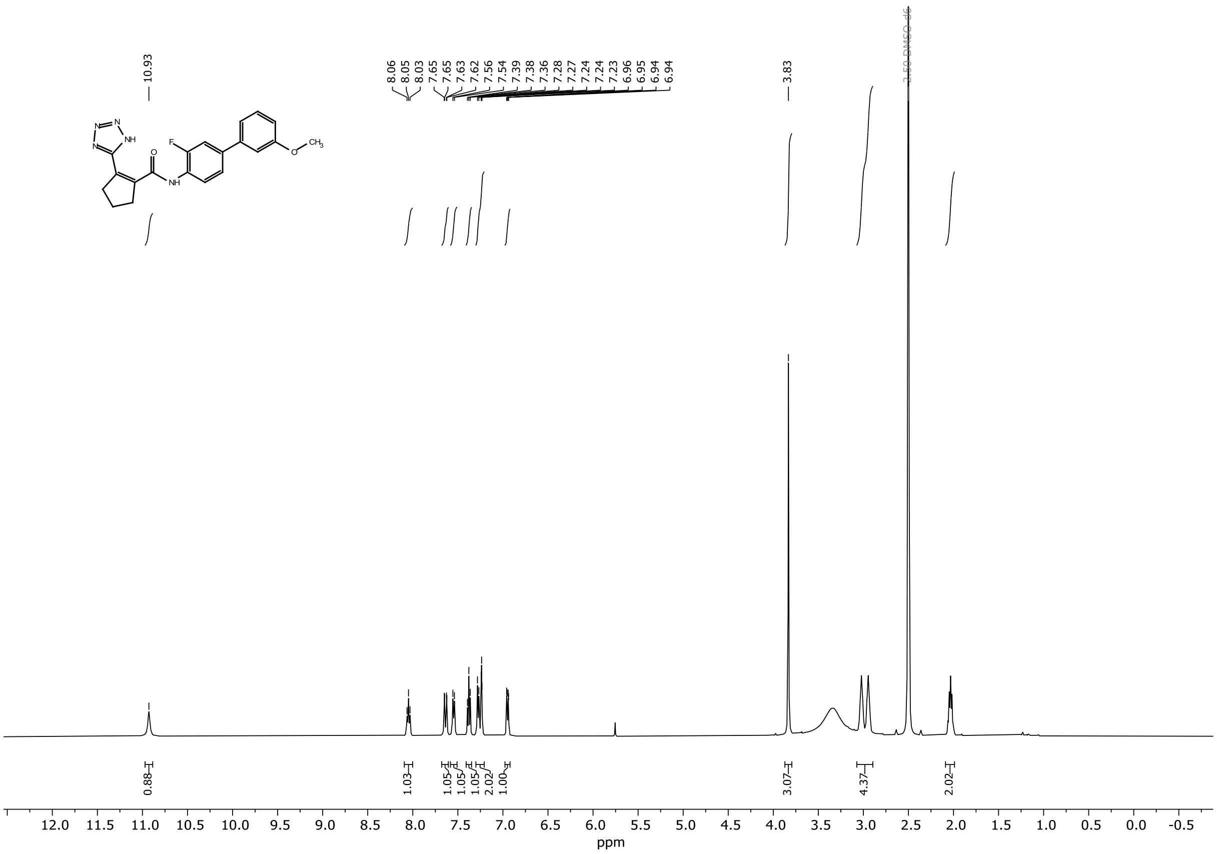

^{1}H-NMR (500 MHz, CD_3OD) of compound **32**

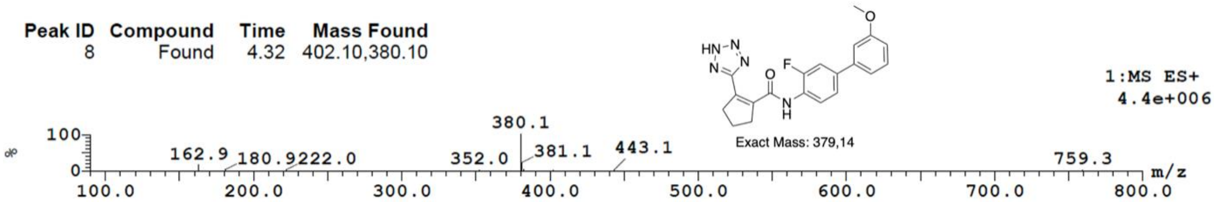

MS of compound **32**

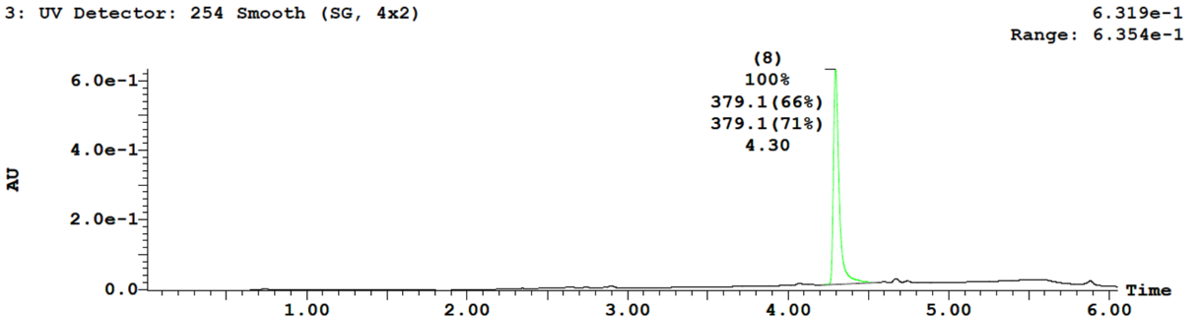

Peak Number	Compound	Time	AreaAbs	Area %Total	Width	Height	Mass Found
8	Found	4.30	3e+004	100.00	0	6e+005	379.10, 379.10

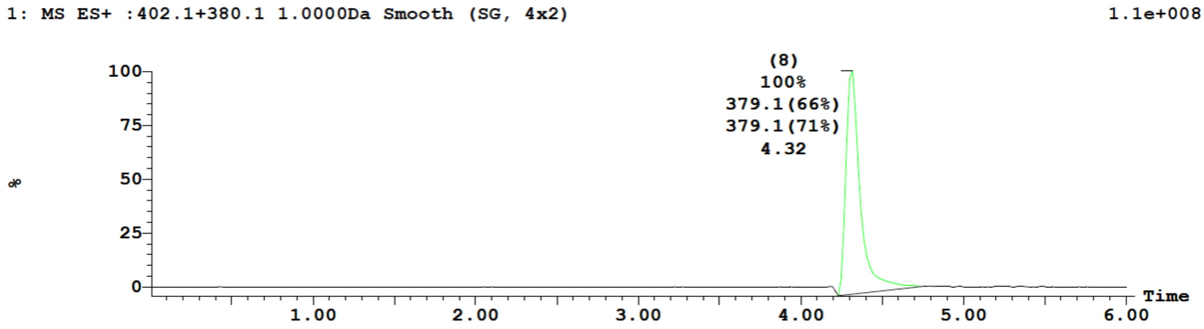

Peak Number	Compound	Time	AreaAbs	Area %Total	Width	Height	Mass Found
8	Found	4.32	1e+007	100.00	1	1e+008	379.10, 379.10

Chromatographic purity analysis of compound **32**
